# Stimulation of terrestrial ecosystem carbon storage by nitrogen addition: a meta-analysis

**DOI:** 10.1038/srep19895

**Published:** 2016-01-27

**Authors:** Kai Yue, Yan Peng, Changhui Peng, Wanqin Yang, Xin Peng, Fuzhong Wu

**Affiliations:** 1 Long-term Research Station of Alpine Forest Ecosystem, Provincial Key Laboratory of Ecological Forestry Engineering, Institute of Ecology and Forestry, Sichuan Agricultural University, Chengdu 611130, China; 2 Department of Biological Science, Institute of Environment Sciences, University of Quebec at Montreal, Montreal, Quebec, H3C 3P8, Canada; 3 Laboratory for Ecological Forecasting and Global Change, College of Forestry, Northwest A & F University, Yangling 712100, China; 4 State Key Laboratory of Geohazard Prevention and Geoenvironment Proctection, College of Environment and Civil Engineering, Chengdu University of Technology, Chengdu 610059, China

## Abstract

Elevated nitrogen (N) deposition alters the terrestrial carbon (C) cycle, which is likely to feed back to further climate change. However, how the overall terrestrial ecosystem C pools and fluxes respond to N addition remains unclear. By synthesizing data from multiple terrestrial ecosystems, we quantified the response of C pools and fluxes to experimental N addition using a comprehensive meta-analysis method. Our results showed that N addition significantly stimulated soil total C storage by 5.82% ([2.47%, 9.27%], 95% CI, the same below) and increased the C contents of the above- and below-ground parts of plants by 25.65% [11.07%, 42.12%] and 15.93% [6.80%, 25.85%], respectively. Furthermore, N addition significantly increased aboveground net primary production by 52.38% [40.58%, 65.19%] and litterfall by 14.67% [9.24%, 20.38%] at a global scale. However, the C influx from the plant litter to the soil through litter decomposition and the efflux from the soil due to microbial respiration and soil respiration showed insignificant responses to N addition. Overall, our meta-analysis suggested that N addition will increase soil C storage and plant C in both above- and below-ground parts, indicating that terrestrial ecosystems might act to strengthen as a C sink under increasing N deposition.

The anthropogenic nitrogen (N) input has been rising substantially since the onset of the Industrial Revolution, largely due to increased fertilizer use and the production of N oxides from combustion processes in vehicles and industry, and this input now exceeds natural biological N fixation^1^,^2^. Global annual N deposition has increased tenfold over the past 150 years and is predicted to double or even triple in the coming years^3^. The carbon (C) and N cycles in terrestrial ecosystems interact in numerous and complex ways^4^, and the C budget (i.e., C pools and fluxes) will likely play a more prominent role under recently projected increasing N deposition. The ways in which N enrichment influences C pools and fluxes within and across ecosystems will determine whether the increasing N input leads to an increase or decrease in terrestrial ecosystem C sequestration.

An altered ecosystem C cycle may exhibit positive or negative feedback to N enrichment and in return differently regulate N-enrichment effects. Soil C stocks are determined by the balance between plant growth in combination with the subsequent input of plant litter to the soil and C losses through soil respiration, litter decomposition, and dissolved organic C (DOC) export. It has been well documented that plant growth can be limited by N availability in some ecosystems^5^,^6^. Hundreds of N-fertilizer trials conducted in temperate and boreal forests have documented growth increases^7^,^8^, and aboveground net primary production (ANPP) is generally thought to increase with increasing N addition^9^. Additionally, N addition could reduce microbial biomass in many ecosystems, with corresponding declines in soil carbon dioxide (CO_2_) fluxes, suggesting a positive effect on soil C storage^10^. Such effects of N addition on soil C storage and CO_2_ emission were also found by Liu & Greaver^11^. Furthermore, N enrichment has been proven to increase plant litter quality by decreasing the lignin/N ratio and increasing the labile C substrate input to soil, thus resulting in greater C release through heterotrophic respiration during litter decomposition^12^. As a result, the simultaneously stimulated increase in both input (aboveground plant biomass) and output (CO_2_ emission) may not necessarily lead to an increase in C storage. Over a long-term period, soil C storage may even decrease due to an increased litter decomposition rate enhanced by increases in high quality plant litter^13^. Moreover, N enrichment could alter plant C allocation patterns, and plants may consequently allocate less C to belowground parts as a result of increased N availability^14^. Because of the slower decomposition rate of root litter compared with leaf litter^15^,^16^, less C allocation to the C pool in belowground plant parts under N addition may lead to a decrease in soil C storage over a long-term period.

N deposition could also alter the soil C pool by changing C effluxes such as DOC leaching and microbial respiration. The DOC concentration usually shows a positive response to chronic N addition^17^, thus increasing net ecosystem C loss through facilitating DOC leaching from the soil C pool relative to labile riverine and marine C pools^18^,^19^. However, N addition has been shown to have negative effects on microbial growth, decreasing the diversity of microbial community composition and activity^10^. Microbes consume organic C and release CO_2_ into the atmosphere through decomposition. Decreases in microbial abundance and activity would sustain greater ecosystem production, i.e., increasing soil C storage. Such diverse responses create a more complex and dynamic relationship between C budgeting and ecosystem N enrichment.

Recently, LeBauer & Treseder^7^, Xia & Wan^9^, and Knorr *et al.*^20^ have synthesized the effects of N addition on plant growth or litter decomposition, and Treseder^10^, Liu & Greaver^21^, Lu *et al.*^22^, and Janssens *et al.*^23^ have synthesized such effects on belowground C dynamics. However, it is still difficult to gain an overall perspective on the response of terrestrial ecosystem C cycle to N addition as a result of their different data sets compiling^21^,^22^. Moreover, some previous studies have suggested that N in combination with other nutrients exceed the effects of N alone on net primary production (NPP)^23^,^24^. In contrast, Fornara *et al.* found that C storage was minimally affected by the addition of multi-nutrient fertilizer, suggesting that the positive N-only effect on soil C storage could be reduced by the simultaneous addition of other nutrients^25^. Such paradoxical results make it important to synthesize the N-only effect on terrestrial C dynamics. Although some of the above-mentioned meta-analyses (e.g., Xia & Wan^9^) have noted such differences, many of these studies (e.g., Liu & Greaver^26^, Lu *et al.*^22^, and Zhou *et al.*^27^) assessed the effects of N addition on C dynamics based on results derived from a mixed database of both N-only and multi-nutrient applications, which may bias the evaluation of ecosystem C dynamics to N deposition.

Therefore, in the present study, we compiled data extracted from primary studies under experimental N addition across various ecosystems to quantitatively evaluate the central tendency of the N-enrichment effects on terrestrial ecosystem C dynamics, including C pools and fluxes. The main objective of this study was trying to gain an overall perspective on the effects of N deposition on terrestrial ecosystem C storage by evaluating how N-only addition may influence C pools and fluxes between aboveground and belowground compartments. Specifically, the responses of C pools (including plant, litter, microbial biomass, soil, and DOC) and the C influx and efflux to experimental N addition were evaluated. Previous studies have suggested that environmental factors (e.g., temperature and latitude), experimental duration, and ecosystem type are important moderators for the response of the C cycle to N addition^22^,^26^,^27^; therefore, we assessed the effects of climatic conditions, including mean annual temperature (MAT), mean annual precipitation (MAP), latitude, and ecosystem types, on the response of the C cycle to N addition. Treatments using mixed fertilizers were not included in our study, but we discuss our results in the context of previously published works reporting such case studies such as Liu & Greaver^21^ and Fornara *et al.*^25^.

## Results

### Carbon pools

**Figure 1:**
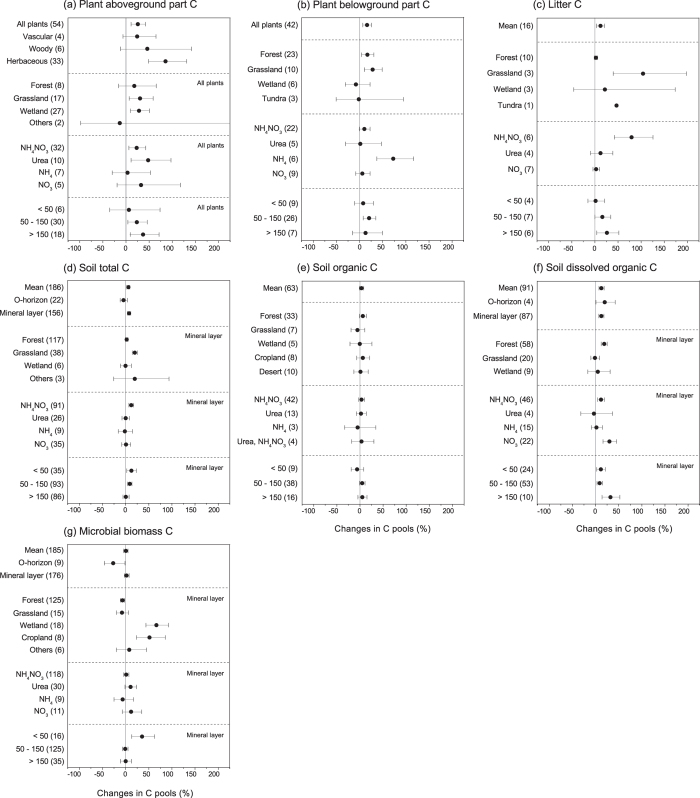
Effects of N addition on ecosystem C pools of (a) plant aboveground parts C, (b) plant belowground parts C, (c) plant litter C, (d) soil total C, (e) soil organic C, (f) soil dissolved organic C, and (g) soil microbial biomass C expressed as the percentage change relative to the control (%). The values are means ±95% CIs, and the number of case studies is shown in parentheses. The text in the upper right of some of the figures indicates the database used in the meta-analysis.

The overall effects of N addition on the plant C pool were found to be stimulative (Fig. 1a, b). Compared with the control groups, the C content of the aboveground plant parts was significantly increased by an average of 25.65% ([11.07%, 42.12%], 95% CI, the same below) under N-addition conditions across all ecosystems (Fig. 1a). When the effects of the moderators were considered, the effects of N addition were only significant in the grassland and wetland ecosystems, where the C contents of the aboveground plant parts were increased by 30.46% [7.12%, 58.88%] and 28.20% [9.43%, 50.19%], respectively. With respect to the different N chemical forms and addition levels, the effects of NH_4_NO_3_ (+23.16% [6.19%, 42.85%]) and urea (+47.82% [10.86%, 97.11%]) appeared to be more pronounced than the effects of the other forms, and higher levels of addition appeared to result in a greater positive effect on the C contents of the aboveground plant parts than did lower addition levels. Similarly, N addition also increased the C content of the belowground plant parts by 15.93% [6.80%, 25.85%] (Fig. 1b), and the stimulation was most pronounced for grasslands (+28.24% [10.41%, 48.96%]). However, the positive impact was significant only for NH_4_ and an intermediate level of N addition (50–150 kg N/ha/yr). Additionally, N addition significantly increased plant litter C by 12.16% [3.81%, 21.19%] (Fig. 1c).

Soil total C content was increased by 5.82% [2.47%, 9.28%] under N addition (Fig. 1d). For the response of the mineral layer within different ecosystems, significantly positive effects were observed only in grassland (+19.75% [14.40%, 25.33%]). NH_4_NO_3_ was the most significant form of added N, increasing soil total C by 12.01% [7.11%, 17.13%]. In contrast, N addition showed insignificant effects on SOC, regardless of the effects of moderators (Fig. 1e). N addition significantly increased the soil DOC content by an average of 12.63% [7.30%, 18.20%] across all studies, with increases of 19.69% [0.60%, 42.40%] and 12.32% [6.87%, 18.06%] for the O-horizon and mineral layer, respectively (Fig. 1f). When the data were subdivided into different ecosystems, only forest (+18.48% [12.03%, 25.29%]) showed significantly positive responses. The soil DOC concentration was significantly increased by NH_4_NO_3_ and NO_3_ as well as all of the levels of N addition. Microbial biomass C (MBC) showed an insignificant response to N addition across all of the case studies (Fig. 1f). Considering the effects of the moderators, MBC presented a significantly negative response to N addition in forest (−6.95% [−11.58%, −2.07%]) but significantly positive responses to N addition in wetland (+66.36% [43.76%, 92.51%]) and cropland (+51.30% [23.34%, 85.58%]).

### Carbon fluxes

**Figure 2:**
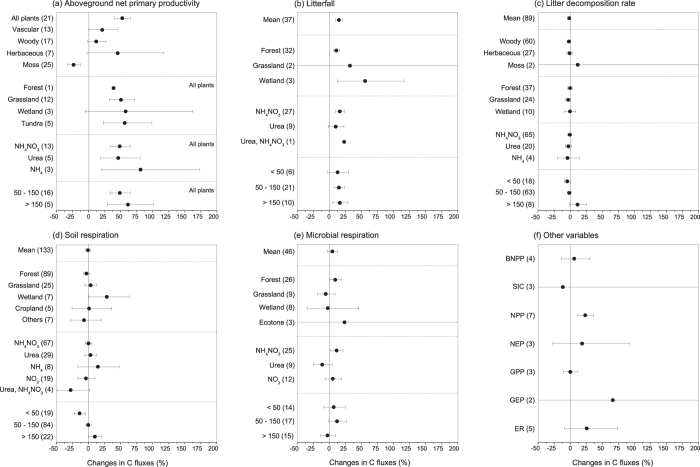
Effects of N addition on ecosystem C fluxes: (**a**) aboveground net primary productivity, (**b**) litterfall, (**c**) litter decomposition rate, (**d**) soil respiration, (**e**) microbial respiration, and (**f**) other parameters related to C fluxes, which are expressed as the percentage change relative to the control (%). The values are the means ±95% CIs, and the number of case studies is shown in parentheses. The text in the upper right of some of the figures indicates the database used in the meta-analysis. BNPP: belowground net primary productivity; NPP: net primary production; SIC: soil inorganic C; NEP: net ecosystem productivity; GPP: gross primary productivity; GEP: gross ecosystem productivity; ER: ecosystem respiration.

Overall, N addition significantly increased ANPP by an average of 52.38% [40.58%, 65.19%] (Fig. 2a). This positive effect was significant for grassland (+50.38% [32.17%, 71.12%]) and tundra (+56.27% [23.06%, 98.44%]) within the different ecosystems and for all N forms and addition levels. Furthermore, litterfall showed a significantly positive response to N addition, exhibiting an average increase of 14.67% [9.24%, 20.38%] (Fig. 2b). Within the different ecosystems, significantly positive effects were observed in forest (+10.76% [5.88%, 15.87%]) and wetland (55.63% [12.15%, 115.96%]). Litterfall was significantly increased by NH_4_NO_3_ (+16.10% [9.48%, 23.13%]), and the effects of intermediate (50–150 kg N/ha/yr) and high (>150 kg N/ha/yr) addition levels were more pronounced compared with the effect of a low addition level (<50 kg N/ha/yr). However, N addition had no significant impact on litter decomposition rate across all case studies (Fig. 2c).

N addition had no significant influence on soil respiration across all case studies, and a significant effect was observed only in wetland (+28.26% [0.27%, 64.07%]) (Fig. 2d). Similarly, microbial respiration showed an insignificant response to N addition as a whole (Fig. 2e). However, when considering the different ecosystems, a significantly positive effect was observed in forest (+9.08% [0.70%, 18.15%]) (Fig. 2e). With respect to the other variables related to the carbon cycle, only NPP showed a significantly positive response to N addition (Fig. 2f).

### Influence factors and sensitivity analysis

**Figure 3:**
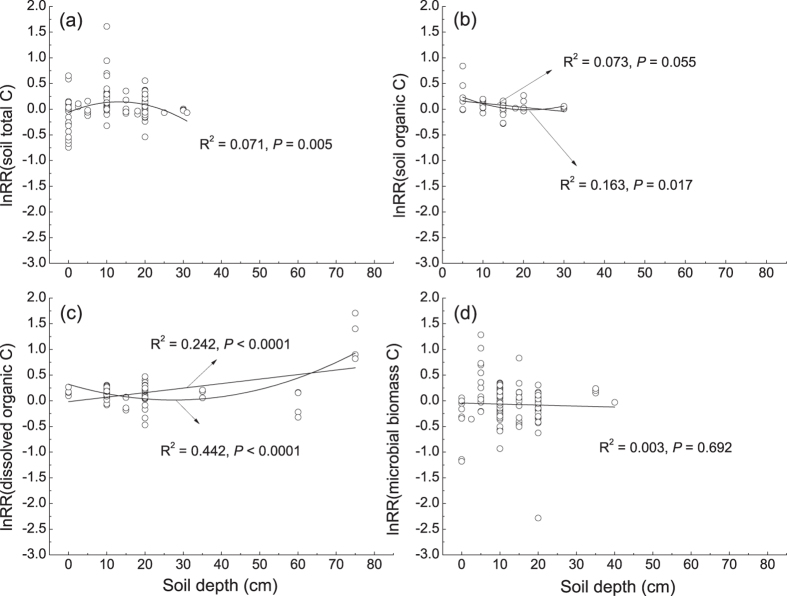
Relationships of soil depth with the response ratio (lnRR) of (**a**) soil total C, (**b**) soil organic C, (**c**) soil dissolved organic C, and (**d**) soil microbial biomass C. Zero represents the O-horizon.

**Figure 4:**
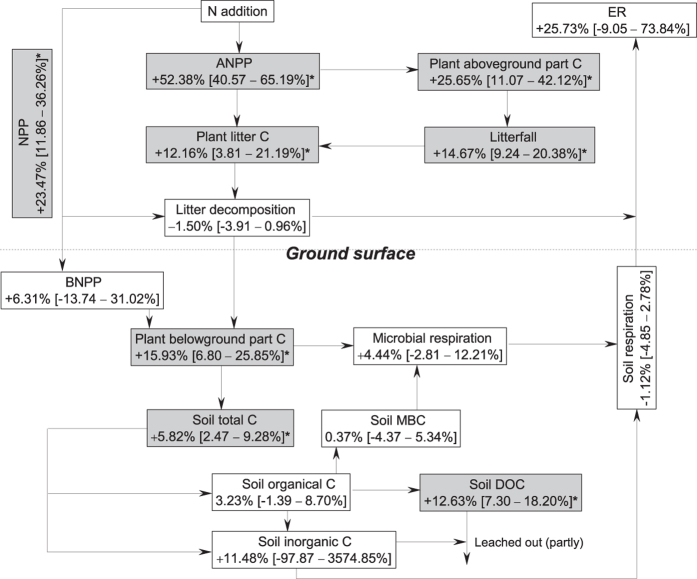
The responses of the terrestrial ecosystem carbon cycle to experimental N addition with the changes imposed across all case studies. Numbers indicate the mean changes in the C pool or flux with the 95% CI. Asterisks indicate statistical significance (*P* < 0.05). NPP: net primary production; ANPP: aboveground net primary production; BNPP: belowground net primary production; MBC: microbial biomass carbon; TOC: total organic carbon; DOC: dissolved organic carbon; ER: ecosystem respiration.

Soil depth showed significant influences on the response of soil total C, SOC, and DOC (Fig. 3a–c) but not on MBC (Fig. 3d). The regression analyses suggested that N-addition level, experimental duration, MAT, MAP, and latitude can also be important moderators of the response of terrestrial C dynamics to N addition (Table 1). As a whole, terrestrial ecosystem C storage may be increased by N addition, as both plant C and soil C were significantly stimulated, whereas C fluxes were minimally affected (Fig. 4). Moreover, the sensitivity analysis suggested that when only a mean effect size per study was considered, the results did not change qualitatively regarding the response of all the components of C cycles (C pools and fluxes) to N addition (Suppl. Table S1).

## Discussion

Experimental N addition was found to stimulate the growth of both above- and below-ground plant parts, indicating an increase in the size of the plant C pools (Fig. 1a,b). These results is consistent with a previous meta-analysis investigating the effects of N addition on plants conducted by Xia & Wan^9^ who noticed the differences between N-only and multi-nutrient addition effects. The net C accumulation in plants can be attributed to the stimulation of ANPP and NPP under N addition (Fig. 2a,f). N availability can be an important limitation for plant NPP in some ecosystems^7^; therefore, the significant increase in soil N availability induced by N addition can enhance plant N uptake, which in turn increases leaf N contents and plant photosynthesis and ultimately stimulates shoot and root growth^5^,^28^. The aboveground litter C pool and litterfall increased significantly in response to N addition (Figs 1c and 2b), but the litter decomposition rate showed insignificant responses to N addition (Fig. 2c). Aboveground litterfall usually increases following N addition^7^, which can alleviate C limitation as litter becomes incorporated into the soil. N may increase, decrease, or have no effect on the litter decomposition rate, depending on both internal (e.g., litter substrate quality) and external (e.g., environmental conditions and microbial physiology) factors related to decomposition^29^. The insignificant effects of N addition on litter decomposition observed in our study are consistent with a previous meta-analysis that did not identify any significant effects of N on litter decomposition^20^.

In addition, soil total C and DOC were significantly altered by experimental N addition (Fig. 1d,f), but SOC showed insignificant responses to N enrichment (Fig. 1e). These results were inconsistent with those found by Liu & Greaver^26^, who suggested that soil C and DOC showed insignificant response to N addition. Such differences may be attributed to the mixture data sets they used, as Fornara & Tilman^30^ found that soil C was significantly increased by N-only addition but not multi-nutrient in an experiment involving 27 years of chronic N addition. An increase in the ecosystem C pool increases the amount of C available for leaching, which can contribute to an increased DOC concentration. Additionally, the response of the DOC pool is influenced by the form of N addition as the addition of NH_4_NO_3_ and NO_3_ increased the DOC concentration by an average of 11.67% [4.89%, 18.90%] and 29.77% [15.99%, 45.18%], respectively, whereas the addition of NH_4_ showed an insignificant effect (Fig. 1f). NO_3_ has been found to increase DOC, whereas NH_4_ addition usually decreases DOC^18^; this difference has been attributed to their different influences on soil acid^18^,^19^. In contrast to the response of soil DOC to N addition, SOC showed an insignificant response (Fig. 1e). Soil microbes consume organic C and convert it into either atmospheric CO_2_ or dissolved CO_2_ in a soil solution. This finding is consistent with the response of MBC and microbial respiration as N addition had insignificant impacts on both MBC (Fig. 1g) and microbial respiration (Fig. 2e).

Sylvia *et al.* found that an increasing fresh litter input generally resulted in an increase in MBC, primarily due to an increase in the available labile C input from fresh litter^31^. However, this correlation could be affected by N addition as a result of potential mechanisms such as toxicity effects caused by N enrichment and inconsistent changes in individual C components and the total labile C input^26^. The MBC response observed in the present study is inconsistent with the results of Liu & Greaver^26^ and Treseder^10^, who indicated that N addition decreased MBC by approximately 20% and 15%, respectively. This disparity between these previous studies and the present one may be attributed to the different data sets compiled as these previous authors included case studies using multi-nutrient addition [e.g., N with P and/or K addition] in their analyses^26^, which further indicated that N-only and multi-nutrient additions can have different effects on soil and microbial biomass C storage. As the availability of water, P, belowground C and nutrient sources all represent potential limitations for microbial biomass^32^,^33^,^34^, MBC could thus show different responses between N-only and multi-nutrient addition^25^. Similar to microbial respiration, soil respiration was not significantly altered by N addition across all case studies (Fig. 4b). A recent meta-analysis conducted by Zhou *et al.* suggested that N addition could significantly increase soil respiration by 2.0% across all ecosystems^27^. However, in their research, case studies using multi-nutrient application were included, and such inclusion may explain the different results between their study and the present work. More importantly, as Zhou *et al.* found that different ecosystems could show different responses of soil respiration to N addition^27^, the different ecosystem types considered between our study and theirs could explain the different results.

The response of soil C dynamics to experimental N addition can differ considerably among ecosystems. We found that experimental N addition significantly increased the aboveground plant part C pools in grassland and wetland but not forest (Fig. 1a). The latter result can be attributed to the small sample size for forest (Fig. 1a), which may have limited the statistical power to detect a significant effect. The significant increases in ANPP and litterfall induced by N addition and the unaffected litter decomposition rate all increase aboveground litter C accumulation and thereby enhance the size of the C pool in litter (Fig. 1c). The effect of N addition on soil MBC (Q_*between*_ = 81.60, *P* < 0.0001) varied among different ecosystems, and N addition significantly increased MBC in wetlands by 66.36% [43.76%, 92.51%] (Fig. 1g). Compared with other ecosystem types, forest exhibited the most significant response to N addition for SOC and DOC (Fig. 1e,f). These results highlight the need to consider the ecosystem type as an important moderator when predicting the response of the C cycle to increased N deposition under a scenario of climate change. Moreover, the small sample sizes for variables such as net primary productivity (EPP), net ecosystem productivity (NEP), and ecosystem respiration (ER) also limited comprehensive analyses (Fig. 2f).

Forcing factors such as the form of added N (e.g., NH_4_NO_3_ or urea), magnitude of N addition, duration of the experiment, and soil depth may be potentially important moderators influencing the response of ecosystem C cycles to experimental N addition. NH_4_NO_3_ and urea were the most widely used N fertilizers in the N-addition experiments (Suppl. Table S1); thus, the sample sizes for these two forms of N were generally very robust. Different forms of N can influence soil properties, such as soil acidity^18^, in various ways and therefore exert diverse impacts on soil C cycles. For example, NH_4_NO_3_ and urea showed different impacts with respect to C pools (Fig. 1) and C fluxes (Fig. 2), in both magnitude and direction. As discussed above, the addition of NH_4_NO_3_ and NO_3_ can increase soil acidity and therefore the DOC concentration, whereas NH_4_ usually exerts a mild influence^18^. Furthermore, the addition of organic N, such as urea, can simultaneously increase the input of C, which could also confound the effect of N. Moreover, the soil nutrient status can influence the impact of N addition. For example, the addition of NO_3_ in the form of calcium nitrate can enhance the effect of N on MBC in soils that are calcium limited^35^. The fertilizer addition rate could also moderate the effects of N addition, as both C pools and fluxes generally showed significant responses to intermediate and/or high levels of N addition. However, the regression analysis suggested that only MBC in the soil O-horizon and litter decomposition rate were significantly correlated with N-addition level (Table 1). Similarly, the experimental duration impacted the responses of the C pools significantly: both soil total C and MBC showed significant correlations with experimental duration (Table 1). Moreover, soil depth has been demonstrated to be an important moderator of the response of soil C dynamics to changing environmental factors, such as land-use changes^36^ and N enrichment^37^,^38^. Our study found that soil total C, SOC and DOC correlated significantly with soil depth (Fig. 3a–c). However, soil depth had an insignificant impact on soil MBC (Fig. 3d).

Moreover, environmental factors such as MAT, MAP, and latitude may potentially influence the responses of ecosystem C pools and fluxes to experimental N addition. The N addition-induced changes in the C content of MBC (R^2^ = 0.045, *P* = 0.005) and litter decomposition rate (R^2^ = 0.078, *P* = 0.008) were significantly correlated with MAP, whereas only MBC was significantly correlated with latitude (R^2^ = 0.038, *P* = 0.009; Table 1), indicating that increased precipitation could enhance the effects of N addition on MBC and litter decomposition. The results indicated that MAP plays an important role in modulating the effects of N addition on ecosystem C dynamics. However, the effect of MAT on the response of C dynamics to N addition was insignificant (Table 1), which may be due to the effects of confounding factors among study sites, such as soil condition and plant species composition^39^,^40^. LeBauer & Treseder^7^ found that the response of NPP to N addition was correlated with MAT, MAP, and latitude within but not among ecosystems, and such variation may have contributed to some of the insignificant correlations that we observed between the C dynamic response ratio and the environmental factors. However, because of the small sample sizes for some of the variables within each type of ecosystem, we did not analyse the correlations between the C dynamics response ratio and the environmental factors within each ecosystem.

The net ecosystem C balance is determined by C assimilation through photosynthesis, C loss through ecosystem respiration, and non-respiration losses such as soil DOC leaching^41^. Our results showed that almost all of the C influxes were stimulated by experimental N addition, whereas only DOC was stimulated by N addition as one of the C effluxes (Fig. 4). N addition significantly increased both ANPP and aboveground litterfall but had no impact on litter decomposition (Fig. 4). These results indicate that N deposition can stimulate C stocks in plant and litter C pools. However, N addition significantly increased the soil DOC content (Fig. 4), indicating an increased loss of C from soil C pools. Nevertheless, not all the increased DOC can be leached out, and the total DOC loss from global terrestrial soil, i.e., approximately 0.4 Pg C/yr^42^, is much smaller than the input from plant litter, which is approximately 60 Pg C/yr^43^. Given the relatively small amount of leached DOC, the soil C content is more likely to increase as a whole. Furthermore, given the insignificant response of soil respiration to N addition, our results suggest that terrestrial ecosystems might act as a C sink rather than a C source under further increases in N deposition as a whole. Meanwhile, our results also showed that C dynamics can show different response to N-only and multi-nutrient additions, indicating that combining other nutrients with N can bias the assessment of N deposition effects on terrestrial ecosystem C dynamics. Moreover, we also need to pay more attention to the negative effects of N deposition on ecosystem, as the altered C dynamics may result in changes in both ecosystem process and functioning, such as eutrophication as a result of the direct effect of N deposition and indirect effect of increased DOC leaching induced by N deposition.

Although the present meta-analysis provides a statistical evaluation of the response of the terrestrial ecosystem C cycle to N addition as a whole, some uncertainties still remain due to the inherent limitations of the studies’ methodologies and experimental manipulations. First, most of the experiments of N addition included in the present analysis were conducted via manual dispersal of particle N or sprays of N solution in the canopy, which fail to simulate natural N deposition given N retention in the canopy and its influence on the canopy process^44^. Second, the levels of N addition used in most of the experiments were much higher than the natural deposition rates as the worldwide deposition rate is typically <50 kg N/ha/yr^45^. Such experimental levels of N addition may therefore overestimate the effects of N deposition on terrestrial C dynamics under natural conditions given that higher N addition levels typically have stronger effects^30^. Third, current N-deposition rates at the northernmost high latitude sites (e.g., Canada and Siberia) are still very low and are not rising rapidly, while deposition rates at more southern locations, such as the Netherlands, are high and rising rapidly or stabilizing at high values^46^,^47^. As N-addition effects have been found to be weaker in areas with high N deposition than in areas with low N deposition^48^, pooling the results across entire ecosystems may overestimate N-deposition effects in southern areas and underestimate them in northern ones. Therefore, the evaluation of the effects of N addition may be biased to some extent. Moreover, as studies with N addition can also endure other environmental changes such as elevated CO_2_ and warming, estimations of the effects of N addition may be confounded by such environmental factors to some extent. Therefore, further studies that focus on the effects of multiple global change factors on C cycles better capture the complex interactive process for both theoretical and model development.

## Methods

### Data collection and extraction

Peer-reviewed journal articles evaluating the effects of N addition on C dynamics in terrestrial ecosystems were identified by searching the *Web of Science* and *Google Scholar* on 18 March 2015, with no restrictions on the publication year, and collected. The search terms used in online databases were “(nitrogen addition OR nitrogen enrichment OR nitrogen deposition OR nitrogen supply OR nitrogen fertilization OR nitrogen limit) AND (microbial biomass OR microbial respiration OR soil carbon OR soil respiration OR plant biomass OR plant carbon OR root biomass OR root carbon OR litterfall OR litter production OR litter decomposition OR litter decay OR litter carbon OR dissolved organic carbon)” and their equivalents in Chinese. To minimize publication bias, only primary studies that satisfied the following criteria were included in the analysis: (i) field experiments with N-addition treatments and a control were conducted in which at least one of the considered variables was evaluated; (ii) experimental and control plots were established within the same ecosystem and contrasted only with respect to the target variable; (iii) treatments using other fertilizers (e.g., P and K) in addition to N were excluded, as were treatments with N-addition gradients, i.e., different addition levels among different years for a specific case study (any comparison of a treatment and control was designated a “case study”) but not the entire study; (iv) the magnitude and experimental duration were clearly recorded, and measurements of the variables in the experimental and control groups were performed at the same spatial and temporal scales; (v) experiments whose durations were less than one growing season were excluded to avoid the influence of short-term noise; and (vi) the means, sample sizes, and standard deviations (SDs) or standard errors (SEs) of the chosen variables were directly provided or could be calculated from the studies.

Given the statistical assumption of independence among case studies in a meta-analysis^49^, the measurement from the most recent sampling was used if multiple measurements conducted at different times were reported within the same study. Within an individual study, the effects of different levels or/and chemical forms of N addition on C dynamics under treatment and control conditions were often compared, and thus, several effect sizes were usually obtained in a single primary study. However, these effect sizes may also be non-independent; however, including only one case study per study would reduce the available information and limit the analysis of moderators^50^. We therefore included the multiple cases, which we considered as independent case studies in the analyses, and then assessed the potential effects of including multiple case studies per study using a sensitivity analysis^50^,^51^. After extraction, a total of 198 articles were included in our analysis (Suppl. Table S2, S3). The database included C pools and fluxes from plants, plant litter, microorganisms, and soil. The plant C pools in the above- and below-ground parts were determined by assessing either plant biomass or C contents^52^. The litter C pool was described based on the litter C stock, and the soil C pool was determined from the soil C content and C storage, including soil total C, soil organic C (SOC), soil inorganic C (SIC), and DOC. The microbial C pool data were obtained from primary studies that directly reported microbial biomass C (MBC), in addition to all of the C flux data that could be directly extracted from studies, including information such as NPP, the average or cumulative litter mass loss, microbial respiration, and soil respiration in response to N addition.

### Moderator variables

The magnitude of the response of C dynamics to N addition may be affected by various environmental and forcing factors. Therefore, some of the variables were further categorized into the following groups, which are referred to as moderators: ecosystem type (forest, grassland, wetland, tundra, cropland, or desert); N chemical form [ammonium nitrate (NH_4_NO_3_), urea, ammonium (NH_4_), or nitrate (NO_3_)]; level of N addition (<50 kg N/ha/yr, 50−150 kg N/ha/yr, or >150 kg N/ha/yr)^26^; and plant functional types (PFT) according to the growth form (woody, herbaceous, or moss). However, as some of the moderators were continuous variables (MAT, MAP, latitude, experimental duration, soil depth, and N-addition level), we thus tested the linear and/or non-linear relationships between these moderator variables and the response of C dynamics to N addition. Where data were presented graphically in the selected primary studies, the figures were digitized to extract the numerical values using the free software Engauge Digitizer (Free Software Foundation, Inc., Boston, MA, USA). In addition, environmental factors such as MAT, MAP, and latitude were either entered into our database directly from the primary studies when available or extracted from the *WorldClim* database (http//: www.worldclim.org) using the studies’ location information.

### Effect size and meta-analysis

The data were analysed using a meta-analysis approach, and the effects of N addition on the terrestrial C pools and fluxes were estimated based on the natural log-transformed response ratio lnRR = ln(*X*_*e*_*/X*_*c*_), where *X*_*e*_ is the treatment mean and *X*_*c*_ is the control mean^49^. The variance associated with each value of lnRR was calculated from the SD of each parameter related to C dynamics^53^. If only the SE was available in the primary studies, we first converted it into the SD before calculating the variance^54^. Moreover, the effects of the moderators on the magnitude and direction of the responses of the C pools and fluxes to N addition were evaluated according to the available sample size. The heterogeneity within (Q_*within*_) and between (Q_*between*_) moderator levels was compared using mixed models to assess the significance of each categorical moderator^53^. The difference between two groups was considered insignificant if their 95% confidence intervals (CIs) overlapped^53^. The percentage change in response to N addition was calculated as the mean percentage of the change in the C content for a C pool or the change in the C influx/efflux for a C flux in relation to the control (%). Here, the equation (exp(lnRR) −1) ×100% was used, and the effects were considered insignificant at *P* < 0.05 if their 95% CIs overlapped zero. The average response ratio (lnRR) and its related 95% CI were calculated using the mixed model of the meta-analytical software MetaWin 2.1^55^.

### Sensitivity analysis and publication bias

Considering the possible non-independence of case studies selected from a specific primary study, we repeated the analysis using an averaged effect size per primary study, which was calculated as the mean effect size of all of the cases considered within that study using a mixed-effects model, similar to Ferreira *et al.*^50^. The analysis was then performed as described above. Moreover, we evaluated the publication bias in the overall database for each variable related to C dynamics included in this analysis with funnel plots, which are scatterplots of the effect sizes versus the sample sizes of individual studies, using MetaWin 2.1^55^. The funnel plots of all of the variables were symmetrical, which suggested an absence of publication bias.

## Additional Information

**How to cite this article**: Yue, K. *et al.* Stimulation of terrestrial ecosystem carbon storage by nitrogen addition: a meta-analysis. *Sci. Rep.* **6**, 19895; doi: 10.1038/srep19895 (2016).

## Supplementary Material

Supplementary Information

## Figures and Tables

**Table 1:** Regression analysis results of the response ratios (lnRR) of C pools and fluxes against N addition amount, duration, and environmental factors across all ecosystem groups.

Variable *X*	Addition amount					Duration					MAT					MAP					Latitude				
	*a*	*b*	*n*	*R*^*2*^	*P*	*a*	*b*	*n*	*R*^*2*^	*P*	*a*	*b*	*n*	*R*^*2*^	*P*	*a*	*b*	*n*	*R*^*2*^	*P*	*a*	*b*	*n*	*R*^*2*^	*P*
PAPC	0.155	0.000	54	0.027	0.235	0.262	−0.010	54	0.012	0.435	0.297	−0.007	54	0.030	0.207	0.344	0.000	54	0.060	0.074	0.238	0.000	54	0.000	0.924
PBPC	0.063	0.000	42	0.005	0.648	0.116	−0.003	42	0.001	0.885	0.038	0.007	42	0.016	0.426	0.211	0.000	42	0.034	0.239	0.280	−0.004	42	0.015	0.434
LC	0.179	0.000	17	0.007	0.749	0.119	0.015	17	0.073	0.295	0.215	0.001	17	0.000	0.996	0.359	0.000	17	0.056	0.359	0.048	0.004	17	0.034	0.479
STC_O-horizon_	0.056	−0.001	22	0.148	0.077	−0.318	0.017	22	0.160	0.065	0.048	−0.020	22	0.102	0.148	−0.061	−0.001	22	0.003	0.812	−0.572	0.010	22	0.129	0.101
STC_Mineral_	0.090	−0.001	165	0.002	0.555	0.016	**0.009**	165	0.071	0.001	0.081	0.000	165	0.000	0.906	0.068	0.000	165	0.001	0.729	0.016	0.002	165	0.008	0.258
SOC	−0.064	0.000	63	0.006	0.342	−0.033	0.004	63	0.001	0.779	0.115	−0.010	63	0.019	0.281	0.067	−0.001	63	0.009	0.468	−0.324	0.009	63	0.024	0.221
DOC	0.131	0.001	87	0.000	0.931	0.153	−0.005	87	0.002	0.649	0.114	0.002	87	0.003	0.598	0.107	0.001	87	0.003	0.625	−0.041	0.005	87	0.016	0.236
MBC_O-horizon_	−0.863		9	0.617	0.012	0.041	**−0.059**	9	0.608	0.013	−0.617	0.025	9	0.255	0.165	−0.704	0.000	9	0.142	0.318	−0.024	−0.010	9	0.242	0.178
MBC_Mineral_	0.024	0.000	176	0.005	0.344	0.149		172	0.085	0.000	0.089	−0.009	176	0.018	0.073	0.201		176	0.045	0.005	−0.308	**0.008**	176	0.038	0.009
ANPP	0.382	0.000	21	0.006	0.745	0.413	−0.002	21	0.000	0.945	0.411	0.000	21	0.001	0.907	0.463	−0.001	21	0.017	0.574	0.324	0.002	21	0.013	0.618
Litterfall	0.152	−0.001	37	0.001	0.885	0.179	−0.007	37	0.023	0.374	0.199	−0.005	37	0.041	0.230	0.193	−0.001	37	0.029	0.313	0.028	0.003	37	0.038	0.250
LDR	−0.076	**0.001**	89	0.070	0.012	0.024	0.000	89	0.024	0.148	0.026	−0.003	89	0.021	0.174	−0.082	**0.001**	89	0.078	0.008	0.003	0.000	89	0.000	0.915
SR	−0.046	0.000	126	0.021	0.108	0.012	−0.003	134	0.001	0.706	−0.002	0.000	134	0.000	0.916	−0.009	0.001	134	0.001	0.713	0.067	−0.002	134	0.008	0.310
MR	0.106	0.000	46	0.057	0.109	−0.014	0.017	46	0.036	0.207	0.057	−0.001	46	0.002	0.770	0.064	−0.001	46	0.002	0.755	0.024	0.001	46	0.001	0.821

PAPC: plant aboveground part C; PBPC: plant belowground part C; LC: Litter C; STC_O-horizon_: soil total C of O-horizon; STC_Mineral_: soil total C of mineral layer; SOC: soil organic C; DOC: soil dissolved organic C; MBC_O-horizon_:microbial biomass C in O-horizon; MBC_Mineral_: microbial biomass C in mineral layer; ANPP: aboveground net primary productivity; LDR: litter decomposition rate; SR: soil respiration; MR: microbial respiration; MAT: mean annual temperature; MAP: mean annual precipitation. The regression analysis was based on lnRR = *a* + *bX*, where *X* is the independent variable in the first line, *a* is the intercept, and *b* is the regression coefficient. *n* is the sample size, *R^2^* is the determinant coefficient and *P* is probability of significance of the regression relationship. *b* values in bold are statistically significant.
