# Supplementary material for: Stimulation of terrestrial ecosystem carbon storage by nitrogen addition: a meta-analysis
Source: Sci Rep. 2016 Jan 27;6:19895. doi: 10.1038/srep19895 (PMC4728605; doi:10.1038/srep19895)
Supplement: Supplementary Information [file srep19895-s1.pdf]

SUPPLEMENTARY INFORMATION TO

Stimulation of terrestrial ecosystem carbon storage by nitrogen addition: a meta-analysis

Kai Yue[1 †], Yan Peng[1 †], Changhui Peng[2, 3], Wanqin Yang[1], Xin Peng[4] & Fuzhong Wu[1*]

[†]These authors contributed equally to the present work.

[*]Corresponding author

Supplementary Table S1 Summary table for subgroup analysis considering a mean effect size per primary study. Sample size (n), mean percentage change in carbon pool or fluxes, 95% confidence interval (CI), test for heterogeneity of all effect size (Q_{total}), and degrees of freedom (df) and *P*-value are shown. Levels with different letter differ significantly.

	Subgroups	n	Mean changes in carbon (%)	%95 CI
Carbon pool				
	Plant aboveground part C	28	37.82	[11.89, 69.76]
		Q_{total} = 14.07, df = 27, *P* = 0.9805		
	Plant belowground part C	20	19.01	[3.08, 37.36]
		Q_{total} = 30.27, df = 19, *P* = 0.0484		
	Plant litter C	8	17.08	[2.183, 34.15]
		Q_{total} = 10.81, df = 7, *P* = 0.2122		
	Soil total C	39	15.14	[4.90, 26.38]
		Q_{total} = 53.16, df = 38, *P* = 0.0521		
	Soil organic	18	2.58	[-9.14, 15.82]
		Q_{total} = 9.28, df = 17, *P* = 0.9310		
	Soil dissolved organic C	17	11.78	[0.20, 24.69]
		Q_{total} = 32.52, df = 18, *P* = 0.0086		
	Microbial biomass C	49	1.47	[-8.32, 12.31]
		Q_{total} = 35.97, df = 48, *P* = 0.8996		
Carbon fluxes				
	ANPP	10	45.93	[32.11, 61.19]
		Q_{total} = 8.36, df = 9, *P* = 0.4980		
	BNPP	2	5.39	[-87.67, 800.61]
		Q_{total} = 1.00, df = 1, *P* = 0.3173		
	NPP	3	38.76	[16.6, 65.10]
		Q_{total} = 0.13, df = 2, *P* = 0.9376		

	Litterfall	12	8.47	[0.42, 17.17]
		Q_{total} =10.06, df = 11, *P* = 0.5249		
	Litter decomposition	13	-0.17	[-16.23, 18.22]
		Q_{total} = 11.03, df = 12, *P* = 0.5267		
	Soil respiration	52	-1.20	[-7.94, 6.02]
		Q_{total} = 75.34, df = 51, *P* = 0.0149		
	Microbial respiration	12	-2.77	[-16.22, 12.85]
		Q_{total} = 8.22, df = 11, *P* = 0.6932		
	ER	4	32.29	[-28.92, 146.21]
		Q_{total} = 3.47, df = 3, *P* = 0.3248		
	GEP	2	66.50	[-85.47, 1807.92]
		Q_{total} = 1, df = 1, *P* = 0.3173		
	GPP	2	2.95	[-46.4, 97.72]
		Q_{total} = 1, df = 1, *P* = 0.3173		

Supplementary Table S2. Data matrix used in this meta-analysis. Study reference, effect size (lnR), variance (VlnR), and moderator variables are given.

Reference	Site location	Response variable	Soil depth (cm)	Ecosystem	Latitude (°)	MAT (℃)	MAP (mm)	N form	N addition level (kg/ha/yr)	Duration (yr, month for litter decomposition)	lnRR	Variance of lnRR	PFT-1	PFT-2
Mack et al. 2004	Toolik Lake, Alaska	litter C		tundra	68.38 N	-6.1	180	NH_4NO_3	100	20	0.3844	0.0718		
Mack et al. 2004	Toolik Lake, Alaska	ANPP		tundra	68.38 N	-6.1	180	NH_4NO_3	100	20	0.5643	0.0431		vascular plant
Mack et al. 2004	Toolik Lake, Alaska	soil total C	O-horizon	tundra	68.38 N	-6.1	180	NH_4NO_3	100	20	-0.1555	0.1221		
Mack et al. 2004	Toolik Lake, Alaska	soil total C	0-20	tundra	68.38 N	-6.1	180	NH_4NO_3	100	20	-0.5407	0.1197		
Magill et al. 2004	Harvard forest, UAS	DOC	0-60	forest	42.3 N	6.5	1120	NH_4NO_3	50	15	0.1625	0.018		
Magill et al. 2004	Harvard forest, UAS	DOC	0-60	forest	42.3 N	6.5	1120	NH_4NO_3	150	15	0.1534	0.0127		
Magill et al. 2004	Harvard forest, UAS	DOC	0-60	forest	42.3 N	6.5	1120	NH_4NO_3	50	15	-0.2185	0.0155		
Magill et al. 2004	Harvard forest, UAS	DOC	0-60	forest	42.3 N	6.5	1120	NH_4NO_3	150	15	-0.3201	0.0121		
Magill et al. 2004	Harvard forest, UAS	litterfall		forest	42.3 N	6.5	1120	NH_4NO_3	50	15	0.4014	0.1112		
Magill et al. 2004	Harvard forest, UAS	litterfall		forest	42.3 N	6.5	1120	NH_4NO_3	150	15	-0.4923	0.2201		
Magill et al. 2004	Harvard forest, UAS	litterfall		forest	42.3 N	6.5	1120	NH_4NO_3	50	15	-0.0538	0.0556		
Magill et al. 2004	Harvard forest, UAS	litterfall		forest	42.3 N	6.5	1120	NH_4NO_3	150	15	-0.1327	0.0477		
Mo et al. 2008	Guangdong, China	soil respiration		forest	23.1 N	21	1927	NH_4NO_3	50	1	0.0528	0.0039		
Mo et al. 2008	Guangdong, China	soil respiration		forest	23.1 N	21	1927	NH_4NO_3	100	1	-0.0815	0.0194		
Mo et al. 2008	Guangdong, China	soil respiration		forest	23.1 N	21	1927	NH_4NO_3	150	1	-0.1562	0.0028		
Mo et al. 2008	Guangdong, China	MBC	0-10	forest	23.1 N	21	1927	NH_4NO_3	50	1	-0.0671	0.0034		
Mo et al. 2008	Guangdong, China	MBC	0-10	forest	23.1 N	21	1927	NH_4NO_3	100	1	-0.2007	0.0026		
Mo et al. 2008	Guangdong, China	MBC	0-10	forest	23.1 N	21	1927	NH_4NO_3	150	1	-0.292	0.0137		
Mo et al. 2008	Guangdong, China	DOC	0-10	forest	23.1 N	21	1927	NH_4NO_3	50	1	-0.0822	0.006		
Mo et al. 2008	Guangdong, China	DOC	0-10	forest	23.1 N	21	1927	NH_4NO_3	100	1	0.1236	0.0049		

Mo et al. 2008	Guangdong, China	DOC	0-10	forest	23.1 N	21	1927	NH_4NO_3	150	1	0.2126	0.0032	
Bennett and Adams 2001	Western Australia	litter C		grassland	22.17 S	33	350	urea	50	2	0.608	0.0299	
Bennett and Adams 2001	Western Australia	plant aboveground part C		grassland	22.17 S	33	350	urea	50	2	0.1488	0.011	total
Campo and Vazquez-Yanes 2004	Yucatan, Mexico	litterfall		forest	21.06 N	25.8	760	urea	220	3	0.0859	0.0034	
Campo and Vazquez-Yanes 2004	Yucatan, Mexico	litterfall		forest	21.06 N	25.8	760	urea	220	3	0.0635	0.0055	
Campo and Vazquez-Yanes 2004	Yucatan, Mexico	litter C		forest	21.06 N	25.8	760	urea	220	3	0	0.0176	
Campo and Vazquez-Yanes 2004	Yucatan, Mexico	litter C		forest	21.06 N	25.8	760	urea	220	3	0.0443	0.0179	
Carpenter et al., 1990	Piceance Basin, Colorado	ANPP		grassland	39.51 N	11.8	282	NH_4NO_3	100	3	0.8854	0.0129	herbaceous
Carpenter et al., 1990	Piceance Basin, Colorado	ANPP		grassland	39.51 N	11.8	282	NH_4NO_3	100	3	-0.8938	0.2043	woody
D'Antonio and Mack. 2006	Hawaii Volcanoes National Park	plant belowground part C		grassland	19.6 N	20.5	1500	NH_4	100	2	0.6433	0.0469	total
Dukes et al. 2005	JRGCE, Woodside, California	NPP		grassland	37.24 N	13.6	655	NO_3	20	5	0.2869	0.0224	total
Dukes et al. 2005	JRGCE, Woodside, California	plant aboveground part C		grassland	37.24 N	13.6	655	NO_3	20	5	0.2902	0.0103	total
Dukes et al. 2005	JRGCE, Woodside, California	plant belowground part C		grassland	37.24 N	13.6	655	NO_3	20	5	0.1473	0.033	total
Davis et al. 2004	Craigieburn Range, New Zealand	litterfall		forest	43.15 S	8	1447	urea	400	2	0.0817	0.0073	
Davis et al. 2004	Craigieburn Range, New Zealand	litterfall		forest	43.15 S	8	1447	urea	400	2	0.035	0.0027	
Bobbink 1991	Limburg, Netherlands	plant aboveground part C		grassland	50.51 N	10	800	NH_4NO_3	100	3	0.7195	0.0224	total
Bobbink 1991	Limburg, Netherlands	plant aboveground part C		grassland	50.51 N	10	800	NH_4NO_3	100	3	0.3827	0.058	total
Bobbink 1991	Limburg, Netherlands	plant aboveground part C		grassland	50.51 N	10	800	NH_4NO_3	100	3	-0.2231	0.0153	total
Bobbink 1991	Limburg, Netherlands	plant aboveground part C		grassland	50.51 N	10	800	NH_4NO_3	100	3	-0.3623	0.0244	total
Burton et al. 2004	Michigan, USA	soil respiration		forest	46.52 N	4.8	821	NO_3	30	8	-0.0535	0.0192	
Burton et al. 2004	Michigan, USA	soil respiration		forest	45.33 N	6.1	828	NO_3	30	8	-0.0987	0.0209	
Burton et al. 2004	Michigan, USA	soil respiration		forest	44.23 N	6.9	856	NO_3	30	8	-0.3342	0.0123	

Burton et al. 2004	Michigan, USA	soil respiration		forest	43.40 N	7.6	793	NO_3	30	8	-0.182	0.0099	
Burton et al. 2004	Michigan, USA	plant belowground part C		forest	46.52 N	4.8	821	NO_3	30	8	-0.1352	0.008	total
Burton et al. 2004	Michigan, USA	plant belowground part C		forest	45.33 N	6.1	828	NO_3	30	8	0.0784	0.0014	total
Burton et al. 2004	Michigan, USA	plant belowground part C		forest	44.23 N	6.9	856	NO_3	30	8	0.121	0.0076	total
Burton et al. 2004	Michigan, USA	plant belowground part C		forest	43.40 N	7.6	793	NO_3	30	8	0.0087	0.0104	total
Iversen and Norby 2008	Eastern Tennessee, UAS	ANPP		forest	35.54 N	13.9	1371	urea	200	1	0.3283	0.0003	total
Cleveland and Townsend 2006	Costa Rica	soil respiration		forest	8.43 N	22.3	5000	NH_4NO_3	150	2	0.2917	0.0158	
Baer and Blair 2008	Manhattan, Kansas, UAS	ANPP		grassland	39.05 N	12.7	1268	NH_4NO_3	50	8	0.3956	0.0088	total
Baer and Blair 2008	Manhattan, Kansas, UAS	plant belowground part C		grassland	39.05 N	12.7	1268	NH_4NO_3	50	8	-0.003	0.0202	total
Baer and Blair 2008	Manhattan, Kansas, UAS	soil total C	0-10	grassland	39.05 N	12.7	1268	NH_4NO_3	50	8	0.0434	0.0008	
Baer and Blair 2008	Manhattan, Kansas, UAS	MBC	0-10	grassland	39.05 N	12.7	1268	NH_4NO_3	50	8	-0.0052	0.0111	
Jourdan et al. 2008	Sao Paulo State, Brazil	plant belowground part C		forest	23.02 N	19	1360	NH_4	120	1	1.5404	0.0506	total
Persson and Ahlstron 2001	Hacksvik	plant belowground part C		forest	59.51 N	6.91	571	NH_4NO_3	150	4	0.562	0.5609	total
Persson and Ahlstron 2001	Hacksvik	plant belowground part C		forest	59.51 N	6.91	571	NH_4NO_3	225	4	0.9261	0.6051	total
Persson and Ahlstron 2001	Nissafors	plant belowground part C		forest	59.51 N	6.91	571	NH_4NO_3	150	4	0.0438	0.6784	total
Persson and Ahlstron 2001	Strasan	plant belowground part C		forest	59.51 N	6.91	571	NH_4NO_3	150	4	-0.3726	0.5668	total
Persson and Ahlstron 2001	Strasan	plant belowground part C		forest	59.51 N	6.91	571	NH_4NO_3	225	4	-0.3774	0.6957	total
Persson and Ahlstron 2001	Varnamo	plant belowground part C		forest	59.51 N	6.91	571	NH_4NO_3	150	4	0.712	0.6293	total
Persson and Ahlstron 2001	Varnamo	plant belowground part C		forest	59.51 N	6.91	571	urea	150	4	0.7896	0.5624	total
Persson and Ahlstron 2001	Vra	plant belowground part C		forest	59.51 N	6.91	571	NH_4NO_3	150	4	-0.7635	0.6842	total
Persson and Ahlstron 2001	Vra	plant belowground part C		forest	59.51 N	6.91	571	urea	150	4	-0.5076	0.6198	total
Dijkstra et al. 2005	Minnesota, USA	MBC	0-20	grassland	44.72 N	5.5	660	NH_4NO_3	40	4	-0.0617	0.0016	
Dijkstra et al. 2005	Minnesota, USA	soil total C	0-20	grassland	44.72 N	5.5	660	NH_4NO_3	40	4	-0.0168	0.0023	
Dijkstra et al. 2005	Minnesota, USA	soil total C	0-20	grassland	44.72 N	5.5	660	NH_4NO_3	40	4	0.08	0.0032	
Schaeffer et al. 2003	Nye County, Nevada	soil respiration		desert	36.49 N	26	140	NO_3		2	-0.6593	0.1084	
Schaeffer et al. 2003	Nye County, Nevada	soil respiration		desert	36.49 N	26	140	NO_3		2	-0.5285	0.2611	

Schaeffer et al. 2003	Nye County, Nevada	soil respiration		desert	36.49 N	26	140	NO_3		2	0.3382	0.2141
Schaeffer et al. 2003	Nye County, Nevada	soil respiration		desert	36.49 N	26	140	NO_3		2	-0.409	0.2541
Schmidt et al. 2004	Niwot Ridge, UAS	MBC	0-10	tundra	40.03 N	26	140	NH_4NO_3	100	6	-0.3217	0.024
Compton et al. 2004	Harvard forest, UAS	MBC	0-20	forest	42.3 N	6.5	1120	NH_4NO_3	50	15	-0.4212	0.1977
Compton et al. 2004	Harvard forest, UAS	MBC	0-20	forest	42.3 N	6.5	1120	NH_4NO_3	150	15	-0.6252	0.1945
Compton et al. 2004	Harvard forest, UAS	MBC	0-20	forest	42.3 N	6.5	1120	NH_4NO_3	50	15	-0.0377	0.0695
Compton et al. 2004	Harvard forest, UAS	MBC	0-20	forest	42.3 N	6.5	1120	NH_4NO_3	150	15	-2.2842	4.0022
Waldrop et al. 2004 a	Lower Michigan, UAS	MBC	0-10	forest	44.22 N	7.2	810	NO_3	30	1	0.2606	0.0089
Waldrop et al. 2004 a	Lower Michigan, UAS	MBC	0-10	forest	44.22 N	7.2	810	NO_3	30	1	0.0513	0.0053
Waldrop et al. 2004 a	Lower Michigan, UAS	MBC	0-10	forest	44.22 N	7.2	810	NO_3	30	1	-0.0517	0.0247
Waldrop et al. 2004 a	Lower Michigan, UAS	MBC	0-10	forest	44.22 N	7.2	810	NO_3	80	1	0.2476	0.0063
Waldrop et al. 2004 a	Lower Michigan, UAS	MBC	0-10	forest	44.22 N	7.2	810	NO_3	80	1	0.1001	0.005
Waldrop et al. 2004 a	Lower Michigan, UAS	MBC	0-10	forest	44.22 N	7.2	810	NO_3	80	1	-0.3649	0.0131
Waldrop et al. 2004 a	Lower Michigan, UAS	microbial respiration	0-10	forest	44.22 N	7.2	810	NO_3	30	1	0.1391	0.0183
Waldrop et al. 2004 a	Lower Michigan, UAS	microbial respiration	0-10	forest	44.22 N	7.2	810	NO_3	30	1	0.1136	0.0121
Waldrop et al. 2004 a	Lower Michigan, UAS	microbial respiration	0-10	forest	44.22 N	7.2	810	NO_3	30	1	0	0.0313
Waldrop et al. 2004 a	Lower Michigan, UAS	microbial respiration	0-10	forest	44.22 N	7.2	810	NO_3	80	1	0.4855	0.0207
Waldrop et al. 2004 a	Lower Michigan, UAS	microbial respiration	0-10	forest	44.22 N	7.2	810	NO_3	80	1	0.192	0.0073
Waldrop et al. 2004 a	Lower Michigan, UAS	microbial respiration	0-10	forest	44.22 N	7.2	810	NO_3	80	1	-0.168	0.0184
Waldrop et al. 2004 a	Lower Michigan, UAS	soil total C	0-10	forest	44.22 N	7.2	810	NO_3	30	1	0.2181	0.0963

Waldrop et al. 2004 a	Lower Michigan, UAS	soil total C	0-10	forest	44.22 N	7.2	810	NO_3	30	1	-0.0959	0.052
Waldrop et al. 2004 a	Lower Michigan, UAS	soil total C	0-10	forest	44.22 N	7.2	810	NO_3	30	1	-0.0068	0.0782
Waldrop et al. 2004 a	Lower Michigan, UAS	soil total C	0-10	forest	44.22 N	7.2	810	NO_3	80	1	0.2908	0.0465
Waldrop et al. 2004 a	Lower Michigan, UAS	soil total C	0-10	forest	44.22 N	7.2	810	NO_3	80	1	0.1385	0.0402
Waldrop et al. 2004 a	Lower Michigan, UAS	soil total C	0-10	forest	44.22 N	7.2	810	NO_3	80	1	-0.3197	0.0239
Waldrop et al. 2004 b	Lower Michigan, UAS	soil total C	0-20	forest	44.22 N	7.2	810	NO_3	80	3	0.1178	0.0254
Waldrop et al. 2004 b	Lower Michigan, UAS	soil total C	0-20	forest	44.22 N	7.2	810	NO_3	80	3	0.1309	0.0584
Waldrop et al. 2004 b	Lower Michigan, UAS	soil total C	0-20	forest	44.22 N	7.2	810	NO_3	80	3	-0.2386	0.0342
Wallenstein et al. 2006	Maine, USA	MBC	0-10	forest	44.52 N	7.5	1170	NH_4	25.2	15	-0.0772	0.0068
Wallenstein et al. 2006	Maine, USA	MBC	0-10	forest	44.52 N	7.5	1170	NH_4	25.2	15	-0.1661	0.0988
Wallenstein et al. 2006	Harvard Forest, USA	MBC	0-10	forest	42.3 N	6	1100	NH_4NO_3	50	15	-0.162	0.0244
Wallenstein et al. 2006	Harvard Forest, USA	MBC	0-10	forest	42.3 N	6	1100	NH_4NO_3	150	15	-0.9328	0.0116
Wallenstein et al. 2006	Harvard Forest, USA	MBC	0-10	forest	42.3 N	6	1100	NH_4NO_3	50	15	0.2329	0.0098
Wallenstein et al. 2006	Harvard Forest, USA	MBC	0-10	forest	42.3 N	6	1100	NH_4NO_3	150	15	-0.3054	0.0143
Wallenstein et al. 2006	Mt. Ascutney, UAS	MBC	O-horizon	forest	43.26 N	6	1100	NH_4	15.7	15	-1.1407	0.1127
Wallenstein et al. 2006	Mt. Ascutney, UAS	MBC	O-horizon	forest	43.26 N	6	1100	NH_4	31.4	15	-1.1799	0.0229
Wang et al. 2008b	Hunan, China	soil total C	0-20	forest	26.31 N	15.8	1300	urea	100	1	-0.0447	0.0005
Wang et al. 2008b	Hunan, China	soil total C	0-20	forest	26.31 N	15.8	1300	urea	200	1	-0.0754	0.0026
Wang et al. 2008b	Hunan, China	DOC	0-20	forest	26.31 N	15.8	1300	urea	100	1	0.0347	0.0045
Wang et al. 2008b	Hunan, China	DOC	0-20	forest	26.31 N	15.8	1300	urea	200	1	0.0682	0.009
Wang et al. 2008b	Hunan, China	MBC	0-20	forest	26.31 N	15.8	1300	urea	100	1	-0.1847	0.0074
Wang et al. 2008b	Hunan, China	MBC	0-20	forest	26.31 N	15.8	1300	urea	200	1	-0.3483	0.0043
Chen et al. 2002	Queensland, Australia	soil total C	0-10	forest	26.28 S	21.4	993	NH_4NO_3	300	5	0.0022	0.0225
Chen et al. 2002	Queensland, Australia	soil total C	0-10	forest	26.28 S	21.4	993	NH_4NO_3	600	5	0.0844	0.0116
Chen et al. 2002	Queensland, Australia	soil total C	10-20	forest	26.28 S	21.4	993	NH_4NO_3	300	5	0.0953	0.0265
Chen et al. 2002	Queensland, Australia	soil total C	10-20	forest	26.28 S	21.4	993	NH_4NO_3	600	5	0.3015	0.0481

Chen et al. 2002	Queensland, Australia	MBC	0-10	forest	26.28 S	21.4	993	NH_4NO_3	300	5	-0.2522	0.0488
Chen et al. 2002	Queensland, Australia	MBC	0-10	forest	26.28 S	21.4	993	NH_4NO_3	600	5	-0.2432	0.0507
Chen et al. 2002	Queensland, Australia	MBC	10-20	forest	26.28 S	21.4	993	NH_4NO_3	300	5	0.0024	0.0866
Chen et al. 2002	Queensland, Australia	MBC	10-20	forest	26.28 S	21.4	993	NH_4NO_3	600	5	0.3571	0.0801
Chen et al. 2002	Queensland, Australia	microbial respiration	0-10	forest	26.28 S	21.4	993	NH_4NO_3	300	5	-0.2017	0.0213
Chen et al. 2002	Queensland, Australia	microbial respiration	0-10	forest	26.28 S	21.4	993	NH_4NO_3	600	5	-0.1446	0.0294
Chen et al. 2002	Queensland, Australia	microbial respiration	10-20	forest	26.28 S	21.4	993	NH_4NO_3	300	5	-0.3102	0.0246
Chen et al. 2002	Queensland, Australia	microbial respiration	10-20	forest	26.28 S	21.4	993	NH_4NO_3	600	5	0.1252	0.0198
Brenner et al. 2005	Alaska, UAS	soil total C	0-20	forest	64.45 N	-3.7	269	NH_4NO_3	100	5	0.0089	0.009
Brenner et al. 2005	Alaska, UAS	soil total C	0-20	forest	64.45 N	-3.7	269	NH_4NO_3	100	5	-0.1338	0.0401
Brenner et al. 2005	Alaska, UAS	soil total C	20-30	forest	64.45 N	-3.7	269	NH_4NO_3	100	5	0.6737	0.193
Brenner et al. 2005	Alaska, UAS	soil total C	20-30	forest	64.45 N	-3.7	269	NH_4NO_3	100	5	0.0325	0.3506
Brenner et al. 2005	Alaska, UAS	MBC	0-20	forest	64.45 N	-3.7	269	NH_4NO_3	100	5	-0.1529	0.6739
Brenner et al. 2005	Alaska, UAS	MBC	0-20	forest	64.45 N	-3.7	269	NH_4NO_3	100	5	-0.062	0.0364
Torn et al. 2005	Thurston, Hawaii, USA	soil total C	O-horizon	forest	22.05 N	16	2500		100	12	0.1509	0.0242
Torn et al. 2005	Thurston, Hawaii, USA	soil total C	0-10	forest	22.05 N	16	2500		100	12	0.0526	0.0288
Torn et al. 2005	Kokee, Kauai, USA	soil total C	O-horizon	forest	22.05 N	16	2500		100	6	0.0992	0.0931
Torn et al. 2005	Kokee, Kauai, USA	soil total C	0-10	forest	22.05 N	16	2500		100	6	0.2428	0.2042
Torn et al. 2005	Thurston, Hawaii, USA	MBC	O-horizon	forest	22.05 N	16	2500		100	12	-0.0502	0.0481
Torn et al. 2005	Kokee, Kauai, USA	MBC	0-10	forest	22.05 N	16	2500		100	6	-0.5596	0.194
Barnard et al. 2006	Jasper Ridge Biological Preserve, California, USA	MBC		grassland	37.24 N	13.6	655	NO_3	70	15	0.0339	0.0047

Nohrstedt 1989	Kroksbo, Sweden	soil respiration		forest	60.3 N	8	539	NH_4NO_3	150	11	0	0.0046		
Nohrstedt 1989	Kroksbo, Sweden	soil respiration		forest	60.3 N	8	539	NH_4NO_3	600	11	-0.2683	0.0063		
Nohrstedt 1989	Kroksbo, Sweden	MBC		forest	60.3 N	8	539	NH_4NO_3	150	11	-0.0431	0.0036		
Nohrstedt 1989	Kroksbo, Sweden	MBC		forest	60.3 N	8	539	NH_4NO_3	600	11	-0.2263	0.0044		
Nohrstedt 1989	Kroksbo, Sweden	soil total C		forest	60.3 N	8	539	NH_4NO_3	150	11	0.1651	0.0131		
Nohrstedt 1989	Kroksbo, Sweden	soil total C		forest	60.3 N	8	539	NH_4NO_3	600	11	0.2485	0.0122		
Nohrstedt 1989	Kroksbo, Sweden	soil respiration		forest	60.3 N	8	539	urea	150	11	-0.0836	0.0051		
Nohrstedt 1989	Kroksbo, Sweden	soil respiration		forest	60.3 N	8	539	urea	600	11	-0.1252	0.0053		
Nohrstedt 1989	Kroksbo, Sweden	MBC		forest	60.3 N	8	539	urea	150	11	-0.1549	0.004		
Nohrstedt 1989	Kroksbo, Sweden	MBC		forest	60.3 N	8	539	urea	600	11	-0.2751	0.0047		
Nohrstedt 1989	Kroksbo, Sweden	soil total C		forest	60.3 N	8	539	urea	150	11	0.0253	0.0148		
Nohrstedt 1989	Kroksbo, Sweden	soil total C		forest	60.3 N	8	539	urea	600	11	0.3795	0.0112		
Nohrstedt 1989	Nissators, Sweden	soil respiration		forest	50.24 N	8.5	520	NH_4NO_3	150	9	0.007	0.0095		
Nohrstedt 1989	Nissators, Sweden	MBC		forest	50.24 N	8.5	520	NH_4NO_3	150	9	-0.091	0.011		
Nohrstedt 1989	Nissators, Sweden	soil total C		forest	50.24 N	8.5	520	NH_4NO_3	150	9	0.2231	0.0256		
Thirukkumaran & Parkinson 2002	Watershed, Alberta, Canada	soil total C	O-horizon	forest	52.2 N	1.9	660	NH_4NO_3	188	2	0.1036	0.0282		
Thirukkumaran & Parkinson 2002	Watershed, Alberta, Canada	soil total C	O-horizon	forest	52.2 N	1.9	660	urea	188	2	0.0878	0.0071		
Thirukkumaran & Parkinson 2002	Watershed, Alberta, Canada	microbial respiration	O-horizon	forest	52.2 N	1.9	660	NH_4NO_3	188	2	0.0195	0.0133		
Thirukkumaran & Parkinson 2002	Watershed, Alberta, Canada	microbial respiration	O-horizon	forest	52.2 N	1.9	660	urea	188	2	0.0078	0.0037		
Thirukkumaran & Parkinson 2002	Watershed, Alberta, Canada	litter decomposition rate	O-horizon	forest	52.2 N	1.9	660	NH_4NO_3	188	2	0.0263	0.0072	*Pinus*	woody

Thirukkumaran & Parkinson 2002	Watershed, Alberta, Canada	litter decomposition rate	O-horizon	forest	52.2 N	1.9	660	urea	188	2	0.1448	0.0086	*Pinus*	woody
Tripathi et al. 2008	Uttar Pradesh, India	MBC	0-10	forest	24.55 N	27	1220	urea	150	6	0.348	0.0032		
Tripathi et al. 2008	Uttar Pradesh, India	MBC	0-10	ecotone	24.55 N	27	1220	urea	150	6	0.3447	0.0056		
Tripathi et al. 2008	Uttar Pradesh, India	MBC	0-10	grassland	24.55 N	27	1220	urea	150	6	0.3429	0.0038		
Tripathi et al. 2008	Uttar Pradesh, India	soil total C	0-10	forest	24.55 N	27	1220	urea	150	6	0.2578	0.0045		
Tripathi et al. 2008	Uttar Pradesh, India	soil total C	0-10	ecotone	24.55 N	27	1220	urea	150	6	0.3085	0.0036		
Tripathi et al. 2008	Uttar Pradesh, India	soil total C	0-10	grassland	24.55 N	27	1220	urea	150	6	0.3008	0.0043		
Priess and Folster 2001	La Sabanita, San Francisco	MBC	O-horizon	forest	5 N	20.6	1700	NO_3	300	1	0.056	0.0133		
Priess and Folster 2001	La Sabanita, San Francisco	MBC	O-horizon	forest	5 N	20.6	1700	NO_3	300	1	-0.0106	0.0163		
Priess and Folster 2001	La Sabanita, San Francisco	MBC	O-horizon	forest	5 N	20.6	1700	NO_3	300	1	-0.003	0.0344		
Priess and Folster 2001	La Sabanita, San Francisco	MBC	0-10	forest	5 N	20.6	1700	NO_3	300	1	0.3242	0.0115		
Priess and Folster 2001	La Sabanita, San Francisco	MBC	0-10	forest	5 N	20.6	1700	NO_3	300	1	0.0661	0.028		
Priess and Folster 2001	La Sabanita, San Francisco	MBC	0-10	forest	5 N	20.6	1700	NO_3	300	1	0.206	0.0258		
Priess and Folster 2001	La Sabanita, San Francisco	microbial respiration	O-horizon	forest	5 N	20.6	1700	NO_3	300	1	-0.1335	0.0699		
Priess and Folster 2001	La Sabanita, San Francisco	microbial respiration	O-horizon	forest	5 N	20.6	1700	NO_3	300	1	-0.0513	0.0134		
Priess and Folster 2001	La Sabanita, San Francisco	microbial respiration	O-horizon	forest	5 N	20.6	1700	NO_3	300	1	0.0541	0.0028		
Priess and Folster 2001	La Sabanita, San Francisco	microbial respiration	0-10	forest	5 N	20.6	1700	NO_3	300	1	0.1369	0.0311		
Priess and Folster 2001	La Sabanita, San Francisco	microbial respiration	0-10	forest	5 N	20.6	1700	NO_3	300	1	0	0.0391		
Priess and Folster 2001	La Sabanita, San Francisco	microbial	0-10	forest	5 N	20.6	1700	NO_3	300	1	-0.2513	0.0117		

		respiration											
Priess and Folster 2001	La Sabanita, San Francisco	soil respiration		forest	5 N	20.6	1700	NO_3	300	1	0.2015	0.0293	
Priess and Folster 2001	La Sabanita, San Francisco	soil respiration		forest	5 N	20.6	1700	NO_3	300	1	-0.226	0.0337	
Priess and Folster 2001	La Sabanita, San Francisco	soil respiration		forest	5 N	20.6	1700	NO_3	300	1	-0.0521	0.0478	
Gundale et al. 2014	Vindeln, Sweden	plant aboveground part C		forest	64.14 N	0.9	498	NH_4NO_3	12.5	14	0.0274	0.0126	total
Gundale et al. 2014	Vindeln, Sweden	plant aboveground part C		forest	64.14 N	0.9	498	NH_4NO_3	50	14	0.0328	0.0125	total
West et al. 2006	Minnesota, USA	microbial respiration	0-20	grassland	45 N	5.7	660	NH_4NO_3	40	6	0.0695	0.0097	
West et al. 2006	Minnesota, USA	microbial respiration	0-20	grassland	45 N	5.7	660	NH_4NO_3	40	6	0.0105	0.0048	
West et al. 2006	Minnesota, USA	microbial respiration	0-20	grassland	45 N	5.7	660	NH_4NO_3	40	6	0.0313	0.0106	
West et al. 2006	Minnesota, USA	microbial respiration	0-20	grassland	45 N	5.7	660	NH_4NO_3	40	6	0.1721	0.0092	
Nowinsk et al. 2009	East of Los Angeles, USA	microbial respiration	O-horizon	forest	34.03 N	10.6	90	NH_4NO_3	50	10	0.1666	0.0092	
Nowinsk et al. 2009	East of Los Angeles, USA	microbial respiration	0-50	forest	34.03 N	12.9	98	NH_4NO_3	150	10	0.1628	0.0089	
Nowinsk et al. 2009	East of Los Angeles, USA	microbial respiration	O-horizon	forest	34.03 N	10.6	90	NH_4NO_3	50	10	0.7989	0.0478	
Nowinsk et al. 2009	East of Los Angeles, USA	microbial respiration	0-50	forest	34.03 N	12.9	98	NH_4NO_3	150	10	-0.0876	0.0386	
Maljanen et al. 2006	Southern Finland	microbial respiration	0-5	forest	61.19 N	3.3	680	NH_4NO_3	200	3	-0.0953	0.0322	
Verburg et al. 2004	EcoCELL, Kansas, USA	plant aboveground part C		grassland	39.31 N	12	188	NH_4	88	1	0.6818	0.0019	total
Verburg et al. 2004	EcoCELL, Kansas, USA	plant belowground part C		grassland	39.31 N	12	188	NH_4	88	1	0.4858	0.0043	total

Lovelock et al. 2007	Waikopua, Auckland, New Zealand	soil respiration		wetland	36.56 S	15.1	1212	urea		5	0.1137	0.1656
Lovelock et al. 2007	Waikopua, Auckland, New Zealand	soil respiration		wetland	36.56 S	15.1	1212	urea		5	0.2126	0.0472
Lovelock et al. 2007	Whangapoua, New Zealand	soil respiration		wetland	36.43 S	15.1	1212	urea		3	0.0894	0.2163
Lovelock et al. 2007	Whangapoua, New Zealand	soil respiration		wetland	36.43 S	15.1	1212	urea		3	0.0675	0.1653
Johnson et al. 2006	Placerville, California, USA	soil total C	0-30	forest	38.44 N	14.1	980	NH_4	100	6	0.0065	0.0022
Johnson et al. 2006	Placerville, California, USA	soil total C	0-31	forest	38.44 N	14.1	980	NH_4	200	6	-0.0671	0.0009
Jones et al 2006	Edinburgh, Scotland	soil respiration		grassland	55.52 N	8.3	849	NH_4NO_3	300	3	0.138	0.0139
Jones et al 2006	Edinburgh, Scotland	soil respiration		grassland	55.52 N	8.3	849	urea	300	3	0.2569	0.0239
Jones et al 2006	Edinburgh, Scotland	soil total C	0-2.5	grassland	55.52 N	8.3	849	NH_4NO_3	300	3	0.0517	0.0032
Jones et al 2006	Edinburgh, Scotland	soil total C	2.5-5	grassland	55.52 N	8.3	849	NH_4NO_3	300	3	-0.0279	0.0055
Jones et al 2006	Edinburgh, Scotland	soil total C	5-10	grassland	55.52 N	8.3	849	NH_4NO_3	300	3	0.0114	0.0096
Jones et al 2006	Edinburgh, Scotland	soil total C	10-15	grassland	55.52 N	8.3	849	NH_4NO_3	300	3	-0.1284	0.0192
Jones et al 2006	Edinburgh, Scotland	soil total C	15-20	grassland	55.52 N	8.3	849	NH_4NO_3	300	3	-0.042	0.011
Jones et al 2006	Edinburgh, Scotland	soil total C	20-40	grassland	55.52 N	8.3	849	urea	300	3	-0.0377	0.0353
Jones et al 2006	Edinburgh, Scotland	soil total C	0-2.5	grassland	55.52 N	8.3	849	urea	300	3	0.1089	0.0047
Jones et al 2006	Edinburgh, Scotland	soil total C	2.5-5	grassland	55.52 N	8.3	849	urea	300	3	0.2645	0.0415
Jones et al 2006	Edinburgh, Scotland	soil total C	5-10	grassland	55.52 N	8.3	849	urea	300	3	0.0227	0.0096
Jones et al 2006	Edinburgh, Scotland	soil total C	10-15	grassland	55.52 N	8.3	849	urea	300	3	-0.0368	0.0201
Jones et al 2006	Edinburgh, Scotland	soil total C	15-20	grassland	55.52 N	8.3	849	urea	300	3	0.0663	0.0197
Jones et al 2006	Edinburgh, Scotland	soil total C	20-40	grassland	55.52 N	8.3	849	urea	300	3	-0.0187	0.0394
Ambus and Robertson 2006	Michigan, USA	soil respiration		forest	42.24 N	9.7	890	NH_4NO_3	10	2	-0.0369	0.0159
Ambus and Robertson 2006	Michigan, USA	soil respiration		forest	42.24 N	9.7	890	NH_4NO_3	30	2	-0.0307	0.0112
Ambus and Robertson 2006	Michigan, USA	soil respiration		forest	42.24 N	9.7	890	NH_4NO_3	10	2	0.0101	0.0094
Ambus and Robertson 2006	Michigan, USA	soil respiration		forest	42.24 N	9.7	890	NH_4NO_3	30	2	0.0592	0.0098
Gulledge and Schimel 2000	Fairbanks, Alaska, USA	soil respiration		forest	64.45 N	-5.4	190	NH_4NO_3	66.7	4	-0.3137	0.0175
Gulledge and Schimel 2000	Fairbanks, Alaska, USA	soil respiration		forest	64.45 N	-5.4	190	NH_4NO_3	42.3	4	-0.1431	0.0117

Gulledge and Schimel 2000	Fairbanks, Alaska, USA	soil respiration		forest	64.45 N	-5.4	190	NH_4NO_3	171.4	4	0.1192	0.0046	
Gulledge and Schimel 2000	Fairbanks, Alaska, USA	soil respiration		forest	64.45 N	-5.4	190	NH_4NO_3	142.9	4	0.1457	0.0138	
McDowell et al. 2004	Harvard Forest, Massachusetts, USA	DOC		forest	42.3 N	6	1100	NH_4NO_3	50	10	-0.0426	0.0295	
McDowell et al. 2004	Harvard Forest, Massachusetts, USA	DOC		forest	42.3 N	6	1100	NH_4NO_3	150	10	0.0475	0.0385	
McDowell et al. 2004	Harvard Forest, Massachusetts, USA	DOC		forest	42.3 N	6	1100	NH_4NO_3	50	10	0.0835	0.0496	
McDowell et al. 2004	Harvard Forest, Massachusetts, USA	DOC		forest	42.3 N	6	1100	NH_4NO_3	150	10	0.2858	0.0358	
Pregitzer et al. 2004	Michigan, USA	DOC	0-75	forest	46.52 N	4.8	821	NO_3	30	7	1.3991	0.0401	
Pregitzer et al. 2004	Michigan, USA	DOC	0-75	forest	45.33 N	6.1	828	NO_3	30	7	0.8983	0.0709	
Pregitzer et al. 2004	Michigan, USA	DOC	0-75	forest	44.23 N	6.9	856	NO_3	30	7	0.8197	0.0712	
Pregitzer et al. 2004	Michigan, USA	DOC	0-75	forest	43.4 N	7.6	793	NO_3	30	7	1.706	0.0391	
Sinsabaugh et al. 2004	Lower Michigan, USA	DOC	0-20	forest	44.14 N	7.2	810	NO_3	80	1	0.1152	0.0347	
Sinsabaugh et al. 2004	Lower Michigan, USA	DOC	0-20	forest	44.14 N	7.2	810	NO_3	80	1	0.1226	0.0313	
Sinsabaugh et al. 2004	Lower Michigan, USA	DOC	0-20	forest	44.14 N	7.2	810	NO_3	80	1	0.4034	0.0593	
Sinsabaugh et al. 2004	Lower Michigan, USA	DOC	0-20	forest	44.14 N	7.2	810	NO_3	80	1	0.0612	0.1207	
Sinsabaugh et al. 2004	Lower Michigan, USA	DOC	0-20	forest	44.14 N	7.2	810	NO_3	80	1	0.1221	0.0841	
Sinsabaugh et al. 2004	Lower Michigan, USA	DOC	0-20	forest	44.14 N	7.2	810	NO_3	80	1	0.091	0.065	
Sinsabaugh et al. 2004	Lower Michigan, USA	DOC	0-20	forest	44.14 N	7.2	810	NO_3	80	1	0.47	0.0518	
Sinsabaugh et al. 2004	Lower Michigan, USA	DOC	0-20	forest	44.14 N	7.2	810	NO_3	80	1	0.1897	0.0451	
Sinsabaugh et al. 2004	Lower Michigan, USA	DOC	0-20	forest	44.14 N	7.2	810	NO_3	80	1	0.3531	0.0509	
Pregitzer et al. 2008	Michigan, USA	ANPP		forest	46.52 N	4.7	873	NO_3	30	10	0.1683	0.0013	woody
Pregitzer et al. 2008	Michigan, USA	ANPP		forest	45.33 N	6	871	NO_3	30	10	0.185	0.0161	woody
Pregitzer et al. 2008	Michigan, USA	ANPP		forest	44.23 N	6.9	888	NO_3	30	10	0.1394	0.013	woody
Pregitzer et al. 2008	Michigan, USA	ANPP		forest	43.4 N	7.6	812	NO_3	30	10	0.1224	0.0231	woody
Pregitzer et al. 2008	Michigan, USA	litter C		forest	46.52 N	4.7	873	NO_3	30	10	0.0063	0.0003	
Pregitzer et al. 2008	Michigan, USA	litter C		forest	45.33 N	6	871	NO_3	30	10	0	0.0006	
Pregitzer et al. 2008	Michigan, USA	litter C		forest	44.23 N	6.9	888	NO_3	30	10	0	0.0014	

Pregitzer et al. 2008	Michigan, USA	litter C		forest	43.4 N	7.6	812	NO_3	30	10	0.0655	0.0059
Pregitzer et al. 2008	Michigan, USA	soil total C	O-horizon	forest	46.52 N	4.7	873	NO_3	30	10	-0.0023	0.002
Pregitzer et al. 2008	Michigan, USA	soil total C	O-horizon	forest	45.33 N	6	871	NO_3	30	10	-0.0066	0.0002
Pregitzer et al. 2008	Michigan, USA	soil total C	O-horizon	forest	44.23 N	6.9	888	NO_3	30	10	-0.0413	0.0019
Pregitzer et al. 2008	Michigan, USA	soil total C	O-horizon	forest	43.4 N	7.6	812	NO_3	30	10	0.0065	0.001
Pregitzer et al. 2008	Michigan, USA	soil total C	0-10	forest	46.52 N	4.7	873	NO_3	30	10	-0.0083	0.0511
Pregitzer et al. 2008	Michigan, USA	soil total C	0-10	forest	45.33 N	6	871	NO_3	30	10	0.6931	0.0534
Pregitzer et al. 2008	Michigan, USA	soil total C	0-10	forest	44.23 N	6.9	888	NO_3	30	10	0.0323	0.0132
Pregitzer et al. 2008	Michigan, USA	soil total C	0-10	forest	43.4 N	7.6	812	NO_3	30	10	0.3331	0.0384
Pregitzer et al. 2008	Michigan, USA	soil total C	10-30	forest	46.52 N	4.7	873	NO_3	30	10	0.0241	0.0414
Pregitzer et al. 2008	Michigan, USA	soil total C	10-30	forest	45.33 N	6	871	NO_3	30	10	-0.1335	0.0122
Pregitzer et al. 2008	Michigan, USA	soil total C	10-30	forest	44.23 N	6.9	888	NO_3	30	10	-0.0194	0.0172
Pregitzer et al. 2008	Michigan, USA	soil total C	10-30	forest	43.4 N	7.6	812	NO_3	30	10	-0.0347	0.0164
Pregitzer et al. 2008	Michigan, USA	soil total C	30-50	forest	46.52 N	4.7	873	NO_3	30	10	0.2113	0.6692
Pregitzer et al. 2008	Michigan, USA	soil total C	30-50	forest	45.33 N	6	871	NO_3	30	10	0	0.6646
Pregitzer et al. 2008	Michigan, USA	soil total C	30-50	forest	44.23 N	6.9	888	NO_3	30	10	0	0.6743
Pregitzer et al. 2008	Michigan, USA	soil total C	30-50	forest	43.4 N	7.6	812	NO_3	30	10	-0.398	0.6727
Pregitzer et al. 2008	Michigan, USA	soil total C	50-70	forest	46.52 N	4.7	873	NO_3	30	10	-0.0606	0.6636
Pregitzer et al. 2008	Michigan, USA	soil total C	50-70	forest	45.33 N	6	871	NO_3	30	10	-0.0606	0.6636
Pregitzer et al. 2008	Michigan, USA	soil total C	50-70	forest	44.23 N	6.9	888	NO_3	30	10	0.2364	0.6952
Pregitzer et al. 2008	Michigan, USA	soil total C	50-70	forest	43.4 N	7.6	812	NO_3	30	10	-0.2877	0.6914
Smaill et al. 2008	Woodhill, New Zealand	soil total C	O-horizon	forest	36.43 S	14.3	1330	urea	150	18	-0.7449	0.0559
Smaill et al. 2008	Tarawera, New Zealand	soil total C	O-horizon	forest	38.13 S	14	1820	urea	115	10	-0.6678	0.0262
Smaill et al. 2008	Berwick, New Zealand	soil total C	O-horizon	forest	46 S	10.3	747	urea	95	10	-0.4353	0.0161
Smaill et al. 2008	Burnham, New Zealand	soil total C	O-horizon	forest	43.37 S	11.5	639	urea	98	10	-0.6054	0.0608
Smaill et al. 2008	Kinleith, New Zealand	soil total C	O-horizon	forest	38.14 S	13.2	1420	urea	136	7	-0.277	0.0137

Smaill et al. 2008	Golden Downs, New Zealand	soil total C	O-horizon	forest	41.36 S	10.4	1340	urea	142	6	-0.261	0.0512
Koehler et al. 2009b	Gigante Peninsula, Panama	soil total C	0-5	forest	9.06 N	27.4	2650	urea	125	6	-0.0774	0.0173
Koehler et al. 2009b	Gigante Peninsula, Panama	soil total C	5-50	forest	9.06 N	27.4	2650	urea	125	6	-0.0403	0.009
Mäkipää 1995	Heinola, Southern Finland	soil total C	0-10	forest	61.1 N	3.3	680	NH_4NO_3	28.5	25	0.9452	0.0129
Mäkipää 1995	Padasjoki, Southern Finland	soil total C	0-10	forest	61.24 N	3.3	680	NH_4NO_3	26	20	0.3069	0.12
Mäkipää 1995	Punkaharju, Southern Finland	soil total C	0-10	forest	61.49 N	3.3	680	NH_4NO_3	26	30	-0.1007	0.1026
Mäkipää 1995	Heinola, Southern Finland	soil total C	0-10	forest	61.6 N	3.3	680	NH_4NO_3	23	26	0.2453	0.0042
Mäkipää 1995	Halsua, Southern Finland	soil total C	0-10	forest	63.23 N	3.3	680	NH_4NO_3	33	28	0.3992	0.1869
Mäkipää 1995	Heinola, Southern Finland	soil total C	O-horizon	forest	61.1 N	3.3	680	NH_4NO_3	28.5	25	0.5874	0.0336
Mäkipää 1995	Padasjoki, Southern Finland	soil total C	O-horizon	forest	61.24 N	3.3	680	NH_4NO_3	26	20	-0.3238	0.0429
Mäkipää 1995	Punkaharju, Southern Finland	soil total C	O-horizon	forest	61.49 N	3.3	680	NH_4NO_3	26	30	0.128	0.0319
Mäkipää 1995	Heinola, Southern Finland	soil total C	O-horizon	forest	61.6 N	3.3	680	NH_4NO_3	23	26	0.1339	0.0222
Mäkipää 1995	Halsua, Southern Finland	soil total C	O-horizon	forest	63.23 N	3.3	680	NH_4NO_3	33	28	0.6509	0.0352
Mäkipää 1995	Heinola, Southern Finland	soil total C	10-30	forest	61.1 N	3.3	680	NH_4NO_3	28.5	25	0.9816	0.051
Mäkipää 1995	Padasjoki, Southern Finland	soil total C	10-30	forest	61.24 N	3.3	680	NH_4NO_3	26	20	0.2163	0.1183
Mäkipää 1995	Punkaharju, Southern Finland	soil total C	10-30	forest	61.49 N	3.3	680	NH_4NO_3	26	30	-0.2273	0.0828
Mäkipää 1995	Heinola, Southern Finland	soil total C	10-30	forest	61.6 N	3.3	680	NH_4NO_3	23	26	-0.1279	0.0598
Mäkipää 1995	Halsua, Southern Finland	soil total C	10-30	forest	63.23 N	3.3	680	NH_4NO_3	33	28	0.477	0.2866
Yano et al. 2000	Harvard Forest, USA	DOC	O-horizon	forest	42.3 N	6.1	1090	NH_4NO_3	50	6	0.0974	0.0137
Yano et al. 2000	Harvard Forest, USA	DOC	O-horizon	forest	42.3 N	6.1	1090	NH_4NO_3	150	6	0.159	0.0127
Yano et al. 2000	Harvard Forest, USA	DOC	O-horizon	forest	42.3 N	6.1	1090	NH_4NO_3	50	6	0.171	0.0113
Yano et al. 2000	Harvard Forest, USA	DOC	O-horizon	forest	42.3 N	6.1	1090	NH_4NO_3	150	6	0.2681	0.0105
Reid et al. 2012	Minnesota, USA	soil total C	0-20	grassland	45 N	5.5	660	NH_4NO_3	40	3	0.0707	0.007
Du et al. 2014a	Shijiazhuang, China	soil respiration		forest	38.41 N	13.1	650	urea, NH_4NO_3	48	2	-0.0435	0.0054
Du et al. 2014a	Shijiazhuang, China	soil respiration		forest	38.41 N	13.1	650	urea,	48	2	-0.209	0.0083

								NH_4NO_3					
Du et al. 2014a	Shijiazhuang, China	soil respiration		forest	38.41 N	13.1	650	urea, NH_4NO_3	48	2	-0.4746	0.0115	
Du et al. 2014a	Shijiazhuang, China	soil respiration		forest	38.41 N	13.1	650	urea, NH_4NO_3	48	2	-0.6391	0.0119	
Du et al. 2014a	Shijiazhuang, China	soil respiration		forest	38.41 N	13.1	650	NH_4NO_3	48	2	-1.0214	0.0109	
Heijmans et al. 2001	State Forest of Dwingeloo, Netherlands	plant aboveground part C		wetland	51.99 N	9	840	NH_4NO_3	50	3	0.3462	0.0556	vascular plants
Heijmans et al. 2001	State Forest of Dwingeloo, Netherlands	litter C		wetland	51.99 N	9	840	NH_4NO_3	50	3	0.9316	0.3088	
Heijmans et al. 2001	State Forest of Dwingeloo, Netherlands	ANPP		wetland	51.99 N	9	840	NH_4NO_3	50	3	0.3687	0.0396	vascular plants
Heijmans et al. 2001	State Forest of Dwingeloo, Netherlands	ANPP		wetland	51.99 N	9	840	NH_4NO_3	50	3	-0.3782	0.0236	moss
Heijmans et al. 2001	State Forest of Dwingeloo, Netherlands	plant belowground part C		wetland	51.99 N	9	840	NH_4NO_3	50	3	0.2509	0.0616	vascular plants
Wang et al. 2013	Sanjiang Plain, Heilongjiang, China	ER		wetland	47.49 N	2.1	600	NH_4NO_3	80	2	0.0359	0.0048	
Wang et al. 2013	Sanjiang Plain, Heilongjiang, China	GPP		wetland	47.49 N	2.1	600	NH_4NO_3	80	2	0.0909	0.0027	
Wang et al. 2009a	Guangdong, China	MBC	0-10	forest	23.08 N	20.9	1927	NH_4NO_3	50	3	0.0997	0.0031	
Wang et al. 2009a	Guangdong, China	MBC	0-10	forest	23.08 N	20.9	1927	NH_4NO_3	100	3	-0.0412	0.0009	
Wang et al. 2009a	Guangdong, China	MBC	0-10	forest	23.08 N	20.9	1927	NH_4NO_3	50	3	0.1343	0.0053	
Wang et al. 2009a	Guangdong, China	MBC	0-10	forest	23.08 N	20.9	1927	NH_4NO_3	100	3	-0.1032	0.0046	
Wang et al. 2009a	Guangdong, China	MBC	0-10	forest	23.08 N	20.9	1927	NH_4NO_3	50	3	-0.032	0.0011	
Wang et al. 2009a	Guangdong, China	MBC	0-10	forest	23.08 N	20.9	1927	NH_4NO_3	100	3	-0.0604	0.001	
Wang et al. 2009a	Guangdong, China	MBC	0-10	forest	23.08 N	20.9	1927	NH_4NO_3	150	3	-0.2153	0.0009	
Wang et al. 2009a	Guangdong, China	DOC	0-10	forest	23.08 N	20.9	1927	NH_4NO_3	50	3	0.0623	0.0019	
Wang et al. 2009a	Guangdong, China	DOC	0-10	forest	23.08 N	20.9	1927	NH_4NO_3	100	3	-0.0603	0.0032	
Wang et al. 2009a	Guangdong, China	DOC	0-10	forest	23.08 N	20.9	1927	NH_4NO_3	50	3	0.0677	0.0033	

Wang et al. 2009a	Guangdong, China	DOC	0-10	forest	23.08 N	20.9	1927	NH_4NO_3	100	3	0.1311	0.0059	
Wang et al. 2009a	Guangdong, China	DOC	0-10	forest	23.08 N	20.9	1927	NH_4NO_3	50	3	0.0189	0.0008	
Wang et al. 2009a	Guangdong, China	DOC	0-10	forest	23.08 N	20.9	1927	NH_4NO_3	100	3	0.1082	0.0011	
Wang et al. 2009a	Guangdong, China	DOC	0-10	forest	23.08 N	20.9	1927	NH_4NO_3	150	3	0.1501	0.0008	
Chen et al. 2012a	Guangdong, China	soil TOC	0-10	forest	23.1 N	21.5	1956	NH_4NO_3	50	6	0.0766	0.0573	
Chen et al. 2012a	Guangdong, China	soil TOC	0-10	forest	23.1 N	21.5	1956	NH_4NO_3	100	6	0.1401	0.0408	
Chen et al. 2012a	Guangdong, China	soil TOC	10-20	forest	23.1 N	21.5	1956	NH_4NO_3	50	6	0.08	0.0136	
Chen et al. 2012a	Guangdong, China	soil TOC	10-20	forest	23.1 N	21.5	1956	NH_4NO_3	100	6	-0.0572	0.0065	
Chen et al. 2012a	Guangdong, China	soil TOC	0-10	forest	23.1 N	21.5	1956	NH_4NO_3	50	6	0.0766	0.0573	
Chen et al. 2012a	Guangdong, China	soil TOC	0-10	forest	23.1 N	21.5	1956	NH_4NO_3	100	6	0.1401	0.0408	
Chen et al. 2012a	Guangdong, China	soil TOC	10-20	forest	23.1 N	21.5	1956	NH_4NO_3	50	6	0.08	0.0136	
Chen et al. 2012a	Guangdong, China	soil TOC	10-20	forest	23.1 N	21.5	1956	NH_4NO_3	100	6	-0.0572	0.0065	
Chen et al. 2012a	Guangdong, China	soil TOC	0-10	forest	23.1 N	21.5	1956	NH_4NO_3	50	6	-0.071	0.0345	
Chen et al. 2012a	Guangdong, China	soil TOC	0-10	forest	23.1 N	21.5	1956	NH_4NO_3	100	6	0.1812	0.0243	
Chen et al. 2012a	Guangdong, China	soil TOC	0-10	forest	23.1 N	21.5	1956	NH_4NO_3	150	6	0.1982	0.0223	
Chen et al. 2012a	Guangdong, China	soil TOC	10-20	forest	23.1 N	21.5	1956	NH_4NO_3	50	6	-0.0121	0.0378	
Chen et al. 2012a	Guangdong, China	soil TOC	10-20	forest	23.1 N	21.5	1956	NH_4NO_3	100	6	0.092	0.0469	
Chen et al. 2012a	Guangdong, China	soil TOC	10-20	forest	23.1 N	21.5	1956	NH_4NO_3	150	6	0.0471	0.0456	
Chen et al. 2012a	Guangdong, China	MBC	0-10	forest	23.1 N	21.5	1956	NH_4NO_3	50	6	0.0846	0.1014	
Chen et al. 2012a	Guangdong, China	MBC	0-10	forest	23.1 N	21.5	1956	NH_4NO_3	100	6	0	0.1019	
Chen et al. 2012a	Guangdong, China	MBC	0-10	forest	23.1 N	21.5	1956	NH_4NO_3	50	6	0.0712	0.0803	
Chen et al. 2012a	Guangdong, China	MBC	0-10	forest	23.1 N	21.5	1956	NH_4NO_3	100	6	-0.0678	0.0615	
Chen et al. 2012a	Guangdong, China	MBC	0-10	forest	23.1 N	21.5	1956	NH_4NO_3	50	6	-0.0353	0.0474	
Chen et al. 2012a	Guangdong, China	MBC	0-10	forest	23.1 N	21.5	1956	NH_4NO_3	100	6	-0.1427	0.0516	
Chen et al. 2012a	Guangdong, China	MBC	0-10	forest	23.1 N	21.5	1956	NH_4NO_3	150	6	-0.248	0.037	
Bragazza et al. 2012	Italian Alps	ANPP		wetland	46.21 N	3	1000	NH_4NO_3	30	7	-0.9662	0.5254	moss

Bragazza et al. 2012	Italian Alps	ANPP		wetland	46.21 N	3	1000	NH_4NO_3	30	7	0.2595	0.1205		herbaceous
Bragazza et al. 2012	Italian Alps	ANPP		wetland	46.21 N	3	1000	NH_4NO_3	30	7	0.8863	0.0773		woody
Bragazza et al. 2012	Italian Alps	litter decomposition rate		wetland	46.21 N	3	1000	NH_4NO_3	30	3	0.0497	0.0034	*Calluna*	woody
Bragazza et al. 2012	Italian Alps	litter decomposition rate		wetland	46.21 N	3	1000	NH_4NO_3	30	3	0.1792	0.0254	*Sphagnum*	moss
Bragazza et al. 2012	Italian Alps	litter decomposition rate		wetland	46.21 N	3	1000	NH_4NO_3	30	3	-0.0912	0.0082	*Eriophorum*	herbaceous
Fang et al. 2014	Qinghai, China	DOC	0-10	grassland	37.37 N	-0.4	383.3	NH_4	10	5	0.2371	0.0146		
Fang et al. 2014	Qinghai, China	DOC	0-10	grassland	37.37 N	-0.4	383.3	NH_4	10	5	0.0879	0.0014		
Fang et al. 2014	Qinghai, China	DOC	0-10	grassland	37.37 N	-0.4	383.3	NO_3	10	5	0.3007	0.0018		
Fang et al. 2014	Qinghai, China	DOC	0-10	grassland	37.37 N	-0.4	383.3	NH_4	20	5	0.2909	0.0152		
Fang et al. 2014	Qinghai, China	DOC	0-10	grassland	37.37 N	-0.4	383.3	NH_4	20	5	0.1789	0.0166		
Fang et al. 2014	Qinghai, China	DOC	0-10	grassland	37.37 N	-0.4	383.3	NO_3	20	5	0.2813	0.0226		
Fang et al. 2014	Qinghai, China	DOC	0-10	grassland	37.37 N	-0.4	383.3	NH_4	40	5	0.2107	0.0095		
Fang et al. 2014	Qinghai, China	DOC	0-10	grassland	37.37 N	-0.4	383.3	NH_4	40	5	0.1447	0.0031		
Fang et al. 2014	Qinghai, China	DOC	0-10	grassland	37.37 N	-0.4	383.3	NO_3	40	5	0.262	0.0184		
Fang et al. 2014	Qinghai, China	DOC	10-20	grassland	37.37 N	-0.4	383.3	NH_4	10	5	-0.1507	0.0041		
Fang et al. 2014	Qinghai, China	DOC	10-20	grassland	37.37 N	-0.4	383.3	NH_4	10	5	-0.1099	0.0042		
Fang et al. 2014	Qinghai, China	DOC	10-20	grassland	37.37 N	-0.4	383.3	NO_3	10	5	-0.2715	0.0024		
Fang et al. 2014	Qinghai, China	DOC	10-20	grassland	37.37 N	-0.4	383.3	NH_4	20	5	-0.2005	0.0019		
Fang et al. 2014	Qinghai, China	DOC	10-20	grassland	37.37 N	-0.4	383.3	NH_4	20	5	-0.199	0.007		
Fang et al. 2014	Qinghai, China	DOC	10-20	grassland	37.37 N	-0.4	383.3	NO_3	20	5	-0.2052	0.0028		
Fang et al. 2014	Qinghai, China	DOC	10-20	grassland	37.37 N	-0.4	383.3	NH_4	40	5	-0.1451	0.0036		
Fang et al. 2014	Qinghai, China	DOC	10-20	grassland	37.37 N	-0.4	383.3	NH_4	40	5	-0.1594	0.0024		
Fang et al. 2014	Qinghai, China	DOC	10-20	grassland	37.37 N	-0.4	383.3	NO_3	40	5	-0.2288	0.0064		
Xu et al. 2004	Qinghai, China	soil respiration		grassland	37.36 N	-1.7	560	NO_3	44	1	0.3699	0.0041		
Xu et al. 2004	Qinghai, China	soil respiration		grassland	37.36 N	-1.7	560	NH_4	56	1	-0.0502	0.0046		
Xu et al. 2004	Qinghai, China	soil respiration		grassland	38 N	-1.7	560	NO_3	44	1	-0.8174	0.016		

Xu et al. 2004	Qinghai, China	soil respiration		grassland	38 N	-1.7	560	NH_4	56	1	0.178	0.0214	
Xu et al. 2004	Qinghai, China	plant belowground part C		grassland	37.52 N	-1.7	560	NO_3	44	1	0.5653	0.0175	total
Xu et al. 2004	Qinghai, China	plant belowground part C		grassland	37.52 N	-1.7	560	NH_4	56	1	0.2783	0.0093	total
Xu et al. 2004	Qinghai, China	plant aboveground part C		grassland	37.52 N	-1.7	560	NO_3	44	1	-0.0769	0.0498	total
Xu et al. 2004	Qinghai, China	plant aboveground part C		grassland	37.52 N	-1.7	560	NH_4	56	1	-0.2736	0.0909	total
Du et al. 2014b	Heibei, China	MBC	0-10	ecotone	42.5 N	-1.4	450	urea	50	3	0.1719	0.042	
Du et al. 2014b	Heibei, China	MBC	0-10	ecotone	42.5 N	-1.4	450	urea	100	3	0.2006	0.0478	
Du et al. 2014b	Heibei, China	MBC	0-10	ecotone	42.5 N	-1.4	450	urea	150	3	0.0935	0.0494	
Du et al. 2014b	Heibei, China	NPP		ecotone	42.5 N	-1.4	450	urea	50	3	0.1379	0.0025	
Du et al. 2014b	Heibei, China	NPP		ecotone	42.5 N	-1.4	450	urea	100	3	0.2047	0.0024	
Du et al. 2014b	Heibei, China	NPP		ecotone	42.5 N	-1.4	450	urea	150	3	0.3364	0.0024	
Du et al. 2014b	Heibei, China	soil respiration		ecotone	42.5 N	-1.4	450	urea	50	3	-0.0074	0.0047	
Du et al. 2014b	Heibei, China	soil respiration		ecotone	42.5 N	-1.4	450	urea	100	3	0.0364	0.0052	
Du et al. 2014b	Heibei, China	soil respiration		ecotone	42.5 N	-1.4	450	urea	150	3	-0.0455	0.0058	
Du et al. 2014b	Heibei, China	microbial respiration	0-10	ecotone	42.5 N	-1.4	450	urea	50	3	0.351	0.2226	
Du et al. 2014b	Heibei, China	microbial respiration	0-10	ecotone	42.5 N	-1.4	450	urea	100	3	-0.2752	0.2128	
Du et al. 2014b	Heibei, China	microbial respiration	0-10	ecotone	42.5 N	-1.4	450	urea	150	3	0.5759	0.2235	
Liu and Crowley 2009	Lake Mathews, California, USA	soil total C	0-10	grassland	33.51 N	13.5	280	NO_3	50	2	0	0.0118	
Liu and Crowley 2009	Mott Rimrock Reserve, California, USA	soil total C	0-10	grassland	33.48 N	13.5	280	NO_3	50	2	0.0196	0.0058	
Liu and Crowley 2009	Santa Margarita Ecological Reserve, California, USA	soil total C	0-10	grassland	33.29 N	13.5	280	NO_3	50	2	-0.008	0.0096	
Fan et al. 2007	Fujian, China	soil total C	0-20	forest	26.3 N	19	1600	urea	60	3	-0.0333	0.0018	
Fan et al. 2007	Fujian, China	soil total C	0-20	forest	26.3 N	19	1600	urea	120	3	-0.0846	0.001	

Fan et al. 2007	Fujian, China	soil total C	0-20	forest	26.3 N	19	1600	urea	240	3	-0.1607	0.0018
Fan et al. 2007	Fujian, China	soil total C	20-40	forest	26.3 N	19	1600	urea	60	3	-0.0594	0.0039
Fan et al. 2007	Fujian, China	soil total C	20-40	forest	26.3 N	19	1600	urea	120	3	-0.0694	0.0031
Fan et al. 2007	Fujian, China	soil total C	20-40	forest	26.3 N	19	1600	urea	240	3	-0.1582	0.0063
Fan et al. 2007	Fujian, China	soil total C	40-60	forest	26.3 N	19	1600	urea	60	3	-0.0622	0.0047
Fan et al. 2007	Fujian, China	soil total C	40-60	forest	26.3 N	19	1600	urea	120	3	-0.2053	0.0042
Fan et al. 2007	Fujian, China	soil total C	40-60	forest	26.3 N	19	1600	urea	240	3	-0.2746	0.0023
Dou et al. 2008	Sanjiang Plain, Heilongjiang, China	MBC	0-35	wetland	47.35 N	1.9	600	NH_4	10	1	0.151	0.0186
Dou et al. 2008	Sanjiang Plain, Heilongjiang, China	MBC	0-35	wetland	47.35 N	1.9	600	NH_4	30	1	0.1974	0.0187
Dou et al. 2008	Sanjiang Plain, Heilongjiang, China	MBC	0-35	wetland	47.35 N	1.9	600	NH_4	50	1	0.2401	0.0201
Dou et al. 2008	Sanjiang Plain, Heilongjiang, China	DOC	0-35	wetland	47.35 N	1.9	600	NH_4	10	1	0.0569	0.0313
Dou et al. 2008	Sanjiang Plain, Heilongjiang, China	DOC	0-35	wetland	47.35 N	1.9	600	NH_4	30	1	0.1786	0.029
Dou et al. 2008	Sanjiang Plain, Heilongjiang, China	DOC	0-35	wetland	47.35 N	1.9	600	NH_4	50	1	0.2098	0.0305
Tu et al. 2010	Ya'an, Sichuan, China	soil total C	0-20	forest	30.08 N	16.1	1772	NH_4NO_3	50	2	-0.0513	0.0001
Tu et al. 2010	Ya'an, Sichuan, China	soil total C	0-20	forest	30.08 N	16.1	1772	NH_4NO_3	150	2	-0.0516	0
Tu et al. 2010	Ya'an, Sichuan, China	soil total C	0-20	forest	30.08 N	16.1	1772	NH_4NO_3	300	2	-0.0591	0.0001
Tu et al. 2010	Ya'an, Sichuan, China	soil total C	20-40	forest	30.08 N	16.1	1772	NH_4NO_3	50	2	0.0064	0.0002
Tu et al. 2010	Ya'an, Sichuan, China	soil total C	20-40	forest	30.08 N	16.1	1772	NH_4NO_3	150	2	-0.0039	0.0003
Tu et al. 2010	Ya'an, Sichuan, China	soil total C	20-40	forest	30.08 N	16.1	1772	NH_4NO_3	300	2	-0.0244	0.0002
Tu et al. 2010	Ya'an, Sichuan, China	soil total C	40-60	forest	30.08 N	16.1	1772	NH_4NO_3	50	2	-0.051	0.0004
Tu et al. 2010	Ya'an, Sichuan, China	soil total C	40-60	forest	30.08 N	16.1	1772	NH_4NO_3	150	2	-0.0517	0.0001
Tu et al. 2010	Ya'an, Sichuan, China	soil total C	40-60	forest	30.08 N	16.1	1772	NH_4NO_3	300	2	-0.0521	0.0001
Tu et al. 2010	Ya'an, Sichuan, China	soil total C	60-80	forest	30.08 N	16.1	1772	NH_4NO_3	50	2	-0.0006	0.0002
Tu et al. 2010	Ya'an, Sichuan, China	soil total C	60-80	forest	30.08 N	16.1	1772	NH_4NO_3	150	2	-0.045	0.0002
Tu et al. 2010	Ya'an, Sichuan, China	soil total C	60-80	forest	30.08 N	16.1	1772	NH_4NO_3	300	2	-0.1647	0.0001
Tu et al. 2010	Ya'an, Sichuan, China	MBC	0-20	forest	30.08 N	16.1	1772	NH_4NO_3	50	2	-0.2364	0.0001

Tu et al. 2010	Ya'an, Sichuan, China	MBC	0-20	forest	30.08 N	16.1	1772	NH_4NO_3	150	2	-0.4321	0.0001
Tu et al. 2010	Ya'an, Sichuan, China	MBC	0-20	forest	30.08 N	16.1	1772	NH_4NO_3	300	2	-0.3214	0.0001
Tu et al. 2010	Ya'an, Sichuan, China	MBC	20-40	forest	30.08 N	16.1	1772	NH_4NO_3	50	2	0.0323	0.0008
Tu et al. 2010	Ya'an, Sichuan, China	MBC	20-40	forest	30.08 N	16.1	1772	NH_4NO_3	150	2	-0.1406	0.0002
Tu et al. 2010	Ya'an, Sichuan, China	MBC	20-40	forest	30.08 N	16.1	1772	NH_4NO_3	300	2	-0.1889	0.0002
Tu et al. 2010	Ya'an, Sichuan, China	MBC	40-60	forest	30.08 N	16.1	1772	NH_4NO_3	50	2	-0.0556	0.0008
Tu et al. 2010	Ya'an, Sichuan, China	MBC	40-60	forest	30.08 N	16.1	1772	NH_4NO_3	150	2	0	0.0007
Tu et al. 2010	Ya'an, Sichuan, China	MBC	40-60	forest	30.08 N	16.1	1772	NH_4NO_3	300	2	0.0267	0.0007
Tu et al. 2010	Ya'an, Sichuan, China	MBC	60-80	forest	30.08 N	16.1	1772	NH_4NO_3	50	2	0.0827	0.0004
Tu et al. 2010	Ya'an, Sichuan, China	MBC	60-80	forest	30.08 N	16.1	1772	NH_4NO_3	150	2	0	0.0003
Tu et al. 2010	Ya'an, Sichuan, China	MBC	60-80	forest	30.08 N	16.1	1772	NH_4NO_3	300	2	0	0.0005
Tu et al. 2010	Ya'an, Sichuan, China	DOC	0-20	forest	30.08 N	16.1	1772	NH_4NO_3	50	2	0.2287	0.0008
Tu et al. 2010	Ya'an, Sichuan, China	DOC	0-20	forest	30.08 N	16.1	1772	NH_4NO_3	150	2	0.1933	0.0009
Tu et al. 2010	Ya'an, Sichuan, China	DOC	0-20	forest	30.08 N	16.1	1772	NH_4NO_3	300	2	0.304	0.0008
Tu et al. 2010	Ya'an, Sichuan, China	DOC	20-40	forest	30.08 N	16.1	1772	NH_4NO_3	50	2	0.2818	0.0011
Tu et al. 2010	Ya'an, Sichuan, China	DOC	20-40	forest	30.08 N	16.1	1772	NH_4NO_3	150	2	0.3979	0.001
Tu et al. 2010	Ya'an, Sichuan, China	DOC	20-40	forest	30.08 N	16.1	1772	NH_4NO_3	300	2	0.6069	0.0009
Tu et al. 2010	Ya'an, Sichuan, China	DOC	40-60	forest	30.08 N	16.1	1772	NH_4NO_3	50	2	0.2429	0.0007
Tu et al. 2010	Ya'an, Sichuan, China	DOC	40-60	forest	30.08 N	16.1	1772	NH_4NO_3	150	2	0.4039	0.0003
Tu et al. 2010	Ya'an, Sichuan, China	DOC	40-60	forest	30.08 N	16.1	1772	NH_4NO_3	300	2	0.3689	0.0003
Tu et al. 2010	Ya'an, Sichuan, China	DOC	60-80	forest	30.08 N	16.1	1772	NH_4NO_3	50	2	0.0936	0.0004
Tu et al. 2010	Ya'an, Sichuan, China	DOC	60-80	forest	30.08 N	16.1	1772	NH_4NO_3	150	2	0.1069	0.0002
Tu et al. 2010	Ya'an, Sichuan, China	DOC	60-80	forest	30.08 N	16.1	1772	NH_4NO_3	300	2	0.4261	0.0006
Zhang et al. 2013a	Sanjiang Plain, Heilongjiang, China	soil total C	0-10	wetland	47.43 N	1.9	585	NH_4NO_3	40	2	-0.0179	0.0006
Zhang et al. 2013a	Sanjiang Plain, Heilongjiang, China	soil total C	0-10	wetland	47.43 N	1.9	585	NH_4NO_3	80	2	-0.0044	0.0008
Zhang et al. 2013a	Sanjiang Plain, Heilongjiang, China	soil total C	10-20	wetland	47.43 N	1.9	585	NH_4NO_3	40	2	-0.0337	0.0003

Zhang et al. 2013a	Sanjiang Plain, Heilongjiang, China	soil total C	10-20	wetland	47.43 N	1.9	585	NH_4NO_3	80	2	-0.0167	0.0002
Zhang et al. 2013a	Sanjiang Plain, Heilongjiang, China	soil total C	20-30	wetland	47.43 N	1.9	585	NH_4NO_3	40	2	0.0448	0.0004
Zhang et al. 2013a	Sanjiang Plain, Heilongjiang, China	soil total C	20-30	wetland	47.43 N	1.9	585	NH_4NO_3	80	2	0.017	0.0005
Tu et al. 2011a	Hongya, Sichuan, China	soil total C	0-20	forest	29.95 N	15	1489	NH_4NO_3	50	2	0.0212	0.0004
Tu et al. 2011a	Hongya, Sichuan, China	soil total C	0-20	forest	29.95 N	15	1489	NH_4NO_3	150	2	0.0142	0.001
Tu et al. 2011a	Hongya, Sichuan, China	soil total C	0-20	forest	29.95 N	15	1489	NH_4NO_3	300	2	0.0142	0.0004
Tu et al. 2011a	Hongya, Sichuan, China	MBC	0-20	forest	29.95 N	15	1489	NH_4NO_3	50	2	0.08	0.0007
Tu et al. 2011a	Hongya, Sichuan, China	MBC	0-20	forest	29.95 N	15	1489	NH_4NO_3	150	2	0.0465	0.0007
Tu et al. 2011a	Hongya, Sichuan, China	MBC	0-20	forest	29.95 N	15	1489	NH_4NO_3	300	2	0.1439	0.0023
Volk et al. 2011	Canton Grisons, Switzerland	GPP		grassland	9.39 N	4.2	771	NH_4NO_3	10	1	-0.0136	0.0007
Volk et al. 2011	Canton Grisons, Switzerland	GPP		grassland	9.39 N	4.2	771	NH_4NO_3	50	1	-0.0236	0.0006
Volk et al. 2011	Canton Grisons, Switzerland	ER		grassland	9.39 N	4.2	771	NH_4NO_3	10	1	-0.0027	0.0008
Volk et al. 2011	Canton Grisons, Switzerland	ER		grassland	9.39 N	4.2	771	NH_4NO_3	50	1	0.0553	0.0011
Tu et al. 2011b	Liujiang, Sichuan, China	soil respiration		forest	29.95 N	16.1	1490	NH_4NO_3	50	2	0.1023	0.0174
Tu et al. 2011b	Liujiang, Sichuan, China	soil respiration		forest	29.95 N	16.1	1490	NH_4NO_3	150	2	0.1563	0.0123
Tu et al. 2011b	Liujiang, Sichuan, China	soil respiration		forest	29.95 N	16.1	1490	NH_4NO_3	300	2	0.2643	0.0106
Tu et al. 2011b	Liujiang, Sichuan, China	NPP		forest	29.95 N	16.1	1490	NH_4NO_3	50	2	0.0765	0.0079
Tu et al. 2011b	Liujiang, Sichuan, China	NPP		forest	29.95 N	16.1	1490	NH_4NO_3	150	2	0.113	0.008
Tu et al. 2011b	Liujiang, Sichuan, China	NPP		forest	29.95 N	16.1	1490	NH_4NO_3	300	2	0.3154	0.0066
Tu et al. 2011b	Liujiang, Sichuan, China	NEP		forest	29.95 N	16.1	1490	NH_4NO_3	50	2	0.0479	0.0144
Tu et al. 2011b	Liujiang, Sichuan, China	NEP		forest	29.95 N	16.1	1490	NH_4NO_3	150	2	0.0628	0.0153
Tu et al. 2011b	Liujiang, Sichuan, China	NEP		forest	29.95 N	16.1	1490	NH_4NO_3	300	2	0.3728	0.0101
Tu et al. 2011b	Liujiang, Sichuan, China	MBC	0-20	forest	29.95 N	16.1	1490	NH_4NO_3	50	2	0.081	0.0024
Tu et al. 2011b	Liujiang, Sichuan, China	MBC	0-20	forest	29.95 N	16.1	1490	NH_4NO_3	150	2	0.0585	0.0018
Tu et al. 2011b	Liujiang, Sichuan, China	MBC	0-20	forest	29.95 N	16.1	1490	NH_4NO_3	300	2	0.1661	0.0022
Tu et al. 2011b	Liujiang, Sichuan, China	DOC	0-20	forest	29.95 N	16.1	1490	NH_4NO_3	50	2	0.0514	0.0046

Tu et al. 2011b	Liujiang, Sichuan, China	DOC	0-20	forest	29.95 N	16.1	1490	NH_4NO_3	150	2	0.2235	0.0025	
Tu et al. 2011b	Liujiang, Sichuan, China	DOC	0-20	forest	29.95 N	16.1	1490	NH_4NO_3	300	2	0.0642	0.0021	
Tu et al. 2011b	Liujiang, Sichuan, China	plant belowground part C		forest	29.95 N	16.1	1490	NH_4NO_3	50	2	-0.0087	0.0032	total
Tu et al. 2011b	Liujiang, Sichuan, China	plant belowground part C		forest	29.95 N	16.1	1490	NH_4NO_3	150	2	0.0091	0.0023	total
Tu et al. 2011b	Liujiang, Sichuan, China	plant belowground part C		forest	29.95 N	16.1	1490	NH_4NO_3	300	2	0.1932	0.0039	total
Liang and Balser 2012	San Francisco, USA	soil total C		grassland	37.41 N	13.9	607		70	9	0.3586	0.0115	
Wang et al. 2011	Siziwang Banner, Inner Mongolia, China	soil respiration		grassland	41.46 N	4	160	NH_4NO_3	100	1	-0.3064	0.0206	
Bowden et al. 2004	Harvard Forest, Massachusetts, USA	soil respiration		forest	42.3 N	3.5	112	NH_4NO_3	37	1	0.2298	0.009	
Bowden et al. 2004	Harvard Forest, Massachusetts, USA	soil respiration		forest	42.3 N	3.5	112	NH_4NO_3	120	1	0.2131	0.0093	
Bowden et al. 2004	Harvard Forest, Massachusetts, USA	soil respiration		forest	42.3 N	3.5	112	NH_4NO_3	37	1	-0.0028	0.0216	
Bowden et al. 2004	Harvard Forest, Massachusetts, USA	soil respiration		forest	42.3 N	3.5	112	NH_4NO_3	120	1	-0.1811	0.0175	
Koehler et al. 2009a	Gigante Peninsula, Panama	soil respiration		forest	9.06 N	27.4	2650	urea	125	3	-0.0821	0.0044	
Koehler et al. 2009a	Gigante Peninsula, Panama	soil respiration		forest	8.45 N	20.1	5532	urea	125	11	0.0182	0.0142	
Liu et al. 2010b	Guangdong, China	plant aboveground part C		forest	3.3 N	21.5	1900	NH_4NO_3	100	4	0.4991	0.0252	woody
Liu et al. 2010b	Guangdong, China	plant belowground part C		forest	3.3 N	21.5	1900	NH_4NO_3	100	4	0.3805	0.0071	woody
Liu et al. 2010b	Guangdong, China	soil respiration		forest	3.3 N	21.5	1900	NH_4NO_3	100	4	0.0227	0.1725	
Liu et al. 2010b	Guangdong, China	soil total C	0-20	forest	3.3 N	21.5	1900	NH_4NO_3	100	4	0.1613	0.0043	
Liu et al. 2010b	Guangdong, China	soil total C	20-40	forest	3.3 N	21.5	1900	NH_4NO_3	100	4	-0.0228	0.0049	
Liu et al. 2010b	Guangdong, China	soil total C	40-60	forest	3.3 N	21.5	1900	NH_4NO_3	100	4	-0.0825	0.0119	
Hasselquist et al. 2012	Umea, Northern Sweden	soil respiration		forest	64.1 N	1.2	520	NH_4NO_3	20	5	0.4145	0.0145	
Hasselquist et al. 2012	Umea, Northern Sweden	soil respiration		forest	64.1 N	1.2	520	NH_4NO_3	100	5	0.0425	0.0145	
Yan et al. 2010	Duolun County, Inner Mongolia, China	MBC	0-40	grassland	42.27 N	2.1	385	urea	280	2	-0.0294	0.0016	
Yan et al. 2010	Duolun County, Inner Mongolia, China	ANPP	0-40	grassland	42.27 N	2.1	385	urea	280	2	0.2371	0.0105	total
Yan et al. 2010	Duolun County, Inner Mongolia, China	BNPP	0-40	grassland	42.27 N	2.1	385	urea	280	2	0.0012	0.0175	total
Yan et al. 2010	Duolun County, Inner Mongolia, China	BNPP	0-10	grassland	42.27 N	2.1	385	urea	280	2	-0.1178	0.0123	total
Yan et al. 2010	Duolun County, Inner Mongolia, China	soil respiration		grassland	42.27 N	2.1	385	urea	280	2	-0.0842	0.0016	

Yan et al. 2010	Duolun County, Inner Mongolia, China	microbial respiration		grassland	42.27 N	2.1	385	urea	280	2	-0.1932	0.0016		
Chen et al. 2012b	Guangdong, China	soil TOC	0-20	forest	23.2 N	21.5	1750	NH_4NO_3	100	4	0.2696	0.1042		
Chen et al. 2012b	Guangdong, China	MBC	0-20	forest	23.2 N	21.5	1750	NH_4NO_3	100	4	-0.3207	0.0395		
Chen et al. 2012b	Guangdong, China	plant aboveground part C		forest	23.2 N	21.5	1750	NH_4NO_3	100	4	0.3844	0.0008		total
Chen et al. 2012b	Guangdong, China	plant belowground part C		forest	23.2 N	21.5	1750	NH_4NO_3	100	4	0.3131	0.0043		total
Chen et al. 2012b	Guangdong, China	soil respiration		forest	23.2 N	21.5	1750	NH_4NO_3	100	4	-0.1071	0.258		
Liu et al. 2011	Guangdong, China	soil TOC	0-20	forest	23.3 N	21.5	1750	NH_4NO_3	100	3	0.1549	0.0068		
Liu et al. 2011	Guangdong, China	soil TOC	20-40	forest	23.2 N	21.5	1750	NH_4NO_3	100	3	-0.0023	0.0073		
Liu et al. 2011	Guangdong, China	soil TOC	40-60	forest	23.2 N	21.5	1750	NH_4NO_3	100	3	0.253	0.02		
Liu et al. 2011	Guangdong, China	soil TIC	0-20	forest	23.2 N	21.5	1750	NH_4NO_3	100	3	0.0572	1.8496		
Liu et al. 2011	Guangdong, China	soil TIC	20-40	forest	23.2 N	21.5	1750	NH_4NO_3	100	3	-0.1625	2.0937		
Liu et al. 2011	Guangdong, China	soil TIC	40-60	forest	23.2 N	21.5	1750	NH_4NO_3	100	3	-0.3677	3.1723		
Liu et al. 2011	Guangdong, China	soil respiration		forest	23.3 N	21.5	1750	NH_4NO_3	100	3	0.0227	0.1725		
Song et al. 2013a	Sanjiang Plain, Heilongjiang, China	litterfall		wetland	47.35 N	2.5	556	NH_4NO_3	60	5	0.2542	0.0066		
Song et al. 2013a	Sanjiang Plain, Heilongjiang, China	litterfall		wetland	47.35 N	2.5	556	NH_4NO_3	120	5	0.3853	0.0078		
Song et al. 2013a	Sanjiang Plain, Heilongjiang, China	litterfall		wetland	47.35 N	2.5	556	NH_4NO_3	240	5	0.919	0.0204		
Song et al. 2013a	Sanjiang Plain, Heilongjiang, China	soil TOC	0-15	wetland	47.35 N	2.5	556	NH_4NO_3	60	5	0.1353	0.0004		
Song et al. 2013a	Sanjiang Plain, Heilongjiang, China	soil TOC	0-15	wetland	47.35 N	2.5	556	NH_4NO_3	120	5	0.1032	0.0003		
Song et al. 2013a	Sanjiang Plain, Heilongjiang, China	soil TOC	0-15	wetland	47.35 N	2.5	556	NH_4NO_3	240	5	-0.2829	0.0006		
Song et al. 2013a	Sanjiang Plain, Heilongjiang, China	MBC	0-15	wetland	47.35 N	2.5	556	NH_4NO_3	60	5	0.1648	0.0006		
Song et al. 2013a	Sanjiang Plain, Heilongjiang, China	MBC	0-15	wetland	47.35 N	2.5	556	NH_4NO_3	120	5	-0.0075	0.0048		
Song et al. 2013a	Sanjiang Plain, Heilongjiang, China	MBC	0-15	wetland	47.35 N	2.5	556	NH_4NO_3	240	5	-0.4911	0.0123		
Song et al. 2011	Sanjiang Plain, Heilongjiang, China	litter decomposition rate		wetland	47.35 N	2.5	556	NH_4NO_3	240	1	0.1398	0.0235	*Calamagrostis*	herbaceous
Song et al. 2013b	Sanjiang Plain, Heilongjiang, China	plant aboveground part C		wetland	47.36 N	1.9	600	NH_4NO_3	60	4	0.4499	0.0299		total
Song et al. 2013b	Sanjiang Plain, Heilongjiang, China	plant aboveground part C		wetland	47.36 N	1.9	600	NH_4NO_3	120	4	0.5531	0.0191		total

Song et al. 2013b	Sanjiang Plain, Heilongjiang, China	plant aboveground part C		wetland	47.36 N	1.9	600	NH_4NO_3	240	4	0.5661	0.0349	total
Song et al. 2013b	Sanjiang Plain, Heilongjiang, China	soil respiration		wetland	47.36 N	1.9	600	NH_4NO_3	60	4	0.0933	0.0052	
Song et al. 2013b	Sanjiang Plain, Heilongjiang, China	soil respiration		wetland	47.36 N	1.9	600	NH_4NO_3	120	4	0.2484	0.0055	
Song et al. 2013b	Sanjiang Plain, Heilongjiang, China	soil respiration		wetland	47.36 N	1.9	600	NH_4NO_3	240	4	0.5255	0.0072	
Ket et al. 2011	Altamaha River, Georgia, USA	plant aboveground part C		wetland	31.33 N	20.3	1261	urea	500	5	1.0044	0.0794	herbaceous
Keeler et al. 2009	Minnesota, USA	soil total C	0-20	grassland	45.4 N	6.7	801	NH_4NO_3	100	5	-0.0031	0.0524	
Keeler et al. 2009	Minnesota, USA	soil total C	0-20	grassland	45.4 N	6.7	801	NH_4NO_3	100	5	-0.1523	0.0184	
Keeler et al. 2009	Minnesota, USA	soil total C	0-20	forest	45.4 N	6.7	801	NH_4NO_3	100	5	0.0415	0.0049	
Keeler et al. 2009	Minnesota, USA	soil total C	0-20	forest	45.4 N	6.7	801	NH_4NO_3	100	5	0.0238	0.0065	
Keeler et al. 2009	Minnesota, USA	soil total C	0-20	forest	45.4 N	6.7	801	NH_4NO_3	100	5	0.0891	0.0431	
Keeler et al. 2009	Minnesota, USA	soil total C	0-20	forest	45.4 N	6.7	801	NH_4NO_3	100	5	-0.0065	0.0211	
Keeler et al. 2009	Minnesota, USA	soil total C	0-20	forest	45.4 N	6.7	801	NH_4NO_3	100	5	0.0359	0.0204	
Keeler et al. 2009	Minnesota, USA	soil total C	0-20	forest	45.4 N	6.7	801	NH_4NO_3	100	5	0.5552	0.0979	
Keeler et al. 2009	Minnesota, USA	MBC	0-20	grassland	45.4 N	6.7	801	NH_4NO_3	100	5	-0.2316	0.0185	
Keeler et al. 2009	Minnesota, USA	MBC	0-20	grassland	45.4 N	6.7	801	NH_4NO_3	100	5	-0.4154	0.0124	
Keeler et al. 2009	Minnesota, USA	MBC	0-20	forest	45.4 N	6.7	801	NH_4NO_3	100	5	0.1483	0.0117	
Keeler et al. 2009	Minnesota, USA	MBC	0-20	forest	45.4 N	6.7	801	NH_4NO_3	100	5	-0.1344	0.007	
Keeler et al. 2009	Minnesota, USA	MBC	0-20	forest	45.4 N	6.7	801	NH_4NO_3	100	5	-0.3025	0.019	
Keeler et al. 2009	Minnesota, USA	MBC	0-20	forest	45.4 N	6.7	801	NH_4NO_3	100	5	-0.0044	0.0127	
Keeler et al. 2009	Minnesota, USA	MBC	0-20	forest	45.4 N	6.7	801	NH_4NO_3	100	5	-0.1424	0.0301	
Keeler et al. 2009	Minnesota, USA	MBC	0-20	forest	45.4 N	6.7	801	NH_4NO_3	100	5	-0.0494	0.0141	
Keeler et al. 2009	Minnesota, USA	litter decomposition rate		grassland	45.4 N	6.7	801	NH_4NO_3	100	5	0.1882	0.036	
Keeler et al. 2009	Minnesota, USA	litter decomposition rate		grassland	45.4 N	6.7	801	NH_4NO_3	100	5	0.0101	0.0029	
Keeler et al. 2009	Minnesota, USA	litter decomposition rate		forest	45.4 N	6.7	801	NH_4NO_3	100	5	0.1822	0.0129	
Keeler et al. 2009	Minnesota, USA	litter decomposition rate		forest	45.4 N	6.7	801	NH_4NO_3	100	5	-0.0357	0.0126	
Keeler et al. 2009	Minnesota, USA	litter decomposition rate		forest	45.4 N	6.7	801	NH_4NO_3	100	5	0.0073	0.0287	

Keeler et al. 2009	Minnesota, USA	litter decomposition rate		forest	45.4 N	6.7	801	NH_4NO_3	100	5	-0.1503	0.0086	
Keeler et al. 2009	Minnesota, USA	litter decomposition rate		forest	45.4 N	6.7	801	NH_4NO_3	100	5	-0.0217	0.0149	
Keeler et al. 2009	Minnesota, USA	litter decomposition rate		forest	45.4 N	6.7	801	NH_4NO_3	100	5	0.0451	0.0226	
Keeler et al. 2009	Minnesota, USA	soil respiration		grassland	45.4 N	6.7	801	NH_4NO_3	100	5	-0.0765	0.001	
Keeler et al. 2009	Minnesota, USA	soil respiration		grassland	45.4 N	6.7	801	NH_4NO_3	100	5	0.0116	0.0013	
Keeler et al. 2009	Minnesota, USA	soil respiration		forest	45.4 N	6.7	801	NH_4NO_3	100	5	-0.11	0.0015	
Keeler et al. 2009	Minnesota, USA	soil respiration		forest	45.4 N	6.7	801	NH_4NO_3	100	5	0	0.0011	
Keeler et al. 2009	Minnesota, USA	soil respiration		forest	45.4 N	6.7	801	NH_4NO_3	100	5	0	0.0003	
Keeler et al. 2009	Minnesota, USA	soil respiration		forest	45.4 N	6.7	801	NH_4NO_3	100	5	0.021	0.0006	
Han et al. 2012	Duolun County, Inner Mongolia, China	soil respiration		grassland	42.02 N	2.1	383	NH_4NO_3	100	3	0.1076	0.0067	
Han et al. 2012	Duolun County, Inner Mongolia, China	soil respiration		grassland	42.02 N	2.1	383	NH_4NO_3	100	3	-0.0704	0.0042	
Han et al. 2012	Duolun County, Inner Mongolia, China	soil respiration		grassland	42.02 N	2.1	383	NH_4NO_3	100	3	-0.0084	0.0145	
Li et al. 2010	Jilin, China	litterfall		forest	42.24 N	2.7	872	NH_4NO_3	25	2	0.2235	0.0079	
Li et al. 2010	Jilin, China	litterfall		forest	42.24 N	2.7	872	NH_4NO_3	50	2	0.2981	0.009	
Li et al. 2010	Jilin, China	litterfall		forest	42.24 N	2.7	872	NH_4NO_3	25	2	0.1393	0.0017	
Li et al. 2010	Jilin, China	litterfall		forest	42.24 N	2.7	872	NH_4NO_3	50	2	0.0394	0.0016	
Li et al. 2010	Jilin, China	litterfall		forest	42.24 N	2.7	872	NH_4NO_3	25	2	0.1584	0.0246	
Li et al. 2010	Jilin, China	litterfall		forest	42.24 N	2.7	872	NH_4NO_3	50	2	0.2612	0.019	
Li et al. 2010	Jilin, China	litterfall		forest	42.24 N	2.7	872	NH_4NO_3	25	2	0.2587	0.0261	
Li et al. 2010	Jilin, China	litterfall		forest	42.24 N	2.7	872	NH_4NO_3	50	2	0.1823	0.0184	
Li et al. 2010	Jilin, China	litterfall		forest	42.24 N	2.7	872	NH_4NO_3	25	2	0.0211	0.0025	
Li et al. 2010	Jilin, China	litterfall		forest	42.24 N	2.7	872	NH_4NO_3	50	2	0.2498	0.0023	
Li et al. 2010	Jilin, China	litterfall		forest	42.24 N	2.7	872	NH_4NO_3	25	2	0.0426	0.0042	
Li et al. 2010	Jilin, China	litterfall		forest	42.24 N	2.7	872	NH_4NO_3	50	2	0.0719	0.0041	
Huang et al. 2011	Central North Island, New Zealand	soil total C	0-5	forest	38.14 S	11.5	1764	urea	250	15	-0.127	0.0051	
Huang et al. 2011	Central North Island, New Zealand	plant aboveground part C		forest	38.14 S	11.5	1764	urea	250	15	0.0537	0.0009	woody

Huang et al. 2011	Central North Island, New Zealand	litterfall		forest	38.14 S	11.5	1764	urea	250	15	0.1008	0.0027	
Huang et al. 2011	Central North Island, New Zealand	soil total C	O-horizon	forest	38.14 S	11.5	1764	urea	250	15	0.037	0.0097	
Cusack et al. 2011	Puerto Rico	plant aboveground part C		forest	18.3 N	27.5	3500	NH_4NO_3	50	3	0.1846	0.1634	total
Cusack et al. 2011	Puerto Rico	plant aboveground part C		forest	18.3 N	27.5	3500	NH_4NO_3	50	3	-0.2653	0.0001	total
Cusack et al. 2011	Puerto Rico	soil total C	0-10	forest	18.3 N	27.5	3500	NH_4NO_3	50	3	0.2076	0.0096	
Cusack et al. 2011	Puerto Rico	soil total C	10-20	forest	18.3 N	27.5	3500	NH_4NO_3	50	3	0.2007	0.0092	
Cusack et al. 2011	Puerto Rico	soil total C	20-30	forest	18.3 N	27.5	3500	NH_4NO_3	50	3	0.1911	0.0047	
Cusack et al. 2011	Puerto Rico	soil total C	30-40	forest	18.3 N	27.5	3500	NH_4NO_3	50	3	0.1942	0.0362	
Cusack et al. 2011	Puerto Rico	soil total C	0-10	forest	18.3 N	27.5	3500	NH_4NO_3	50	3	0.2877	0.064	
Cusack et al. 2011	Puerto Rico	soil total C	10-20	forest	18.3 N	27.5	3500	NH_4NO_3	50	3	0.2744	0.0849	
Cusack et al. 2011	Puerto Rico	soil total C	20-30	forest	18.3 N	27.5	3500	NH_4NO_3	50	3	0.1942	0.0916	
Cusack et al. 2011	Puerto Rico	soil total C	30-40	forest	18.3 N	27.5	3500	NH_4NO_3	50	3	0.0513	0.1149	
Cusack et al. 2011	Puerto Rico	soil respiration		forest	18.3 N	27.5	3500	NH_4NO_3	50	3	-0.2136	0.0344	
Cusack et al. 2011	Puerto Rico	soil respiration		forest	18.3 N	27.5	3500	NH_4NO_3	50	3	-0.3824	0.0014	
Cusack et al. 2011	Puerto Rico	litterfall		forest	18.3 N	27.5	3500	NH_4NO_3	50	3	-0.0304	0.0004	
Cusack et al. 2011	Puerto Rico	litterfall		forest	18.3 N	27.5	3500	NH_4NO_3	50	3	-0.0038	0.0001	
Lu et al. 2013	Guangdong, China	DOC	0-20	forest	23.1 N	21	1927	NH_4NO_3	50	7	-0.1712	0.0116	
Lu et al. 2013	Guangdong, China	DOC	0-20	forest	23.1 N	21	1927	NH_4NO_3	100	7	-0.3373	0.0112	
Lu et al. 2013	Guangdong, China	DOC	0-20	forest	23.1 N	21	1927	NH_4NO_3	150	7	-0.4697	0.0092	
Lu et al. 2013	Guangdong, China	soil total C	0-20	forest	23.1 N	21	1927	NH_4NO_3	50	7	0.0995	0.0019	
Lu et al. 2013	Guangdong, China	soil total C	0-20	forest	23.1 N	21	1927	NH_4NO_3	100	7	0.1261	0.0007	
Lu et al. 2013	Guangdong, China	soil total C	0-20	forest	23.1 N	21	1927	NH_4NO_3	150	7	0.1586	0.0035	
Frost et al. 2009	Altamaha River, Georgia, USA	plant aboveground part C		wetland	31.33 N	20.3	1261	NH_4^+	500	1	0.2198	0.0799	total
Tu et al. 2013	Liujiang, Sichuan, China	soil respiration		forest	29.42 N	16.1	1490	NH_4NO_3	50	2	0.2284	0.0015	
Tu et al. 2013	Liujiang, Sichuan, China	soil respiration		forest	29.42 N	16.1	1490	NH_4NO_3	150	2	0.2424	0.0011	
Tu et al. 2013	Liujiang, Sichuan, China	soil respiration		forest	29.42 N	16.1	1490	NH_4NO_3	300	2	0.4143	0.0007	

Tu et al. 2013	Liujiang, Sichuan, China	microbial respiration		forest	29.42 N	16.1	1490	NH_4NO_3	50	2	0.3302	0.0058		
Tu et al. 2013	Liujiang, Sichuan, China	microbial respiration		forest	29.42 N	16.1	1490	NH_4NO_3	150	2	0.2657	0.0063		
Tu et al. 2013	Liujiang, Sichuan, China	microbial respiration		forest	29.42 N	16.1	1490	NH_4NO_3	300	2	0.361	0.0028		
Lovett et al. 2013	New York, USA	litter decomposition rate		forest	42.07 N	4.3	153	NH_4NO_3	50	6	-0.1437	0.0092	*Fagus*	woody
Lovett et al. 2013	New York, USA	litter decomposition rate		forest	42.07 N	4.3	153	NH_4NO_3	50	6	-0.0847	0.066	*Tsuga*	woody
Lovett et al. 2013	New York, USA	litter decomposition rate		forest	42.07 N	4.3	153	NH_4NO_3	50	6	-0.0225	0.0157	*Acer*	woody
Lovett et al. 2013	New York, USA	litter decomposition rate		forest	42.07 N	4.3	153	NH_4NO_3	50	6	0.0047	0.0094	*Quercus*	woody
Lovett et al. 2013	New York, USA	litter decomposition rate		forest	42.07 N	4.3	153	NH_4NO_3	50	6	-0.1259	0.0105	*Betula*	woody
Lovett et al. 2013	New York, USA	ANPP		forest	42.07 N	4.3	153	NH_4NO_3	50	6	-0.1823	0.0108		woody
Lovett et al. 2013	New York, USA	ANPP		forest	42.07 N	4.3	153	NH_4NO_3	50	6	-0.2157	0.0173		woody
Lovett et al. 2013	New York, USA	ANPP		forest	42.07 N	4.3	153	NH_4NO_3	50	6	0.0413	0.0428		woody
Lovett et al. 2013	New York, USA	ANPP		forest	42.07 N	4.3	153	NH_4NO_3	50	6	0.0953	0.0074		woody
Lovett et al. 2013	New York, USA	ANPP		forest	42.07 N	4.3	153	NH_4NO_3	50	6	-0.1919	0.0126		woody
Hu et al. 2010	Heilongjiang, China	soil TOC	0-10	forest	45.21 N	2.8	723	NH_4NO_3	100	6	0.023	0.0103		
Hu et al. 2010	Heilongjiang, China	soil TOC	10-20	forest	45.21 N	2.8	723	NH_4NO_3	100	6	-0.1059	0.0169		
Hu et al. 2010	Heilongjiang, China	MBC	0-10	forest	45.21 N	2.8	723	NH_4NO_3	100	6	-0.2724	0.0075		
Hu et al. 2010	Heilongjiang, China	MBC	10-20	forest	45.21 N	2.8	723	NH_4NO_3	100	6	-0.2231	0.0693		
Hu et al. 2010	Heilongjiang, China	soil respiration	0-10	forest	45.21 N	2.8	723	NH_4NO_3	100	6	-0.1119	0.01		
Hu et al. 2010	Heilongjiang, China	soil respiration	10-20	forest	45.21 N	2.8	723	NH_4NO_3	100	6	-0.1639	0.0529		
Hu et al. 2010	Heilongjiang, China	DOC	0-10	forest	45.21 N	2.8	723	NH_4NO_3	100	6	0.1121	0.0124		
Hu et al. 2010	Heilongjiang, China	DOC	10-20	forest	45.21 N	2.8	723	NH_4NO_3	100	6	-0.153	0.0057		
Zhang et al. 2013b	Sanjiang Plain, Heilongjiang, China	GEP		wetland	47.35 N	1.9	600	NH_4NO_3	240	2	0.6998	0.0052		
Zhang et al. 2013b	Sanjiang Plain, Heilongjiang, China	plant aboveground part C		wetland	47.35 N	1.9	600	NH_4NO_3	240	2	1.5871	0.0796		total

Zhang et al. 2013b	Sanjiang Plain, Heilongjiang, China	ER		wetland	47.35 N	1.9	600	NH_4NO_3	240	2	0.8976	0.0069	
Cusack et al. 2010	Puerto Rico	soil total C	0-10	forest	18.3 N	23	3537	NH_4NO_3	50	1	0.1268	0.0088	
Cusack et al. 2010	Puerto Rico	soil total C	0-10	forest	18.3 N	21	3537	NH_4NO_3	50	1	0.6493	0.0795	
Cusack et al. 2010	Puerto Rico	DOC	0-10	forest	18.3 N	23	3537	NH_4NO_3	50	1	0.1823	0.0469	
Cusack et al. 2010	Puerto Rico	DOC	0-10	forest	18.3 N	21	3537	NH_4NO_3	50	1	0.1872	0.0353	
Cusack et al. 2010	Puerto Rico	plant belowground part C		forest	18.3 N	23	3537	NH_4NO_3	50	1	-0.8575	0.4084	total
Cusack et al. 2010	Puerto Rico	plant belowground part C		forest	18.3 N	21	3537	NH_4NO_3	50	1	-0.3659	0.1802	total
Cusack et al. 2010	Puerto Rico	soil respiration		forest	18.3 N	23	3537	NH_4NO_3	50	1	-0.0171	0.0021	
Cusack et al. 2010	Puerto Rico	soil respiration		forest	18.3 N	21	3537	NH_4NO_3	50	1	0.1501	0.0047	
Song et al. 2013d	Sanjiang Plain, Heilongjiang, China	MBC	0-15	wetland	47.35 N	2.5	600	NH_4NO_3	240	1	0.1524	0.0339	
Song et al. 2013d	Sanjiang Plain, Heilongjiang, China	MBC	15-30	wetland	47.35 N	2.5	600	NH_4NO_3	240	1	0.312	0.1476	
Song et al. 2013d	Sanjiang Plain, Heilongjiang, China	DOC	0-15	wetland	47.35 N	2.5	600	NH_4NO_3	240	1	0.0705	0.0351	
Song et al. 2013d	Sanjiang Plain, Heilongjiang, China	DOC	15-30	wetland	47.35 N	2.5	600	NH_4NO_3	240	1	0.1005	0.391	
Song et al. 2013d	Sanjiang Plain, Heilongjiang, China	soil TOC	0-15	wetland	47.35 N	2.5	600	NH_4NO_3	240	1	0.1363	0.265	
Song et al. 2013d	Sanjiang Plain, Heilongjiang, China	soil TOC	15-30	wetland	47.35 N	2.5	600	NH_4NO_3	240	1	0.4964	0.6667	
Song et al. 2013c	Sanjiang Plain, Heilongjiang, China	MBC	0-15	wetland	47.35 N	2.5	600	NH_4NO_3	120	1	0.8317	0.0013	
Song et al. 2013c	Sanjiang Plain, Heilongjiang, China	MBC	0-15	wetland	47.35 N	2.5	600	NH_4NO_3	240	1	0.1618	0.0305	
Song et al. 2013c	Sanjiang Plain, Heilongjiang, China	MBC	15-30	wetland	47.35 N	2.5	600	NH_4NO_3	120	1	0.9765	0.0327	
Song et al. 2013c	Sanjiang Plain, Heilongjiang, China	MBC	15-30	wetland	47.35 N	2.5	600	NH_4NO_3	240	1	0.3216	0.1415	
Song et al. 2013c	Sanjiang Plain, Heilongjiang, China	DOC	0-15	wetland	47.35 N	2.5	600	NH_4NO_3	120	1	-0.1703	0.1252	
Song et al. 2013c	Sanjiang Plain, Heilongjiang, China	DOC	0-15	wetland	47.35 N	2.5	600	NH_4NO_3	240	1	0.0622	0.0755	
Song et al. 2013c	Sanjiang Plain, Heilongjiang, China	DOC	15-30	wetland	47.35 N	2.5	600	NH_4NO_3	120	1	-0.4274	0.0674	
Song et al. 2013c	Sanjiang Plain, Heilongjiang, China	DOC	15-30	wetland	47.35 N	2.5	600	NH_4NO_3	240	1	0.0904	0.0543	
Iversen et al. 2010	Gogebic County, Michigan, USA	plant aboveground part C		wetland	46 N	6.3	734	urea	60	5	0.9569	0.0037	total
Iversen et al. 2010	Gogebic County, Michigan, USA	plant aboveground part C		wetland	46 N	6.3	734	urea	60	5	0.2866	0.0524	total
Iversen et al. 2010	Gogebic County, Michigan, USA	plant aboveground part C		wetland	46 N	6.3	734	urea	60	5	0.1397	0.0142	total

Iversen et al. 2010	Gogebic County, Michigan, USA	ANPP		wetland	46 N	6.3	734	urea	60	5	0.6011	0.0023	total
Iversen et al. 2010	Gogebic County, Michigan, USA	ANPP		wetland	46 N	6.3	734	urea	60	5	0.6874	0.0335	total
Iversen et al. 2010	Gogebic County, Michigan, USA	ANPP		wetland	46 N	6.3	734	urea	60	5	0.0953	0.0138	total
Deng et al. 2010	Guangdong, China	soil respiration		forest	23.2 N	21.5	1675	NH_4NO_3	100	1	0.0085	0	
Deng et al. 2010	Guangdong, China	plant aboveground part C		forest	23.2 N	21.5	1675	NH_4NO_3	100	1	0.2693	0.0094	total
Deng et al. 2010	Guangdong, China	plant belowground part C		forest	23.2 N	21.5	1675	NH_4NO_3	100	1	0.2436	0.0037	total
Deng et al. 2010	Guangdong, China	soil TOC		forest	23.2 N	21.5	1675	NH_4NO_3	100	1	0.0947	0.0038	
Churchland et al. 2010	Daring Lake, Northwest Territories, Canada	MBC	O-horizon	tundra	64.52 N	-9	210	NH_4NO_3	100	2	-0.3132	0.0208	
Sardans et al.2006	Catalonia, Spain	plant aboveground part C		shrubland	41.37 N	14	517	NH_4NO_3	500	1	-0.2477	0.0898	total
Baez et al. 2007	Central New Mexico, USA	plant aboveground part C		grassland	34.20 N	13.2	255	NH_4NO_3	20	1	-0.1224	0.0002	herbaceous
Baez et al. 2007	Central New Mexico, USA	plant aboveground part C		grassland	34.20 N	13.2	255	NH_4NO_3	20	1	0.6455	0.0002	herbaceous
Dag-Inge 2004	Central Norway	plant aboveground part C		wetland	62.40 N	0.6	600	NH_4NO_3	120	2	2.1361	0.4877	woody
Dag-Inge 2004	Central Norway	plant aboveground part C		wetland	62.40 N	0.6	600	NH_4NO_3	120	2	1.5869	1.4316	woody
Dag-Inge 2004	Central Norway	plant aboveground part C		wetland	62.40 N	0.6	600	NH_4NO_3	120	2	-1.947	0.7075	woody
Dag-Inge 2004	Central Norway	plant aboveground part C		wetland	62.40 N	0.6	600	NH_4NO_3	120	2	0.6945	0.1616	herbaceous
Dag-Inge 2004	Central Norway	plant aboveground part C		wetland	62.40 N	0.6	600	NH_4NO_3	120	2	1.0644	0.8489	herbaceous
Dag-Inge 2004	Central Norway	plant aboveground part C		wetland	62.40 N	0.6	600	NH_4NO_3	120	2	-0.0547	0.0498	herbaceous
Dag-Inge 2004	Central Norway	plant aboveground part C		wetland	62.40 N	0.6	600	NH_4NO_3	120	2	-0.0273	0.0349	herbaceous
Dag-Inge 2004	Central Norway	plant aboveground part C		wetland	62.40 N	0.6	600	NH_4NO_3	120	2	-0.0358	0.0247	herbaceous
Dag-Inge 2004	Central Norway	plant aboveground part C		wetland	62.40 N	0.6	600	NH_4NO_3	120	2	-0.0741	0.0082	herbaceous
Bonanomi et al. 2006	Central Italy	plant aboveground part C		grassland	43.2 N	12.9	945	urea	35	3	0.6212	0.0324	herbaceous
Turner & Knapp 1996	Northeast Kansas, USA	ANPP		grassland	39.05 N	4.7	890	NH_4NO_3	100	1	0.3172	0.058	herbaceous
Turner & Knapp 1996	Northeast Kansas, USA	ANPP		grassland	39.05 N	4.7	890	NH_4NO_3	100	1	-0.0118	0.0204	herbaceous
Turner & Knapp 1996	Northeast Kansas, USA	ANPP		grassland	39.05 N	4.7	890	NH_4NO_3	100	1	0.2465	0.1288	herbaceous
Turner & Knapp 1996	Northeast Kansas, USA	ANPP		grassland	39.05 N	4.7	890	NH_4NO_3	100	1	0.5474	0.0953	herbaceous

Bowman et al. 1993	Niwot Ridge, UAS	plant belowground part C	tundra	39.24 N	-3	900	urea	250	2	-0.1158	0.0825		
Bowman et al. 1993	Niwot Ridge, UAS	plant belowground part C	tundra	39.24 N	-3	900	urea	250	2	0.1914	0.0344		
Aerts et al. 2006	Northern Sweden	litter decomposition rate	wetland	68.2 N	0.5	320	NH_4	100	3	0.1301	0.0035	*Empetrum nigrum*	woody
Aerts et al. 2006	Northern Sweden	litter decomposition rate	wetland	68.2 N	0.5	320	NH_4	100	3	0	0.0118	*Eriophorum vaginatum*	herbaceous
Aerts et al. 2006	Northern Sweden	litter decomposition rate	wetland	68.2 N	0.5	320	NH_4	100	3	-0.1226	0.0088	*Betula nana*	woody
Aerts et al. 2006	Northern Sweden	litter decomposition rate	wetland	68.2 N	0.5	320	NH_4	100	3	-0.1613	0.0016	*Rubus chamaemorus*	herbaceous
Barger et al. 2003	Caracas, Venezuela	plant aboveground part C	grassland	10.5 N	17.9	1063	urea	200	2	1.2841	0.1369		herbaceous
Barger et al. 2003	Caracas, Venezuela	plant belowground part C	grassland	10.5 N	17.9	1063	urea	200	2	0.9719	0.2004		herbaceous
Barger et al. 2003	Caracas, Venezuela	plant aboveground part C	grassland	10.5 N	17.9	1063	urea	200	2	1.411	0.2771		herbaceous
Barger et al. 2003	Caracas, Venezuela	plant belowground part C	grassland	10.5 N	17.9	1063	urea	200	2	1.2072	0.342		herbaceous
Gunnarsson & Rydin 2000	Akhultmyren, Sweden	ANPP	wetland	57.06 N	7.8	789	NH_4NO_3	10	2	-0.3069	0.5609	*Sphagnum*	moss
Gunnarsson & Rydin 2000	Akhultmyren, Sweden	ANPP	wetland	57.06 N	7.8	789	NH_4NO_3	30	2	-0.0704	0.2761	*Sphagnum*	moss
Gunnarsson & Rydin 2000	Akhultmyren, Sweden	ANPP	wetland	57.06 N	7.8	789	NH_4NO_3	50	2	-0.6339	0.4755	*Sphagnum*	moss
Gunnarsson & Rydin 2000	Akhultmyren, Sweden	ANPP	wetland	57.06 N	7.8	789	NH_4NO_3	100	2	-0.6903	1.2791	*Sphagnum*	moss
Gunnarsson & Rydin 2000	Akhultmyren, Sweden	ANPP	wetland	57.06 N	7.8	789	NH_4NO_3	10	2	-0.0076	2.3387	*Sphagnum*	moss
Gunnarsson & Rydin 2000	Akhultmyren, Sweden	ANPP	wetland	57.06 N	7.8	789	NH_4NO_3	30	2	-0.2864	0.3818	*Sphagnum*	moss
Gunnarsson & Rydin 2000	Akhultmyren, Sweden	ANPP	wetland	57.06 N	7.8	789	NH_4NO_3	50	2	-0.2256	1.4668	*Sphagnum*	moss
Gunnarsson & Rydin 2000	Akhultmyren, Sweden	ANPP	wetland	57.06 N	7.8	789	NH_4NO_3	100	2	-0.0147	0.4155	*Sphagnum*	moss
Gunnarsson & Rydin 2000	Kopparasmyren, Sweden	ANPP	wetland	57.07 N	7.8	789	NH_4NO_3	10	2	0.0917	0.4553	*Sphagnum*	moss
Gunnarsson & Rydin 2000	Kopparasmyren, Sweden	ANPP	wetland	57.07 N	7.8	789	NH_4NO_3	30	2	-0.0384	0.4509	*Sphagnum*	moss
Gunnarsson & Rydin 2000	Kopparasmyren, Sweden	ANPP	wetland	57.07 N	7.8	789	NH_4NO_3	50	2	-0.188	0.5656	*Sphagnum*	moss
Gunnarsson & Rydin 2000	Kopparasmyren, Sweden	ANPP	wetland	57.07 N	7.8	789	NH_4NO_3	100	2	-0.3104	0.6202	*Sphagnum*	moss
Gunnarsson & Rydin 2000	Kopparasmyren, Sweden	ANPP	wetland	57.07 N	7.8	789	NH_4NO_3	10	2	-0.098	0.3103	*Sphagnum*	moss

Gunnarsson & Rydin 2000	Kopparasmyren, Sweden	ANPP		wetland	57.07 N	7.8	789	NH_4NO_3	30	2	-0.0401	0.7577	*Sphagnum*	moss
Gunnarsson & Rydin 2000	Kopparasmyren, Sweden	ANPP		wetland	57.07 N	7.8	789	NH_4NO_3	50	2	-0.2226	0.5287	*Sphagnum*	moss
Gunnarsson & Rydin 2000	Kopparasmyren, Sweden	ANPP		wetland	57.07 N	7.8	789	NH_4NO_3	100	2	-0.4889	0.5463	*Sphagnum*	moss
Gunnarsson & Rydin 2000	Luttumyren, Sweden	ANPP		wetland	61.02 N	6.5	814	NH_4NO_3	10	2	0.0002	0.1449	*Sphagnum*	moss
Gunnarsson & Rydin 2000	Luttumyren, Sweden	ANPP		wetland	61.02 N	6.5	814	NH_4NO_3	30	2	-0.108	0.0509	*Sphagnum*	moss
Gunnarsson & Rydin 2000	Luttumyren, Sweden	ANPP		wetland	61.02 N	6.5	814	NH_4NO_3	50	2	-0.0777	0.0627	*Sphagnum*	moss
Green and Galatowitsch 2002	Carver County, Minnesota, USA	plant aboveground part C		wetland	44.51 N	5.5	72	NO_3	120	1	0.3046	0.0067		total
Green and Galatowitsch 2002	Carver County, Minnesota, USA	plant aboveground part C		wetland	44.51 N	5.5	72	NO_3	480	1	0.4533	0.0053		total
Green and Galatowitsch 2002	Carver County, Minnesota, USA	plant belowground part C		wetland	44.51 N	5.5	72	NO_3	120	1	-0.1318	0.0212		total
Green and Galatowitsch 2002	Carver County, Minnesota, USA	plant belowground part C		wetland	44.51 N	5.5	72	NO_3	480	1	-0.0938	0.0242		total
Vose et al. 1995	Placerville, California, USA	soil respiration		forest	39 N	18	1000	NH_4	100	3	0.8327	0.0939		
Vose et al. 1995	Placerville, California, USA	soil respiration		forest	39 N	18	1000	NH_4	200	3	0.412	0.1251		
Vose et al. 1995	Placerville, California, USA	plant belowground part C		forest	39 N	18	1000	NH_4	100	3	0.3724	0.0511		total
Vose et al. 1995	Placerville, California, USA	plant belowground part C		forest	39 N	18	1000	NH_4	200	3	0.3663	0.0844		total
Hoosbeek et al. 2002	Salmisuo, Ilomantsi, eastern Finland	microbial respiration	O-horizon	wetland	62.47 N	2	600	NH_4NO_3	30	3	-0.1452	0.1782		
Hoosbeek et al. 2002	Kopparasmyren, southern Sweden	microbial respiration	O-horizon	wetland	57.8 N	7	800	NH_4NO_3	30	3	-0.1769	0.1728		
Hoosbeek et al. 2002	Kopparasmyren, southern Sweden	microbial respiration	O-horizon	wetland	57.8 N	7	800	NH_4NO_3	30	3	-0.1495	0.4545		
Hoosbeek et al. 2002	Kopparasmyren, southern Sweden	microbial respiration	O-horizon	wetland	57.8 N	7	800	NH_4NO_3	30	3	0.4055	1.0068		
Hoosbeek et al. 2002	Wageningen	microbial respiration	O-horizon	wetland	51.99 N	9.5	805	NH_4NO_3	50	3	0	0.0605		
Hoosbeek et al. 2002	La Chaux-des-Breuleux, Swiss Jura	microbial respiration	O-horizon	wetland	47.13 N	5	1390	NH_4NO_3	30	3	0	0.1925		

Hoosbeek et al. 2002	La Chaux-des-Breuleux, Swiss Jura	microbial respiration	O-horizon	wetland	47.13 N	5	1390	NH_4NO_3	30	3	0	0.7537	
Hoosbeek et al. 2002	La Chaux-des-Breuleux, Swiss Jura	microbial respiration	O-horizon	wetland	47.13 N	5	1390	NH_4NO_3	30	3	0.1823	0.2602	
van der Hoek et al. 2004	Bennekomse Meent, the Netherland	plant aboveground part C		wetland	52.01 N	9.5	833	NH_4NO_3	200	1	0.288	0.0474	total
van der Hoek et al. 2004	Bennekomse Meent, the Netherland	plant aboveground part C		wetland	52.01 N	9.5	833	NH_4NO_3	200	1	-0.0559	0.095	total
van der Hoek et al. 2004	Bennekomse Meent, the Netherland	litter C		wetland	52.01 N	9.5	833	NH_4NO_3	200	1	0.1581	0.0612	
van der Hoek et al. 2004	Bennekomse Meent, the Netherland	litter C		wetland	52.01 N	9.5	833	NH_4NO_3	200	1	0	0.1115	
Fang et al. 2012	Dinghu Mountain, Guangdong, China	MBC	0-5	forest	23.09 N	20.9	1956	NH_4NO_3	150	8	0.0809	0.0354	
Fang et al. 2012	Dinghu Mountain, Guangdong, China	MBC	0-5	forest	23.09 N	20.9	1956	NH_4NO_3	100	8	0.0215	0.0322	
Fang et al. 2012	Dinghu Mountain, Guangdong, China	MBC	0-5	forest	23.09 N	20.9	1956	NH_4NO_3	50	8	0.2534	0.0742	
Fang et al. 2012	Dinghu Mountain, Guangdong, China	MBC	5-10	forest	23.09 N	20.9	1956	NH_4NO_3	150	8	-0.1322	0.0208	
Fang et al. 2012	Dinghu Mountain, Guangdong, China	MBC	5-10	forest	23.09 N	20.9	1956	NH_4NO_3	100	8	0.0226	0.0214	
Fang et al. 2012	Dinghu Mountain, Guangdong, China	MBC	5-10	forest	23.09 N	20.9	1956	NH_4NO_3	50	8	-0.1019	0.0503	
Fang et al. 2012	Dinghu Mountain, Guangdong, China	MBC	10-20	forest	23.09 N	20.9	1956	NH_4NO_3	150	8	-0.2099	0.0398	
Fang et al. 2012	Dinghu Mountain, Guangdong, China	MBC	10-20	forest	23.09 N	20.9	1956	NH_4NO_3	100	8	-0.4018	0.1063	
Fang et al. 2012	Dinghu Mountain, Guangdong, China	MBC	10-20	forest	23.09 N	20.9	1956	NH_4NO_3	50	8	-0.1014	0.0823	
Fang et al. 2012	Dinghu Mountain, Guangdong, China	MBC	20-30	forest	23.09 N	20.9	1956	NH_4NO_3	150	8	0.2042	0.062	
Fang et al. 2012	Dinghu Mountain, Guangdong, China	MBC	20-30	forest	23.09 N	20.9	1956	NH_4NO_3	100	8	-0.2937	0.029	
Fang et al. 2012	Dinghu Mountain, Guangdong, China	MBC	20-30	forest	23.09 N	20.9	1956	NH_4NO_3	50	8	0.103	0.0739	
Fang et al. 2012	Dinghu Mountain, Guangdong, China	MBC	30-50	forest	23.09 N	20.9	1956	NH_4NO_3	150	8	-0.0469	0.1576	
Fang et al. 2012	Dinghu Mountain, Guangdong, China	MBC	30-50	forest	23.09 N	20.9	1956	NH_4NO_3	100	8	-0.7863	0.2044	
Fang et al. 2012	Dinghu Mountain, Guangdong, China	MBC	30-50	forest	23.09 N	20.9	1956	NH_4NO_3	50	8	-0.5138	0.0494	
Fang et al. 2012	Dinghu Mountain, Guangdong, China	MBC	0-5	forest	23.09 N	20.9	1956	NH_4NO_3	100	8	-0.0273	0.0501	
Fang et al. 2012	Dinghu Mountain, Guangdong, China	MBC	0-5	forest	23.09 N	20.9	1956	NH_4NO_3	50	8	-0.2044	0.1207	
Fang et al. 2012	Dinghu Mountain, Guangdong, China	MBC	5-10	forest	23.09 N	20.9	1956	NH_4NO_3	100	8	-0.5138	0.838	

Fang et al. 2012	Dinghu Mountain, Guangdong, China	MBC	5-10	forest	23.09 N	20.9	1956	NH_4NO_3	50	8	-0.4538	0.6646	
Fang et al. 2012	Dinghu Mountain, Guangdong, China	MBC	10-20	forest	23.09 N	20.9	1956	NH_4NO_3	100	8	-2.084	28.8313	
Fang et al. 2012	Dinghu Mountain, Guangdong, China	MBC	10-20	forest	23.09 N	20.9	1956	NH_4NO_3	50	8	-0.1131	1.6174	
Fang et al. 2012	Dinghu Mountain, Guangdong, China	MBC	0-5	forest	23.09 N	20.9	1956	NH_4NO_3	100	8	0.0289	0.0439	
Fang et al. 2012	Dinghu Mountain, Guangdong, China	MBC	0-5	forest	23.09 N	20.9	1956	NH_4NO_3	50	8	0.3592	0.0521	
Fang et al. 2012	Dinghu Mountain, Guangdong, China	MBC	5-10	forest	23.09 N	20.9	1956	NH_4NO_3	100	8	0.5378	0.6911	
Fang et al. 2012	Dinghu Mountain, Guangdong, China	MBC	5-10	forest	23.09 N	20.9	1956	NH_4NO_3	50	8	0.9748	0.2015	
Fang et al. 2012	Dinghu Mountain, Guangdong, China	MBC	20-30	forest	23.09 N	20.9	1956	NH_4NO_3	100	8	-3.25	224.8703	
Fang et al. 2012	Dinghu Mountain, Guangdong, China	MBC	20-30	forest	23.09 N	20.9	1956	NH_4NO_3	50	8	-0.0414	0.7843	
Tu et al. 2012	Lingbao Mountain, Shanxi, China	soil TOC	0-10	forest	36.38 N	8.6	662	urea	50	1	0.0823	0.0173	
Tu et al. 2012	Lingbao Mountain, Shanxi, China	soil TOC	0-10	forest	36.38 N	8.6	662	urea	100	1	0.0313	0.0145	
Quan et al. 2015	Dongling Mountain, Beijing, China	soil respiration		forest	39.57 N	11	634	NH_4NO_3	100	1	-0.0333	0.0012	
Quan et al. 2015	Dongling Mountain, Beijing, China	soil respiration		forest	39.57 N	11	634	NH_4NO_3	100	1	0.0382	0.0011	
Quan et al. 2015	Dongling Mountain, Beijing, China	soil respiration		forest	39.57 N	11	634	NH_4NO_3	100	1	0.0483	0.0005	
Yang et al. 2014	Menyuan County, Qinghai, China	plant aboveground part C		grassland	37.29 N	-1.7	561	urea	100	4	0.335	0.0106	total
Yang et al. 2014	Menyuan County, Qinghai, China	plant belowground part C		grassland	37.29 N	-1.7	561	urea	100	4	-0.089	0.0202	total
Lin et al. 2012	Daqinggou, Inner Mongolia, China	MBC	0-10	forest	42.54 N	6	450	urea	80	1	0.3011	0.0427	
Yu et al. 2013	Hunan, China	MBC	0-10	forest	28.06 N	17.3	1422	NH_4NO_3	50	1	-0.0847	0.0249	
Yu et al. 2013	Hunan, China	MBC	0-10	forest	28.06 N	17.3	1422	NH_4NO_3	150	1	-0.3182	0.0139	
Long et al. 2014	Guangdong, China	soil TOC	0-5	forest	23.18 N	21.5	1700	NH_4NO_3	100	5	-0.0121	0.0144	
Long et al. 2014	Guangdong, China	soil TOC	5-10	forest	23.18 N	21.5	1700	NH_4NO_3	100	5	0.1829	0.0203	
Long et al. 2014	Guangdong, China	soil TOC	10-20	forest	23.18 N	21.5	1700	NH_4NO_3	100	5	-0.0271	0.032	
Long et al. 2014	Guangdong, China	soil TOC	20-40	forest	23.18 N	21.5	1700	NH_4NO_3	100	5	-0.1144	0.0194	
Long et al. 2014	Guangdong, China	soil TOC	40-60	forest	23.18 N	21.5	1700	NH_4NO_3	100	5	0.1051	0.0122	
Long et al. 2014	Guangdong, China	MBC	0-5	forest	23.18 N	21.5	1700	NH_4NO_3	100	5	0.5447	0.0463	
Long et al. 2014	Guangdong, China	MBC	5-10	forest	23.18 N	21.5	1700	NH_4NO_3	100	5	0.7348	0.0981	

Long et al. 2014	Guangdong, China	MBC	10-20	forest	23.18 N	21.5	1700	NH_4NO_3	100	5	0.0889	0.0356		
Long et al. 2014	Guangdong, China	MBC	20-40	forest	23.18 N	21.5	1700	NH_4NO_3	100	5	0.2744	0.5294		
Bejarano et al. 2014	Yucatan Peninsula, Mexico	litter decomposition rate		forest	24.14 N	25.8	531	urea	2.4	1	-0.1141	0.0024	*Gymnopodium*	herbaceous
Bejarano et al. 2014	Yucatan Peninsula, Mexico	litter decomposition rate		forest	24.14 N	25.8	531	urea	24	1	-0.0145	0.0054	*Gymnopodium*	herbaceous
Bejarano et al. 2014	Yucatan Peninsula, Mexico	litter decomposition rate		forest	20.51 N	26.6	993	urea	2.4	1	0.0536	0.0015	*Gymnopodium*	herbaceous
Bejarano et al. 2014	Yucatan Peninsula, Mexico	litter decomposition rate		forest	20.51 N	26.6	993	urea	24	1	0.0271	0.0011	*Gymnopodium*	herbaceous
Bejarano et al. 2014	Yucatan Peninsula, Mexico	litter decomposition rate		forest	19.38 N	26.2	1035	urea	2.4	1	-0.1541	0.0033	*Gymnopodium*	herbaceous
Bejarano et al. 2014	Yucatan Peninsula, Mexico	litter decomposition rate		forest	19.38 N	26.2	1035	urea	24	1	-0.3054	0.014	*Gymnopodium*	herbaceous
Bejarano et al. 2014	Yucatan Peninsula, Mexico	litter decomposition rate		forest	24.14 N	25.8	531	urea	2.4	1	0.0203	0.0001	*Piscidia*	woody
Bejarano et al. 2014	Yucatan Peninsula, Mexico	litter decomposition rate		forest	24.14 N	25.8	531	urea	24	1	-0.0529	0.0001	*Piscidia*	woody
Bejarano et al. 2014	Yucatan Peninsula, Mexico	litter decomposition rate		forest	20.51 N	26.6	993	urea	2.4	1	0.009	0.0001	*Piscidia*	woody
Bejarano et al. 2014	Yucatan Peninsula, Mexico	litter decomposition rate		forest	20.51 N	26.6	993	urea	24	1	0.0181	0.0002	*Piscidia*	woody
Bejarano et al. 2014	Yucatan Peninsula, Mexico	litter decomposition rate		forest	19.38 N	26.2	1035	urea	2.4	1	0.0075	0.0019	*Piscidia*	woody
Bejarano et al. 2014	Yucatan Peninsula, Mexico	litter decomposition rate		forest	19.38 N	26.2	1035	urea	24	1	-0.6854	0.0048	*Piscidia*	woody
Hobbie 2000	Hawaii Volcanoes National Park, USA	litter decomposition rate		forest	19.6 N	20	340	NH_4NO_3	100	30 months	0.1399	0.0017	*Metrosideros*	woody
Hobbie 2000	Hawaii Volcanoes National Park, USA	litter decomposition rate		forest	19.6 N	16	4300	NH_4NO_3	100	30 months	0.3422	0.0075	*Metrosideros*	woody
Hobbie 2000	Hawaii Volcanoes National Park, USA	litter decomposition rate		forest	19.6 N	10	1200	NH_4NO_3	100	30 months	0.1485	0.001	*Metrosideros*	woody
Hobbie 2000	Hawaii Volcanoes National Park, USA	litter decomposition rate		forest	19.6 N	16	2500	NH_4NO_3	100	30 months	0.3644	0.0085	*Metrosideros*	woody
Hobbie 2000	Hawaii Volcanoes National Park, USA	litter decomposition rate		forest	19.6 N	20	340	NH_4NO_3	100	30 months	0.0982	0.0017	*Metrosideros*	woody
Deng et al. 2013	Guangdong, China	plant aboveground part C		forest	23.2 N	21.5	1714	NH_4NO_3	100	5	0.5014	0.0054		woody
Deng et al. 2013	Guangdong, China	plant belowground part C		forest	23.2 N	21.5	1714	NH_4NO_3	100	5	0.3721	0.0087		woody
Deng et al. 2013	Guangdong, China	soil respiration		forest	23.2 N	21.5	1714	NH_4NO_3	100	5	0.0717	0.0062		
Carreiro et al. 2000	Armonk, New York, USA	litter decomposition rate		forest	41.07 N	11.2	1140	NH_4NO_3	20	12 months	0.2317	0.0088	*Cornus florida*	woody
Carreiro et al. 2000	Armonk, New York, USA	litter decomposition rate		forest	41.07 N	11.2	1140	NH_4NO_3	80	12 months	0.2347	0.0087	*Cornus florida*	woody
Carreiro et al. 2000	Armonk, New York, USA	litter decomposition rate		forest	41.07 N	11.2	1140	NH_4NO_3	20	24 months	0.0361	0.0059	*Acer rubrum*	woody
Carreiro et al. 2000	Armonk, New York, USA	litter decomposition rate		forest	41.07 N	11.2	1140	NH_4NO_3	80	24 months	-0.1252	0.0066	*Acer rubrum*	woody

Carreiro et al. 2000	Armonk, New York, USA	litter decomposition rate		forest	41.07 N	11.2	1140	NH_4NO_3	20	36 months	-0.1636	0.0095	*Quercus rubra*	woody
Carreiro et al. 2000	Armonk, New York, USA	litter decomposition rate		forest	41.07 N	11.2	1140	NH_4NO_3	80	36 months	-0.2881	0.0108	*Quercus rubra*	woody
Jia et al. 2012	Shenmu County, Loess Plateau, China	soil respiration		grassland	38.49 N	8.4	405	urea	200	1	0.3093	0.0009	*Leymus chinensis*	herbaceous
Jia et al. 2012	Shenmu County, Loess Plateau, China	plant aboveground part C		grassland	38.49 N	8.4	405	urea	200	1	0.935	0.0037		total
Gong et al. 2015	Songnen Grassland, Northeast China	litter decomposition rate		grassland	44.4 N	6.4	470	NH_4NO_3	100	36 months	0.4068	0.0286	*Phragmites communis*	herbaceous
Gong et al. 2015	Songnen Grassland, Northeast China	litter decomposition rate		grassland	44.4 N	6.4	470	NH_4NO_3	100	36 months	0.3724	0.0188		
Liu et al. 2010	Duolun County, Inner Mongolia, China	DOC	0-15	grassland	42.27 N	2.1	385	urea	150	3	-0.1801	0.0286		
Liu et al. 2010	Duolun County, Inner Mongolia, China	DOC	0-15	grassland	42.27 N	2.1	385	urea	150	3	-0.1386	0.0185		
Liu et al. 2010	Duolun County, Inner Mongolia, China	MBC	0-15	grassland	42.27 N	2.1	385	urea	150	3	-0.452	0.0247		
Liu et al. 2010	Duolun County, Inner Mongolia, China	MBC	0-15	grassland	42.27 N	2.1	385	urea	150	3	-0.4055	0.0409		
Liu et al. 2010	Duolun County, Inner Mongolia, China	microbial respiration	0-15	grassland	42.27 N	2.1	385	urea	150	3	-0.2088	0.0393		
Liu et al. 2010	Duolun County, Inner Mongolia, China	microbial respiration	0-15	grassland	42.27 N	2.1	385	urea	150	3	-0.2056	0.0193		
Zhang et al. 2008	Duolun County, Inner Mongolia, China	MBC	0-15	grassland	42.02 N	2.1	385	urea	10	3	0.0421	0.0544		
Zhang et al. 2008	Duolun County, Inner Mongolia, China	MBC	0-15	grassland	42.02 N	2.1	385	urea	20	3	-0.4652	0.0597		
Zhang et al. 2008	Duolun County, Inner Mongolia, China	MBC	0-15	grassland	42.02 N	2.1	385	urea	40	3	0.0484	0.0785		
Zhang et al. 2008	Duolun County, Inner Mongolia, China	MBC	0-15	grassland	42.02 N	2.1	385	urea	80	3	-0.0082	0.1446		
Zhang et al. 2008	Duolun County, Inner Mongolia, China	MBC	0-15	grassland	42.02 N	2.1	385	urea	160	3	0.3403	0.0548		
Zhang et al. 2008	Duolun County, Inner Mongolia, China	MBC	0-15	grassland	42.02 N	2.1	385	urea	320	3	-0.0955	0.0527		
Zhang et al. 2008	Duolun County, Inner Mongolia, China	MBC	0-15	grassland	42.02 N	2.1	385	urea	640	3	-0.5015	0.1288		
Zhang et al. 2008	Duolun County, Inner Mongolia, China	soil TOC	0-15	grassland	42.02 N	2.1	385	urea	10	3	-0.0177	0.0062		
Zhang et al. 2008	Duolun County, Inner Mongolia, China	soil TOC	0-15	grassland	42.02 N	2.1	385	urea	20	3	-0.2691	0.0035		
Zhang et al. 2008	Duolun County, Inner Mongolia, China	soil TOC	0-15	grassland	42.02 N	2.1	385	urea	40	3	0.0213	0.0062		
Zhang et al. 2008	Duolun County, Inner Mongolia, China	soil TOC	0-15	grassland	42.02 N	2.1	385	urea	80	3	-0.0354	0.0027		

Zhang et al. 2008	Duolun County, Inner Mongolia, China	soil TOC	0-15	grassland	42.02 N	2.1	385	urea	160	3	0.1624	0.0711	
Zhang et al. 2008	Duolun County, Inner Mongolia, China	soil TOC	0-15	grassland	42.02 N	2.1	385	urea	320	3	0.0107	0.003	
Zhang et al. 2008	Duolun County, Inner Mongolia, China	soil TOC	0-15	grassland	42.02 N	2.1	385	urea	640	3	-0.1074	0.0041	
Ares and Fownes 2001	Honaunau Forest, Hawaii, USA	litterfall		forest	19.00 N	14	4968	urea	500	2	0.2557	0.0902	
Ares and Fownes 2001	Honaunau Forest, Hawaii, USA	litterfall		forest	19.00 N	14	4968	urea	500	2	0.0237	0.1692	
Ares and Fownes 2001	Honaunau Forest, Hawaii, USA	litterfall		forest	19.00 N	14	4968	urea	500	2	0.0701	0.0072	
Ares and Fownes 2001	Honaunau Forest, Hawaii, USA	ANPP		forest	19.00 N	14	4968	urea	500	2	0.7117	0.0291	woody
Ares and Fownes 2001	Honaunau Forest, Hawaii, USA	ANPP		forest	19.00 N	14	4968	urea	500	2	-0.094	0.2206	woody
Ares and Fownes 2001	Honaunau Forest, Hawaii, USA	ANPP		forest	19.00 N	14	4968	urea	500	2	0.1795	0.118	woody
Basiliko et al. 2009	Kenneth Creek, Canada	soil TOC	0-5	forest	53.49 N	3	650	urea	200	1	0.8408	0.0776	
Basiliko et al. 2009	Kenneth Creek, Canada	soil TOC	5-10	forest	53.49 N	3	650	urea	200	1	0.494	0.0256	
Basiliko et al. 2009	Kenneth Creek, Canada	MBC	0-5	forest	53.49 N	3	650	urea	200	1	0.6931	0.3212	
Basiliko et al. 2009	Kenneth Creek, Canada	MBC	5-10	forest	53.49 N	3	650	urea	200	1	0.4055	0.2222	
Zhou et al. 2012	Gurbantunggut Desert, Urumqi, Xingjiang, China	soil TOC	0-5	desert	44.87 N	7.4	79.5	NH_4NO_3	5	2	0	0.0139	
Zhou et al. 2012	Gurbantunggut Desert, Urumqi, Xingjiang, China	soil TOC	0-5	desert	44.87 N	7.4	79.5	NH_4NO_3	10	2	0.1542	0.0274	
Zhou et al. 2012	Gurbantunggut Desert, Urumqi, Xingjiang, China	soil TOC	0-5	desert	44.87 N	7.4	79.5	NH_4NO_3	30	2	0.1542	0.0529	
Zhou et al. 2012	Gurbantunggut Desert, Urumqi, Xingjiang, China	soil TOC	0-5	desert	44.87 N	7.4	79.5	NH_4NO_3	60	2	0.2231	0.0247	
Zhou et al. 2012	Gurbantunggut Desert, Urumqi, Xingjiang, China	soil TOC	0-5	desert	44.87 N	7.4	79.5	NH_4NO_3	240	2	0.4595	0.0097	
Zhou et al. 2012	Gurbantunggut Desert, Urumqi, Xingjiang, China	soil TOC	5-10	desert	44.87 N	7.4	79.5	NH_4NO_3	5	2	-0.1335	0.036	
Zhou et al. 2012	Gurbantunggut Desert, Urumqi,	soil TOC	5-10	desert	44.87 N	7.4	79.5	NH_4NO_3	10	2	0	0.0313	

	Xingjiang, China												
Zhou et al. 2012	Gurbantunggut Desert, Urumqi, Xingjiang, China	soil TOC	5-10	desert	44.87 N	7.4	79.5	NH_4NO_3	30	2	-0.2877	0.0157	
Zhou et al. 2012	Gurbantunggut Desert, Urumqi, Xingjiang, China	soil TOC	5-10	desert	44.87 N	7.4	79.5	NH_4NO_3	60	2	-0.2877	0.0157	
Zhou et al. 2012	Gurbantunggut Desert, Urumqi, Xingjiang, China	soil TOC	5-10	desert	44.87 N	7.4	79.5	NH_4NO_3	240	2	-0.2877	0.0434	
Niu et al. 2009	Duolun County, Inner Mongolia, China	GEP		grassland	42.02 N	2.1	385	urea	100	1	0.3159	0.0067	
Niu et al. 2009	Duolun County, Inner Mongolia, China	ER		grassland	42.02 N	2.1	385	urea	100	1	0.2007	0.0128	
Koehler et al. 2009a	Gigante Peninsula, Panama	soil respiration		forest	9.06 N	27.4	2650	urea	125	3	-0.0821	0.0044	
Koehler et al. 2009a	Gigante Peninsula, Panama	soil respiration		forest	9.06 N	27.4	2650	urea	125	11	0.0182	0.0142	
Tanner et al. 1992	Parque Nacional de Sierra Nevada	litter C		forest	8 N	13	2500	urea	225	2	0.0492	0.0069	
Verhoeven & Schmitz 1991	Eastern Vechtplassen area, The Netherlands	plant aboveground part C		wetland	52.22 N	9	800	NH_4NO_3	200	1	0.3087	0.0021	vascular plants
Verhoeven & Schmitz 1991	Eastern Vechtplassen area, The Netherlands	plant aboveground part C		wetland	52.22 N	9	800	NH_4NO_3	200	1	-0.1286	0.0393	vascular plants
Verhoeven & Schmitz 1991	Eastern Vechtplassen area, The Netherlands	plant aboveground part C		wetland	52.22 N	9	800	NH_4NO_3	200	1	0.175	0.0205	vascular plants
Barger et al.2002	Caracas, Venezuela	plant aboveground part C		grassland	10.5 N	17.9	1063	urea	200	1	0.5655	0.0249	total
Davidson et al. 2004	Fazenda Vitoria, Para State, Brazil	plant aboveground part C		forest	2.59 S	27	1800	urea	100	3	0.392	0.1717	total
Augustine et al. 2003	Central Kenya	ANPP		grassland	0.17 N	19	508	urea	400	3	0.2923	0.0244	herbaceous
McMaster et al. 1982	Echo Valley, California, USA	plant aboveground part C		desert	32.93 N	13	476	urea	80	2	0.6893	0.0299	herbaceous
Boeye et al. 1997	Buitengoor, Belgium	plant aboveground part C		wetland	51.12 N	10	345	NH_4NO_3	200	2	0.1513	0.0154	total
van Duren et al. 1997	Hasselt, Belgium	plant aboveground part C		wetland	56.61 N	10	800	urea	200	2	0.3029	0.0113	total
van Duren et al. 1997	Hasselt, Belgium	plant aboveground part C		wetland	56.61 N	10	800	urea	200	2	-0.2119	0.0082	total
Mirmanto et al. 1999	Central Kalimantan, Indonesia	litterfall		forest	0.6 S	27	3600	urea	225	3	0.2482	0.0138	

Herbert & Fownes 1995	Island of Kauai, Hawaii, USA	litterfall		forest	22.08 N	16	2500	urea, NH_4NO_3	100	2	0.2065	0.0039	
Herbert & Fownes 1995	Island of Kauai, Hawaii, USA	ANPP		forest	22.08 N	16	2500	urea, NH_4NO_3	100	2	0.0855	0.0104	woody
Harrington et al. 2001	Island of Kauai, Hawaii, USA	ANPP		forest	19.42 N	16	2500	urea, NH_4NO_3	100	6	0.5489	0.0616	woody
Harrington et al. 2001	Island of Kauai, Hawaii, USA	ANPP		forest	22.13 N	16	2500	urea, NH_4NO_3	100	6	0.1872	0.0225	woody
Haag 1974	Tuktoyaktuk, Northwest Territories, Canada	ANPP		tundra	69 N	-10	266	NH_4NO_3	100	1	0.3707	0.0049	total
Haag 1974	Tuktoyaktuk, Northwest Territories, Canada	ANPP		tundra	69 N	-10	266	NH_4NO_3	200	1	0.5234	0.0049	total
Haag 1974	Tuktoyaktuk, Northwest Territories, Canada	ANPP		tundra	69 N	-10	266	NH_4NO_3	100	1	0.6509	0.0091	total
Haag 1974	Tuktoyaktuk, Northwest Territories, Canada	ANPP		tundra	69 N	-10	266	NH_4NO_3	200	1	0.4871	0.0048	total
Baer et al. 2003	Konza Prairie Biological, Kansas, USA	soil total C	0-10	grassland	39.05 N	13	835	NH_4NO_3	50	3	1.6139	0.0026	
Baer et al. 2003	Konza Prairie Biological, Kansas, USA	MBC	0-10	grassland	39.05 N	13	835	NH_4NO_3	50	3	0.1796	0.01	
Baer et al. 2003	Konza Prairie Biological, Kansas, USA	ANPP		grassland	39.05 N	13	835	NH_4NO_3	50	3	0.2026	0.0121	total
Camill et al. 2004	Northfield, Minnesota, USA	ANPP		grassland	44.47 N	4	767	NH_4NO_3	100	1	0.4264	0.0051	total
Camill et al. 2004	Northfield, Minnesota, USA	ANPP		grassland	44.47 N	4	767	NH_4NO_3	100	1	0.3287	0.0037	total
Camill et al. 2004	Northfield, Minnesota, USA	BNPP		grassland	44.47 N	4	767	NH_4NO_3	100	1	0.2199	0.0113	total
Camill et al. 2004	Northfield, Minnesota, USA	BNPP		grassland	44.47 N	4	767	NH_4NO_3	100	1	0.0956	0.004	total
Gough & Hobbie 2003	Brooks Range, Alaska, USA	ANPP		tundra	68.38 N	-9	310	NH_4NO_3	100	4	0.1293	0.019	total
Gough & Hobbie 2003	Brooks Range, Alaska, USA	plant aboveground part C		tundra	68.38 N	-9	310	NH_4NO_3	100	4	-0.0765	0.0171	total
Gough & Hobbie 2003	Brooks Range, Alaska, USA	plant belowground part C		tundra	68.38 N	-9	310	NH_4NO_3	100	4	-0.204	0.046	total

van Wijnen & Bakker 1999	Island of Schiermonnikoog, The Netherlands	plant aboveground part C	wetland	53.3 N	9	820	NH_4NO_3	50	3	0.2382	0.0314	total
van Wijnen & Bakker 1999	Island of Schiermonnikoog, The Netherlands	plant aboveground part C	wetland	53.3 N	9	820	NH_4NO_3	250	3	0.3406	0.0198	total
van Wijnen & Bakker 1999	Island of Schiermonnikoog, The Netherlands	plant aboveground part C	wetland	53.3 N	9	820	NH_4NO_3	50	3	-0.0447	0.016	total
van Wijnen & Bakker 1999	Island of Schiermonnikoog, The Netherlands	plant aboveground part C	wetland	53.3 N	9	820	NH_4NO_3	250	3	0.316	0.0117	total
Ludwing et al. 2001	Tarangire National Park, Northern Tanzania	plant aboveground part C	grassland	4.48 N	24	650	NO_3	200	2	0.0532	0.0775	herbaceous
Ludwing et al. 2001	Tarangire National Park, Northern Tanzania	plant aboveground part C	grassland	4.48 N	24	650	NO_3	200	2	0.1869	0.0114	herbaceous
Berendse et al. 2001	Salmisuo, Ilomantsi, eastern Finland	ANPP	wetland	62.47 N	3	650	NH_4NO_3	30	3	0.156	0.0321	moss
Berendse et al. 2001	Kopparasmyren, southern Sweden	ANPP	wetland	57.8 N	7	800	NH_4NO_3	30	3	-0.1211	0.0255	moss
Berendse et al. 2001	La Chaux-des-Breuleux, Swiss Jura, Switzerland	ANPP	wetland	47.13 N	5	1390	NH_4NO_3	30	3	-1.0225	0.0504	moss
Berendse et al. 2001	Peat from Dwingeloo, The Netherlands	ANPP	wetland	52.49 N	10.5	750	NH_4NO_3	50	3	-0.451	0.0224	moss
Berendse et al. 2001	Salmisuo, Ilomantsi, eastern Finland	plant aboveground part C	wetland	62.47 N	3	650	NH_4NO_3	30	3	0.1423	0.0196	total
Berendse et al. 2001	Kopparasmyren, southern Sweden	plant aboveground part C	wetland	57.8 N	7	800	NH_4NO_3	30	3	-0.1077	0.0676	total
Berendse et al. 2001	La Chaux-des-Breuleux, Swiss Jura, Switzerland	plant aboveground part C	wetland	47.13 N	5	1390	NH_4NO_3	30	3	-0.0073	0.1559	total
Berendse et al. 2001	Peat from Dwingeloo, The Netherlands	plant aboveground part C	wetland	52.49 N	10.5	750	NH_4NO_3	50	3	0.349	0.041	total
Berendse et al. 2001	Salmisuo, Ilomantsi, eastern Finland	plant belowground part C	wetland	62.47 N	3	650	NH_4NO_3	30	3	-0.0284	0.0562	total
Berendse et al. 2001	Kopparasmyren, southern Sweden	plant belowground part C	wetland	57.8 N	7	800	NH_4NO_3	30	3	-0.3154	0.1401	total
Berendse et al. 2001	La Chaux-des-Breuleux, Swiss Jura, Switzerland	plant belowground part C	wetland	47.13 N	5	1390	NH_4NO_3	30	3	-0.2094	0.1115	total

Berendse et al. 2001	Peat from Dwingeloo, The Netherlands	plant belowground part C	wetland	52.49 N	10.5	750	NH_4NO_3	50	3	0.1214	0.0421	total
Aydin & Uzun 2005	The Blacksea cost of Turkey	ANPP	grassland	41.21 N	14	665	NH_4	60	3	0.4273	0.0079	total
Aydin & Uzun 2005	The Blacksea cost of Turkey	ANPP	grassland	41.21 N	14	665	NH_4	120	3	0.5118	0.0122	total
Aydin & Uzun 2005	The Blacksea cost of Turkey	ANPP	grassland	41.21 N	14	665	NH_4	180	3	0.7971	0.0042	total
Olde Venterink et al. 2001	Zwarte Beek, Belgium	ANPP	wetland	5.20 N	10	825	NH_4NO_3	200	1	0.2174	0.0163	vascular plants
Olde Venterink et al. 2001	Zwarte Beek, Belgium	ANPP	wetland	5.20 N	10	825	NH_4NO_3	200	1	0.5405	0.0351	vascular plants
Olde Venterink et al. 2001	Zwarte Beek, Belgium	ANPP	wetland	5.20 N	10	825	NH_4NO_3	200	1	0.5902	0.0244	vascular plants
Olde Venterink et al. 2001	Zwarte Beek, Belgium	ANPP	wetland	5.20 N	10	825	NH_4NO_3	200	1	0.1273	0.003	vascular plants
Olde Venterink et al. 2001	Zwarte Beek, Belgium	ANPP	wetland	5.20 N	10	825	NH_4NO_3	200	1	0.0883	0.017	vascular plants
Olde Venterink et al. 2001	Zwarte Beek, Belgium	ANPP	wetland	5.20 N	10	825	NH_4NO_3	200	1	0.1032	0.0129	vascular plants
Thormann & Bayley 1997	southern boreal Alberta, Canada	ANPP	wetland	54.68 N	2	332	NH_4NO_3	150	1	-0.3421	0.0032	vascular plants
Thormann & Bayley 1997	southern boreal Alberta, Canada	ANPP	wetland	54.17 N	2	332	NH_4NO_3	150	1	0.1775	0.0135	vascular plants
Thormann & Bayley 1997	southern boreal Alberta, Canada	ANPP	wetland	54.47 N	2	332	NH_4NO_3	150	1	-0.0513	0.0055	vascular plants
Thormann & Bayley 1997	southern boreal Alberta, Canada	ANPP	wetland	54.47 N	2	332	NH_4NO_3	150	1	0	0.1475	vascular plants
Thormann & Bayley 1997	southern boreal Alberta, Canada	ANPP	wetland	54.47 N	2	332	NH_4NO_3	150	1	0.3893	0.0325	vascular plants

Turner et al. 1997	eastern Kansas, USA	ANPP		grassland	39.05 N	12	835	NH_4NO_3	100	2	0.4768	0.0103	total
Turner et al. 1997	eastern Kansas, USA	ANPP		grassland	39.05 N	12	835	NH_4NO_3	100	2	0.4371	0.0179	total
Turner et al. 1997	eastern Kansas, USA	ANPP		grassland	39.05 N	12	835	NH_4NO_3	100	2	0.3364	0.073	total
Turner et al. 1997	eastern Kansas, USA	ANPP		grassland	39.05 N	12	835	NH_4NO_3	100	2	0.1274	0.0286	total
Lugato et al. 2006	Padova experimental farm, Italy	soil TOC	0-30	cropland	45.21 N	12.4	850	urea, NH_4NO_3	60	20	0.0064	0.002	
Lugato et al. 2006	Padova experimental farm, Italy	soil TOC	0-30	cropland	45.21 N	12.4	850	urea, NH_4NO_3	120	20	0.0377	0.0024	
Lugato et al. 2006	Padova experimental farm, Italy	soil TOC	0-30	cropland	45.21 N	12.4	850	urea, NH_4NO_3	180	20	0.0377	0.0024	
Lugato et al. 2006	Padova experimental farm, Italy	soil TOC	0-30	cropland	45.21 N	12.4	850	urea, NH_4NO_3	240	20	0.0561	0.0034	
Bradley et al. 2006	Cedar Creek, Minnesota, USA	plant aboveground part C		grassland	45.24 N	6	810	NH_4NO_3	54.4	14	0.0253	0.0031	total
Bradley et al. 2006	Cedar Creek, Minnesota, USA	plant aboveground part C		grassland	45.24 N	6	810	NH_4NO_3	272	14	0.0858	0.0015	total
Bradley et al. 2006	Cedar Creek, Minnesota, USA	litter C		grassland	45.24 N	6	810	NH_4NO_3	54.4	14	0.3787	0.0275	
Bradley et al. 2006	Cedar Creek, Minnesota, USA	litter C		grassland	45.24 N	6	810	NH_4NO_3	272	14	0.9628	0.0158	
Bradley et al. 2006	Cedar Creek, Minnesota, USA	soil total C	0-15	grassland	45.24 N	6	810	NH_4NO_3	54.4	14	0.1823	0.0073	
Bradley et al. 2006	Cedar Creek, Minnesota, USA	soil total C	0-15	grassland	45.24 N	6	810	NH_4NO_3	272	14	0.3553	0.0036	
Bradley et al. 2006	Cedar Creek, Minnesota, USA	soil respiration		grassland	45.24 N	6	810	NH_4NO_3	54.4	14	0.1687	0.0097	
Bradley et al. 2006	Cedar Creek, Minnesota, USA	soil respiration		grassland	45.24 N	6	810	NH_4NO_3	272	14	0.3756	0.0189	
Xu & Wan 2008	Duolun County, Inner Mongolia, China	soil respiration		grassland	42.27 N	2.1	385	urea	150	2	0.1058	0.0037	
Xu & Wan 2008	Duolun County, Inner Mongolia, China	soil respiration		grassland	42.27 N	2.1	385	urea	150	2	0.1828	0.0059	
Xu & Wan 2008	Duolun County, Inner Mongolia, China	soil respiration		grassland	42.27 N	2.1	385	urea	150	2	-0.1089	0.0054	
Xu & Wan 2008	Duolun County, Inner Mongolia, China	soil respiration		grassland	42.27 N	2.1	385	urea	150	2	0.2376	0.004	
Bechtold & Inouye 2007	Southeastern Idaho, USA	soil total C	0-5	grassland	42.85 N	7.9	321	NH_4NO_3	60	6	-0.0586	0.0162	
Bechtold & Inouye 2007	Southeastern Idaho, USA	soil total C	5-10	grassland	42.85 N	7.9	321	NH_4NO_3	60	6	-0.0351	0.0182	

Bechtold & Inouye 2007	Southeastern Idaho, USA	soil total C	0-5	grassland	42.85 N	7.9	321	NH_4NO_3	60	6	-0.0211	0.0086		
Bechtold & Inouye 2007	Southeastern Idaho, USA	soil total C	5-10	grassland	42.85 N	7.9	321	NH_4NO_3	60	6	0.1834	0.0178		
Hati et al. 2007	Jabalpur, India	soil TOC	0-15	cropland	23.14 N	26	1253	urea	80	28	0.069	0.0058		
Micks et al. 2004	Harvard Forest, Massachusetts, USA	soil respiration		forest	42.32 N	6.5	1100	NH_4NO_3	150	5	-0.1558	0.0087		
Micks et al. 2004	Harvard Forest, Massachusetts, USA	soil respiration		forest	42.32 N	6.5	1100	NH_4NO_3	150	5	-0.1361	0.0082		
Liu et al. 2006	Inner Mongolia, China	litter decomposition rate		grassland	42.02 N	1.6	1748	urea	80	3	0.0328	0.0099	*Allium*	herbaceous
Liu et al. 2006	Inner Mongolia, China	litter decomposition rate		grassland	42.02 N	1.6	1748	urea	160	3	0.08	0.0093	*Allium*	herbaceous
Liu et al. 2006	Inner Mongolia, China	litter decomposition rate		grassland	42.02 N	1.6	1748	urea	320	3	0.1029	0.007	*Allium*	herbaceous
Liu et al. 2006	Inner Mongolia, China	litter decomposition rate		grassland	42.02 N	1.6	1748	urea	80	3	0.0902	0.032	*Stipa*	herbaceous
Liu et al. 2006	Inner Mongolia, China	litter decomposition rate		grassland	42.02 N	1.6	1748	urea	160	3	0.1728	0.0426	*Stipa*	herbaceous
Liu et al. 2006	Inner Mongolia, China	litter decomposition rate		grassland	42.02 N	1.6	1748	urea	320	3	0.2344	0.0255	*Stipa*	herbaceous
Aerts et al. 2003	Amerongse Bovenpolder, the Netherlands	litterfall		grassland	51.54 N	11.2	786	NH_4NO_3	100	12	0.3564	0.1394		
Aerts et al. 2003	Utrecht, the Netherlands	litterfall		grassland	52.04 N	10.1	833	NH_4NO_3	100	12	0.2605	0.0224		
Balik et al. 2003	Cerveny Ujezd, Czech	soil total C	0-25	cropland	50.50 N	7.6	549	NH_4	120	4	-0.0658	0.0855		
Allard et al. 2006	FACE experimental pasture, the New Zealand	MBC		forest	40.14 S	12.9	870	NH_4NO_3	100	1	0.2533	0.0001		
Allard et al. 2006	FACE experimental pasture, the New Zealand	soil respiration		forest	40.14 S	12.9	870	NH_4NO_3	100	1	0.3756	0.0159		
Schnurer et al. 1985	Uppsala, central Sweden	MBC	0-20	cropland	59.51 N	5.4	570	NO_3	80	1	0.3072	0.0077		
Johnson et al. 1997	Placerville, California, USA	soil total C	0-15	forest	38.44 N	14.1	980	NO_3	100	3	-0.0802	0.005		
Johnson et al. 1997	Placerville, California, USA	soil total C	0-15	forest	38.44 N	14.1	980	NO_3	200	3	-0.0286	0.0047		
Johnson et al. 2000	Placerville, California, USA	soil respiration		forest	38.44 N	14.1	980	NO_3	100	6	0	0.1389		
Johnson et al. 2000	Placerville, California, USA	soil respiration		forest	38.44 N	14.1	980	NO_3	200	6	0.4274	0.0396		
Johnson et al. 2000	Placerville, California, USA	soil total C	0-15	forest	38.44 N	14.1	980	NO_3	100	6	0.0062	0.0119		
Johnson et al. 2000	Placerville, California, USA	soil total C	0-15	forest	38.44 N	14.1	980	NO_3	200	6	-0.0506	0.0128		

Illeris et al. 2003	NE Greenland	MBC	0-10	desert	74.30 N	-10	200	NH_4NO_3	37.5	2	-0.0408	0.009
Gallo et al. 2005	Michigan, USA	litter C		forest	44.14 N	7.2	810	NO_3	80	2	-0.0631	0.0105
Gallo et al. 2005	Michigan, USA	litter C		forest	44.15 N	7.2	810	NO_3	80	2	0.0807	0.002
Gallo et al. 2005	Michigan, USA	litter C		forest	44.16 N	7.2	810	NO_3	80	2	0.0628	0.0011
Gallo et al. 2005	Michigan, USA	soil total C	0-20	forest	44.17 N	7.2	810	NO_3	80	2	-0.1094	0.0052
Gallo et al. 2005	Michigan, USA	soil total C	0-20	forest	44.18 N	7.2	810	NO_3	80	2	-0.0577	0.067
Gallo et al. 2005	Michigan, USA	soil total C	0-20	forest	44.19 N	7.2	810	NO_3	80	2	0.0586	0.0182
Gallo et al. 2005	Michigan, USA	DOC	0-20	forest	44.20 N	7.2	810	NO_3	80	2	0.295	0.011
Gallo et al. 2005	Michigan, USA	DOC	0-20	forest	44.21 N	7.2	810	NO_3	80	2	0.2434	0.035
Gallo et al. 2005	Michigan, USA	DOC	0-20	forest	44.22 N	7.2	810	NO_3	80	2	-0.0864	0.0367
Ros et al. 2006	Linz, Austria	soil TOC	0-20	cropland	48.31 N	9.4	832	NH_4NO_3	80	12	-0.0345	0.0009
Ros et al. 2006	Linz, Austria	MBC	0-20	cropland	48.31 N	9.4	832	NH_4NO_3	80	12	0.0372	0.002
Ros et al. 2006	Linz, Austria	soil respiration		cropland	48.31 N	9.4	832	NH_4NO_3	80	12	-0.0991	0.0255
Willams & Silcock 1997	the Moidach More, Scotland, UK	MBC	0-5	wetland	57.1 N	8	800	NH_4NO_3	10	1	1.0245	0.0464
Willams & Silcock 1997	the Moidach More, Scotland, UK	MBC	0-5	wetland	57.1 N	8	800	NH_4NO_3	30	1	1.2862	0.0439
Willams & Silcock 1997	the Moidach More, Scotland, UK	MBC	0-5	wetland	57.1 N	8	800	NH_4NO_3	100	1	0.7282	0.0479
Willams & Silcock 1997	the Moidach More, Scotland, UK	MBC	5-10	wetland	57.1 N	8	800	NH_4NO_3	10	1	1.7387	0.0613
Willams & Silcock 1997	the Moidach More, Scotland, UK	MBC	5-10	wetland	57.1 N	8	800	NH_4NO_3	30	1	1.689	0.0596
Willams & Silcock 1997	the Moidach More, Scotland, UK	MBC	5-10	wetland	57.1 N	8	800	NH_4NO_3	100	1	1.2478	0.0785
Ding et al. 2007	Fengqiu County, Henan, China	soil respiration		cropland	35.00 N	13.9	615	urea	150	1	0.2872	0.0425
Ding et al. 2007	Fengqiu County, Henan, China	soil respiration		cropland	35.00 N	13.9	615	urea	250	1	0.4267	0.0873
Haile-Mariam et al. 2000	Placerville, California, USA	soil TOC	0-18	forest	38.73 N	14.1	979	NH_4	200	1	0.0197	0.0003
Haile-Mariam et al. 2000	Placerville, California, USA	soil TOC	18-30	forest	38.73 N	14.1	979	NH_4	200	1	-0.0313	0.0011
Haile-Mariam et al. 2000	Placerville, California, USA	soil TOC	30-60	forest	38.73 N	14.1	979	NH_4	200	1	-0.1416	0.0011
Liu et al. 2010a	Pingliang, Gansu, China	soil TOC	0-20	cropland	35.16 N	9.8	2834	urea	90	20	0.0198	0.0033
Liu et al. 2010a	Pingliang, Gansu, China	MBC	0-20	cropland	35.16 N	9.8	2834	urea	90	20	0.109	0.0322

Liu et al. 2010a	Pingliang, Gansu, China	soil respiration		cropland	35.16 N	9.8	2834	urea	90	20	0.0199	0.0114		
Aarnio & Martikainen 1994	southern Finland	soil respiration		forest	61.19 N	3.3	680	urea	200	3	-0.1959	0.0049		
Raiesi 2004	Shahre Kord, Iran	MBC		cropland	32.19 N	5	450	urea	150		0.4761	0.0005		
Raiesi 2004	Shahre Kord, Iran	MBC		cropland	32.19 N	5	450	urea	300		0.6867	0.0007		
Raiesi 2004	Shahre Kord, Iran	MBC		cropland	32.19 N	5	450	urea	150		0.5689	0.0039		
Raiesi 2004	Shahre Kord, Iran	MBC		cropland	32.19 N	5	450	urea	300		0.6304	0.0037		
Liu et al. 2007	Duolun County, Inner Mongolia, China	MBC	0-15	grassland	42.27 N	2.1	385	urea	150	1	-0.3314	0.0404		
Liu et al. 2007	Duolun County, Inner Mongolia, China	MBC	0-15	grassland	42.27 N	2.1	385	urea	150	1	0.0625	0.0694		
Liu et al. 2007	Duolun County, Inner Mongolia, China	microbial respiration		grassland	42.27 N	2.1	385	urea	150	1	-0.2932	0.0218		
Liu et al. 2007	Duolun County, Inner Mongolia, China	microbial respiration		grassland	42.27 N	2.1	385	urea	150	1	-0.0578	0.015		
Spinnler et al. 2002	Birmensdorf, Switzerland	soil respiration		forest	47.21 N	9.3	1130	NH_4NO_3	50	2	-0.2954	0.0067		
Matsushima & Chang 2007	Whitecourt, Alberta, Canada	litter decomposition rate		forest	54.7 N	2.6	578	urea	200	15	0.0953	0.0103	*Calamagrostis canadensis*	herbaceous
Mo et al. 2007	Guangzhou, Guangdong, China	soil respiration		forest	23.10 N	21	1927	NH_4NO_3	50	2	-0.046	0.0059		
Mo et al. 2007	Guangzhou, Guangdong, China	soil respiration		forest	23.10 N	21	1927	NH_4NO_3	100	2	-0.0792	0.006		
Mo et al. 2007	Guangzhou, Guangdong, China	litterfall		forest	23.10 N	21	1927	NH_4NO_3	50	2	-0.1325	0.0112		
Mo et al. 2007	Guangzhou, Guangdong, China	litterfall		forest	23.10 N	21	1927	NH_4NO_3	100	2	-0.0311	0.01		
Mo et al. 2007	Guangzhou, Guangdong, China	soil respiration		forest	23.10 N	21	1927	NH_4NO_3	50	2	-0.2264	0.0063		
Mo et al. 2007	Guangzhou, Guangdong, China	soil respiration		forest	23.10 N	21	1927	NH_4NO_3	100	2	-0.1068	0.0057		
Mo et al. 2007	Guangzhou, Guangdong, China	litterfall		forest	23.10 N	21	1927	NH_4NO_3	50	2	0.3386	0.0088		
Mo et al. 2007	Guangzhou, Guangdong, China	litterfall		forest	23.10 N	21	1927	NH_4NO_3	100	2	0.2653	0.0101		
Wang et al. 2010	Tshane, Zambia	soil respiration		grassland	24.17 S	17.2	365	NO_3	133	2	-0.3662	0.1239		
Wang et al. 2010	Ghanzi, Zambia	soil respiration		grassland	21.65 S	18.5	400	NO_3	133	2	0.0408	0.11		
Wang et al. 2010	Pandamatenga, Zambia	soil respiration		grassland	18.66 S	20.6	698	NO_3	133	2	0.5529	0.0088		

Wang et al. 2010	Mongu, Zambia	soil respiration		grassland	15.44 S	21.9	879	NO_3	133	2	0.8699	0.6279		
Lamb et al. 2007	Kinsella, Alberta, Canada	plant aboveground part C		grassland	53.05 N	2.5	431	NH_4NO_3	54.4	1	0.3159	0.0049		total
Lamb et al. 2007	Kinsella, Alberta, Canada	plant belowground part C		grassland	53.05 N	2.5	431	NH_4NO_3	54.4	1	0.1221	0.0099		total
Ni et al. 2012	Harbin, Heilongjiang, China	soil respiration		cropland	45.41 N	3.5	533	urea	225	1	-0.2153	0.0047		
Sifola & Postiglione 2003	Sele River Plain, Italy	plant aboveground part C		cropland	40.37 N	15.9	1006	NH_4NO_3	120	2	0.1789	0.029		herbaceous
Sifola & Postiglione 2003	Sele River Plain, Italy	plant aboveground part C		cropland	40.37 N	15.9	1006	NH_4NO_3	240	2	0.3502	0.0464		herbaceous
Sifola & Postiglione 2003	Sele River Plain, Italy	plant aboveground part C		cropland	40.37 N	15.9	1006	NH_4NO_3	360	2	0.2625	0.0443		herbaceous
Sifola & Postiglione 2003	Sele River Plain, Italy	plant aboveground part C		cropland	40.37 N	15.9	1006	NH_4NO_3	120	2	0.4065	1.0084		herbaceous
Sifola & Postiglione 2003	Sele River Plain, Italy	plant aboveground part C		cropland	40.37 N	15.9	1006	NH_4NO_3	240	2	0.5427	1.0087		herbaceous
Sifola & Postiglione 2003	Sele River Plain, Italy	plant aboveground part C		cropland	40.37 N	15.9	1006	NH_4NO_3	360	2	0.3293	1.0436		herbaceous
Bubier et al. 2007	Ottawa, Ontario, Canada	litter decomposition rate		wetland	45.40 N	6	944	NH_4NO_3	80	70	-0.0035	0.0002	*Chamaedaphne*	woody
Bubier et al. 2007	Ottawa, Ontario, Canada	litter decomposition rate		wetland	45.40 N	6	944	NH_4NO_3	80	70	0.0634	0.0158	*Sphagnum*	moss
Chiang et al. 2000	Miami, southern Florida, USA	plant aboveground part C		wetland	25.13 N	24	1559	NH_4	56	4	-0.0277	0.0382		total
Chiang et al. 2000	Miami, southern Florida, USA	plant aboveground part C		wetland	25.13 N	24	1559	NH_4	224	4	-0.306	0.0112		total
Chiang et al. 2000	Miami, southern Florida, USA	plant aboveground part C		wetland	25.13 N	24	1559	NH_4	56	4	-0.534	0.0724		total
Chiang et al. 2000	Miami, southern Florida, USA	plant aboveground part C		wetland	25.13 N	24	1559	NH_4	224	4	0.2186	0.0179		total
Corre et al. 2003	Solling, Cermany	MBC	forest floor	forest	51.44 N	7.3	1100	NH_4	140	11	-0.3414	0.0106		
Corre et al. 2003	Solling, Cermany	MBC	0-5	forest	51.44 N	7.3	1100	NH_4	140	11	-0.2102	0.0274		
Corre et al. 2003	Solling, Cermany	soil respiration		forest	51.44 N	7.3	1100	NH_4	140	11	-0.4093	0.0358		
Corre et al. 2003	Solling, Cermany	soil respiration		forest	51.44 N	7.3	1100	NH_4	140	11	-0.2436	0.1259		
Fang et al. 2006	Yucheng, Shandong, China	plant aboveground part C		cropland	36.5 N	13.3	555	urea	100	2	1.0986	0.0077		herbaceous
Fang et al. 2006	Yucheng, Shandong, China	plant aboveground part C		cropland	36.5 N	13.3	555	urea	200	2	1.1403	0.0095		herbaceous
Fang et al. 2006	Yucheng, Shandong, China	plant aboveground part C		cropland	36.5 N	13.3	555	urea	300	2	1.381	0.0108		herbaceous
Fang et al. 2006	Yucheng, Shandong, China	plant aboveground part C		cropland	36.5 N	13.3	555	urea	100	2	1.0531	0.0035		herbaceous
Fang et al. 2006	Yucheng, Shandong, China	plant aboveground part C		cropland	36.5 N	13.3	555	urea	200	2	1.2871	0.005		herbaceous
Fang et al. 2006	Yucheng, Shandong, China	plant aboveground part C		cropland	36.5 N	13.3	555	urea	300	2	1.3918	0.0332		herbaceous

Fang et al. 2006	Yucheng, Shandong, China	plant aboveground part C		cropland	36.5 N	13.3	555	urea	100	2	0.4742	0.0307	herbaceous
Fang et al. 2006	Yucheng, Shandong, China	plant aboveground part C		cropland	36.5 N	13.3	555	urea	200	2	0.5865	0.0185	herbaceous
Fang et al. 2006	Yucheng, Shandong, China	plant aboveground part C		cropland	36.5 N	13.3	555	urea	300	2	0.7844	0.0214	herbaceous
Fang et al. 2006	Yucheng, Shandong, China	plant aboveground part C		cropland	36.5 N	13.3	555	urea	100	2	0.4836	0.1333	herbaceous
Fang et al. 2006	Yucheng, Shandong, China	plant aboveground part C		cropland	36.5 N	13.3	555	urea	200	2	0.7172	0.1113	herbaceous
Fang et al. 2006	Yucheng, Shandong, China	plant aboveground part C		cropland	36.5 N	13.3	555	urea	300	2	0.8508	0.1111	herbaceous
Gnankambary et al. 2008	Dossi, Burkina Faso	soil respiration		forest	11.22 N	28	1050	urea	80	2	-0.1112	0.0062	
Gnankambary et al. 2008	Dossi, Burkina Faso	soil respiration		forest	11.22 N	28	1050	urea	80	2	0.2877	0.0511	
Gnankambary et al. 2008	Dossi, Burkina Faso	soil respiration		forest	11.22 N	28	1050	urea	80	2	0.0465	0.0043	
Gnankambary et al. 2008	Dossi, Burkina Faso	soil respiration		forest	11.22 N	28	1050	urea	80	2	0.0392	0.0031	
Harpole et al. 2007	Orange, CA, USA	plant aboveground part C		grassland	33.62 N	14	325	NO_3	100	1	0.3643	0.034	total
Harpole et al. 2007	Orange, CA, USA	plant belowground part C		grassland	33.62 N	14	325	NO_3	100	1	-0.1088	0.0765	total
Hossain et al. 1995	Canberra, Capital	MBC	0-2.5	forest	35.23 S	9.2	1150	NH_4	300	3	-0.3581	0.0023	
Hossain et al. 1995	Canberra, Capital	MBC	2.5-5	forest	35.23 S	9.2	1150	NH_4	300	3	-0.3264	0.0068	
Hossain et al. 1995	Canberra, Capital	MBC	5-10	forest	35.23 S	9.2	1150	NH_4	300	3	0	0.0073	
Hungate et al. 2007	Flagstaff, Arizona, USA	plant aboveground part C		forest	35.16 N	0.9	577	NH_4NO_3	450	1	0.5488	0.0614	total
Hungate et al. 2007	Flagstaff, Arizona, USA	soil total C	O-horizon	forest	35.16 N	0.9	577	NH_4NO_3	450	1	-0.5423	0.1786	
Hungate et al. 2007	Flagstaff, Arizona, USA	soil total C	0-5	forest	35.16 N	0.9	577	NH_4NO_3	450	1	0.1589	0.0146	
Jia et al. 2005	Yuzhong, Gansu, China	soil TOC		cropland	36.02 N	6.2	328		450	13	0.274	0.0006	
Jia et al. 2005	Yuzhong, Gansu, China	MBC		cropland	36.02 N	6.2	328		450	13	0.3834	0.003	
Johnson et al. 1994	Placerville, California, USA	soil total C	0-18	forest	38.44 N	14.1	980	NH_4	100	3	-0.0929	0.0052	
Johnson et al. 1994	Placerville, California, USA	soil total C	18-30	forest	38.44 N	14.1	980	NH_4	200	3	0.1122	0.0107	
Johnson et al. 1994	Placerville, California, USA	soil total C	0-30	forest	38.44 N	14.1	980	NH_4	100	3	-0.0116	0.003	
Johnson et al. 1994	Placerville, California, USA	soil total C	0-18	forest	38.44 N	14.1	980	NH_4	200	3	-0.0329	0.0047	
Johnson et al. 1994	Placerville, California, USA	soil total C	18-30	forest	38.44 N	14.1	980	NH_4	100	3	-0.0061	0.0055	
Johnson et al. 1994	Placerville, California, USA	soil total C	0-30	forest	38.44 N	14.1	980	NH_4	200	3	-0.0227	0.0021	

Johnson et al. 1994	Placerville, California, USA	soil respiration		forest	38.44 N	14.1	980	NH_4	100	3	0.8873	0.099		
Johnson et al. 1994	Placerville, California, USA	soil respiration		forest	38.44 N	14.1	980	NH_4	200	3	0.2513	0.1084		
Johnson et al. 2005	Sourhope, Kelso, Scotland	MBC	O-horizon	grassland	55.59 N	9.3	705	NH_4NO_3	120	2	-0.1618	0.0095		
Johnson et al. 2005	Sourhope, Kelso, Scotland	soil respiration		grassland	55.59 N	9.3	705	NH_4NO_3	120	2	-0.8043	0.0067		
Lee & Jose 2003	Santa Rosa County, FL, USA	soil respiration		forest	30.50 N	19	1600		56	2	-0.0549	0.0008		
Lee & Jose 2003	Santa Rosa County, FL, USA	soil respiration		forest	30.50 N	19	1600		112	2	-0.1704	0.0007		
Lee & Jose 2003	Santa Rosa County, FL, USA	soil respiration		forest	30.50 N	19	1600		224	2	-0.1708	0.0023		
Lee & Jose 2003	Santa Rosa County, FL, USA	soil respiration		forest	30.50 N	19	1600		56	2	-0.1066	0.0027		
Lee & Jose 2003	Santa Rosa County, FL, USA	soil respiration		forest	30.50 N	19	1600		112	2	-0.0553	0.0012		
Lee & Jose 2003	Santa Rosa County, FL, USA	soil respiration		forest	30.50 N	19	1600		224	2	-0.0066	0.0046		
Lee & Jose 2003	Santa Rosa County, FL, USA	MBC	0-10	forest	30.50 N	19	1600		56	2	-0.2446	0.014		
Lee & Jose 2003	Santa Rosa County, FL, USA	MBC	0-10	forest	30.50 N	19	1600		112	2	-0.3362	0.0078		
Lee & Jose 2003	Santa Rosa County, FL, USA	MBC	0-10	forest	30.50 N	19	1600		224	2	-0.5041	0.0619		
Lee & Jose 2003	Santa Rosa County, FL, USA	MBC	0-10	forest	30.50 N	19	1600		56	2	-0.2735	0.035		
Lee & Jose 2003	Santa Rosa County, FL, USA	MBC	0-10	forest	30.50 N	19	1600		112	2	-0.2971	0.0364		
Lee & Jose 2003	Santa Rosa County, FL, USA	MBC	0-10	forest	30.50 N	19	1600		224	2	-0.5934	0.0925		
Hobbie 2008	Cedar Creek, Minnesota, USA	litter decomposition rate		grassland	45.40 N	6.7	801	NH_4NO_3	100	70	-0.1542	0.0056	*Acer*	woody
Hobbie 2008	Cedar Creek, Minnesota, USA	litter decomposition rate		grassland	45.40 N	6.7	801	NH_4NO_3	100	70	0.0351	0.0083	*Quercus*	woody
Hobbie 2008	Cedar Creek, Minnesota, USA	litter decomposition rate		grassland	45.40 N	6.7	801	NH_4NO_3	100	70	0.071	0.0065	*Pinus*	woody
Hobbie 2008	Cedar Creek, Minnesota, USA	litter decomposition rate		grassland	45.40 N	6.7	801	NH_4NO_3	100	70	0.037	0.0056	*Schizachyrium*	herbaecous
Hobbie 2008	Cedar Creek, Minnesota, USA	litter decomposition rate		grassland	45.40 N	6.7	801	NH_4NO_3	100	70	0.0377	0.0012	*Acer*	woody
Hobbie 2008	Cedar Creek, Minnesota, USA	litter decomposition rate		grassland	45.40 N	6.7	801	NH_4NO_3	100	70	0	0.0091	*Quercus*	woody
Hobbie 2008	Cedar Creek, Minnesota, USA	litter decomposition rate		grassland	45.40 N	6.7	801	NH_4NO_3	100	70	-0.1769	0.0094	*Pinus*	woody
Hobbie 2008	Cedar Creek, Minnesota, USA	litter decomposition rate		grassland	45.40 N	6.7	801	NH_4NO_3	100	70	-0.0702	0.0194	*Schizachyrium*	herbaecous
Hobbie 2008	Cedar Creek, Minnesota, USA	litter decomposition rate		grassland	45.40 N	6.7	801	NH_4NO_3	100	70	-0.11	0.0075	*Acer*	woody
Hobbie 2008	Cedar Creek, Minnesota, USA	litter decomposition rate		grassland	45.40 N	6.7	801	NH_4NO_3	100	70	0	0.0106	*Quercus*	woody

Hobbie 2008	Cedar Creek, Minnesota, USA	litter decomposition rate		grassland	45.40 N	6.7	801	NH_4NO_3	100	70	-0.1796	0.0041	*Pinus*	woody
Hobbie 2008	Cedar Creek, Minnesota, USA	litter decomposition rate		grassland	45.40 N	6.7	801	NH_4NO_3	100	70	-0.1406	0.0081	*Schizachyrium*	herbaecous
Hobbie 2008	Cedar Creek, Minnesota, USA	litter decomposition rate		grassland	45.40 N	6.7	801	NH_4NO_3	100	70	-0.5411	0.0061	*Acer*	woody
Hobbie 2008	Cedar Creek, Minnesota, USA	litter decomposition rate		grassland	45.40 N	6.7	801	NH_4NO_3	100	70	-0.0225	0.005	*Quercus*	woody
Hobbie 2008	Cedar Creek, Minnesota, USA	litter decomposition rate		grassland	45.40 N	6.7	801	NH_4NO_3	100	70	-0.208	0.0031	*Pinus*	woody
Hobbie 2008	Cedar Creek, Minnesota, USA	litter decomposition rate		grassland	45.40 N	6.7	801	NH_4NO_3	100	70	-0.1483	0.0022	*Schizachyrium*	herbaecous
Hobbie 2008	Cedar Creek, Minnesota, USA	litter decomposition rate		grassland	45.40 N	6.7	801	NH_4NO_3	100	70	0.081	0.0091	*Acer*	woody
Hobbie 2008	Cedar Creek, Minnesota, USA	litter decomposition rate		grassland	45.40 N	6.7	801	NH_4NO_3	100	70	-0.0218	0.0124	*Quercus*	woody
Hobbie 2008	Cedar Creek, Minnesota, USA	litter decomposition rate		grassland	45.40 N	6.7	801	NH_4NO_3	100	70	0.1752	0.0104	*Pinus*	woody
Hobbie 2008	Cedar Creek, Minnesota, USA	litter decomposition rate		grassland	45.40 N	6.7	801	NH_4NO_3	100	70	0.1419	0.01	*Schizachyrium*	herbaecous
Hobbie 2008	Cedar Creek, Minnesota, USA	litter decomposition rate		grassland	45.40 N	6.7	801	NH_4NO_3	100	70	0.1345	0.0089	*Acer*	woody
Hobbie 2008	Cedar Creek, Minnesota, USA	litter decomposition rate		grassland	45.40 N	6.7	801	NH_4NO_3	100	70	-0.1931	0.0194	*Quercus*	woody
Hobbie 2008	Cedar Creek, Minnesota, USA	litter decomposition rate		grassland	45.40 N	6.7	801	NH_4NO_3	100	70	0.0362	0.0159	*Pinus*	woody
Hobbie 2008	Cedar Creek, Minnesota, USA	litter decomposition rate		grassland	45.40 N	6.7	801	NH_4NO_3	100	70	0.0316	0.0122	*Schizachyrium*	herbaecous
Hobbie 2008	Cedar Creek, Minnesota, USA	litter decomposition rate		grassland	45.40 N	6.7	801	NH_4NO_3	100	70	-0.1027	0.0049	*Acer*	woody
Hobbie 2008	Cedar Creek, Minnesota, USA	litter decomposition rate		grassland	45.40 N	6.7	801	NH_4NO_3	100	70	-0.0345	0.012	*Quercus*	woody
Hobbie 2008	Cedar Creek, Minnesota, USA	litter decomposition rate		grassland	45.40 N	6.7	801	NH_4NO_3	100	70	-0.0834	0.0116	*Pinus*	woody
Hobbie 2008	Cedar Creek, Minnesota, USA	litter decomposition rate		grassland	45.40 N	6.7	801	NH_4NO_3	100	70	-0.0911	0.0027	*Schizachyrium*	herbaecous
Hobbie 2008	Cedar Creek, Minnesota, USA	litter decomposition rate		grassland	45.40 N	6.7	801	NH_4NO_3	100	70	-0.2162	0.0191	*Acer*	woody
Hobbie 2008	Cedar Creek, Minnesota, USA	litter decomposition rate		grassland	45.40 N	6.7	801	NH_4NO_3	100	70	-0.0953	0.0062	*Quercus*	woody
Hobbie 2008	Cedar Creek, Minnesota, USA	litter decomposition rate		grassland	45.40 N	6.7	801	NH_4NO_3	100	70	-0.1692	0.0063	*Pinus*	woody
Hobbie 2008	Cedar Creek, Minnesota, USA	litter decomposition rate		grassland	45.40 N	6.7	801	NH_4NO_3	100	70	0.0162	0.0023	*Schizachyrium*	herbaecous
Fornara et al. 2013	Silwood Park, Berkshire, UK	soil total C	0-20	grassland	51.41 N	11.5	602	NH_4NO_3	100	19	0.2941	0.0005		
Fornara et al. 2013	Silwood Park, Berkshire, UK	plant aboveground part C		grassland	51.42 N	11.5	602	NH_4NO_3	100	19	0.3247	0.0005		
Fornara et al. 2013	Silwood Park, Berkshire, UK	plant belowground part C		grassland	51.43 N	11.5	602	NH_4NO_3	100	19	0.2539	0.0029		
Fornara & Tilman 2012	Minnesota, USA	soil total C	0-20	grassland	45.24 N	6	810	NH_4NO_3	10	27	0.1282	0.0002		

Fornara & Tilman 2012	Minnesota, USA	soil total C	0-20	grassland	45.24 N	6	810	NH_4NO_3	20	27	0.1284	0.0001
Fornara & Tilman 2012	Minnesota, USA	soil total C	0-20	grassland	45.24 N	6	810	NH_4NO_3	34	27	0.2548	0.0002
Fornara & Tilman 2012	Minnesota, USA	soil total C	0-20	grassland	45.24 N	6	810	NH_4NO_3	54	27	0.2118	0.0002
Fornara & Tilman 2012	Minnesota, USA	soil total C	0-20	grassland	45.24 N	6	810	NH_4NO_3	95	27	0.3714	0.0001
Fornara & Tilman 2012	Minnesota, USA	soil total C	0-20	grassland	45.24 N	6	810	NH_4NO_3	170	27	0.3648	0.0001
Fornara & Tilman 2012	Minnesota, USA	soil total C	0-20	grassland	45.24 N	6	810	NH_4NO_3	270	27	0.3585	0.0001

PFT: plant functional type

Supplementary Table S3 References list for the primary studies used in the meta-analysis

Aarnio T, Martikainen PJ (1994) Mineralization of carbon and nitrogen in acid forest soil treated with fast and slow-release nutrients. Plant and soil, **164**, 187-193.

Aerts R, De Caluwe H, Beltman B (2003) Plant community mediated vs. nutritional controls on litter decomposition rates in grasslands. Ecology, **84**, 3198-3208.

Aerts R, Van Logtestijn R, Karlsson P (2006) Nitrogen supply differentially affects litter decomposition rates and nitrogen dynamics of sub-arctic bog species. Oecologia, **146**, 652-658.

Allard V, Robin C, Newton P, Lieffering M, Soussana J (2006) Short and long-term effects of elevated CO 2 on Lolium perenne rhizodeposition and its consequences on soil organic matter turnover and plant N yield. Soil Biology and Biochemistry, **38**, 1178-1187.

Ambus P, Robertson G (2006) The effect of increased N deposition on nitrous oxide, methane and carbon dioxide fluxes from unmanaged forest and grassland communities in Michigan. Biogeochemistry, **79**, 315-337.

Ares A, Fownes JH (2001) Productivity, resource use, and competitive interactions of Fraxinus uhdei in Hawaii uplands. Canadian Journal of Forest Research, **31**, 132-142.

Augustine DJ, Mcnaughton SJ, Frank DA (2003) Feedbacks between soil nutrients and large herbivores in a managed savanna ecosystem. Ecological Applications, **13**, 1325-1337.

Aydin I, Uzun F (2005) Nitrogen and phosphorus fertilization of rangelands affects yield, forage quality and the botanical composition. European Journal of Agronomy, **23**, 8-14.

Báez S, Fargione J, Moore D, Collins S, Gosz J (2007) Atmospheric nitrogen deposition in the northern Chihuahuan desert: temporal trends and potential consequences. Journal of Arid Environments, **68**, 640-651.

Baer S, Blair J, Collins S, Knapp A (2003) Soil resources regulate productivity and diversity in newly established tallgrass prairie. Ecology, **84**, 724-735.

Baer SG, Blair JM (2008) Grassland establishment under varying resource availability: a test of positive and negative feedback. Ecology, **89**, 1859-1871.

Balík J, Cerny J, Tlustos P, Zitková M (2003) Nitrogen balance and mineral nitrogen content in the soil in a long experiment with maize under different systems of N fertilization. Plant Soil and Environment, **49**, 554-559.

Barger NN, D'antonio CM, Ghneim T, Brink K, Cuevas E (2002) Nutrient Limitation to Primary Productivity in a Secondary Savanna in Venezuela1. Biotropica, **34**, 493-501.

Barger NN, D'antonio CM, Ghneim T, Cuevas E (2003) Constraints to colonization and growth of the African grass, Melinis minutiflora, in a Venezuelan savanna. Plant Ecology, **167**, 31-43.

Barnard R, Le Roux X, Hungate B, Cleland E, Blankinship J, Barthes L, Leadley P (2006) Several components of global change alter nitrifying and denitrifying activities in an annual grassland. Functional Ecology, **20**, 557-564.

Basiliko N, Khan A, Prescott CE, Roy R, Grayston SJ (2009) Soil greenhouse gas and nutrient dynamics in fertilized western Canadian plantation forests. Canadian Journal of Forest Research, **39**, 1220-1235.

Bechtold H, Inouye R (2007) Distribution of carbon and nitrogen in sagebrush steppe after six years of nitrogen addition and shrub removal. Journal of Arid Environments, **71**, 122-132.

Bejarano M, Crosby MM, Parra V, Etchevers JD, Campo J (2014) Precipitation Regime and Nitrogen Addition Effects on Leaf Litter Decomposition in Tropical Dry Forests. Biotropica, **46**, 415-424.

Bennett LT, Adams MA (2001) Response of a perennial grassland to nitrogen and phosphorus additions in sub-tropical, semi-arid Australia. Journal of Arid Environments, **48**, 289-308.

Bobbink R (1991) Effects of Nutrient Enrichment in Dutch Chalk Grassland. Journal of Applied Ecology, **28**, 28-41.

Boeye D, Verhagen B, Van Haesebroeck V, Verheyen RF (1997) Nutrient limitation in species ‐ rich lowland fens. Journal of Vegetation Science, **8**, 415-424.

Bonanomi G, Caporaso S, Allegrezza M (2006) Short-term effects of nitrogen enrichment, litter removal and cutting on a Mediterranean grassland. Acta Oecologica, **30**, 419-425.

Bowden RD, Davidson E, Savage K, Arabia C, Steudler P (2004) Chronic nitrogen additions reduce total soil respiration and microbial respiration in temperate forest soils at the Harvard Forest. Forest Ecology and Management, **196**, 43-56.

Bowman WD, Theodose TA, Schardt JC, Conant RT (1993) Constraints of nutrient availability on primary production in two alpine tundra communities. Ecology, **74**, 2085-2097.

Bradley K, Drijber RA, Knops J (2006) Increased N availability in grassland soils modifies their microbial communities and decreases the abundance of arbuscular mycorrhizal fungi. Soil Biology and Biochemistry, **38**, 1583-1595.

Bragazza L, Buttler A, Habermacher J *et al.* (2012) High nitrogen deposition alters the decomposition of bog plant litter and reduces carbon accumulation. Global Change Biology, **18**, 1163-1172.

Brenner RE, Boone RD, Ruess RW (2005) Nitrogen additions to pristine, high-latitude, forest ecosystems: consequences for soil nitrogen transformations and retention in mid and late succession. Biogeochemistry, **72**, 257-282.

Bubier JL, Moore TR, Bledzki LA (2007) Effects of nutrient addition on vegetation and carbon cycling in an ombrotrophic bog. Global Change Biology, **13**, 1168-1186.

Burton AJ, Pregitzer KS, Crawford JN, Zogg GP, Zak DR (2004) Simulated chronic NO3− deposition reduces soil respiration in northern hardwood forests. Global Change Biology, **10**, 1080-1091.

Camill P, Mckone MJ, Sturges ST *et al.* (2004) Community-and ecosystem-level changes in a species-rich tallgrass prairie restoration. Ecological Applications, **14**, 1680-1694.

Campo J, Vázquez-Yanes C (2004) Effects of nutrient limitation on aboveground carbon dynamics during tropical dry forest regeneration in Yucatan, Mexico. Ecosystems, **7**, 311-319.

Carpenter AT, Moore JC, Redente EF, Stark JC (1990) Plant community dynamics in a semi-arid ecosystem in relation to nutrient addition following a major disturbance. Plant and soil, **126**, 91-99.

Carreiro M, Sinsabaugh R, Repert D, Parkhurst D (2000) Microbial enzyme shifts explain litter decay responses to simulated nitrogen deposition. Ecology, **81**, 2359-2365.

Chen C, Xu Z, Hughes J (2002) Effects of nitrogen fertilization on soil nitrogen pools and microbial properties in a hoop pine (Araucaria cunninghamii) plantation in southeast Queensland, Australia. Biology and fertility of soils, **36**, 276-283.

Chen X, Li Y, Mo J *et al.* (2012a) Effects of nitrogen deposition on soil organic carbon fractions in the subtropical forest ecosystems of S China. Journal of Plant Nutrition and Soil Science, **175**, 947-953.

Chen X, Liu J, Deng Q, Yan J, Zhang D (2012b) Effects of elevated CO2 and nitrogen addition on soil organic carbon fractions in a subtropical forest. Plant and soil, **357**, 25-34.

Chiang C, Craft CB, Rogers DW, Richardson CJ (2000) Effects of 4 years of nitrogen and phosphorus additions on Everglades plant communities. Aquatic botany, **68**, 61-78.

Churchland C, Mayo-Bruinsma L, Ronson A, Grogan P (2010) Soil microbial and plant community responses to single large carbon and nitrogen additions in low arctic tundra. Plant and soil, **334**, 409-421.

Cleveland CC, Townsend AR (2006) Nutrient additions to a tropical rain forest drive substantial soil carbon dioxide losses to the atmosphere. Proceedings of the National Academy of Sciences, **103**, 10316-10321.

Compton JE, Watrud LS, Arlene Porteous L, Degrood S (2004) Response of soil microbial biomass and community composition to chronic nitrogen additions at Harvard forest. Forest Ecology and Management, **196**, 143-158.

Corre MD, Beese FO, Brumme R (2003) Soil nitrogen cycle in high nitrogen deposition forest: changes under nitrogen saturation and liming. Ecological Applications, **13**, 287-298.

Cusack DF, Silver WL, Torn MS, Mcdowell WH (2011) Effects of nitrogen additions on above-and belowground carbon dynamics in two tropical forests. Biogeochemistry, **104**, 203-225.

Cusack DF, Torn MS, Mcdowell WH, Silver WL (2010) The response of heterotrophic activity and carbon cycling to nitrogen additions and warming in two tropical soils. Global Change Biology, **16**, 2555-2572.

D'antonio CM, Mack MC (2006) Nutrient Limitation in a Fire‐derived, Nitrogen‐rich Hawaiian Grassland1. Biotropica, **38**, 458-467.

Davidson EA, Reis De Carvalho CJ, Vieira IC *et al.* (2004) Nitrogen and phosphorus limitation of biomass growth in a tropical secondary forest. Ecological Applications, **14**, 150-163.

Davis MR, Allen RB, Clinton PW (2004) The influence of N addition on nutrient content, leaf carbon isotope ratio, and productivity in a Nothofagus forest during stand development. Canadian Journal of Forest Research, **34**, 2037-2048.

Deng Q, Cheng X, Zhou G, Liu J, Liu S, Zhang Q, Zhang D (2013) Seasonal responses of soil respiration to elevated CO 2 and N addition in young subtropical forest ecosystems in southern China. Ecological engineering, **61**, 65-73.

Deng Q, Zhou G, Liu J, Liu S, Duan H, Zhang D (2010) Responses of soil respiration to elevated carbon dioxide and nitrogen addition in young subtropical forest ecosystems in China. Biogeosciences, **7**, 315-328.

Dijkstra FA, Hobbie SE, Reich PB, Knops JM (2005) Divergent effects of elevated CO2, N fertilization, and plant diversity on soil C and N dynamics in a grassland field experiment. Plant and soil, **272**, 41-52.

Ding W, Cai Y, Cai Z, Yagi K, Zheng X (2007) Soil respiration under maize crops: effects of water, temperature, and nitrogen fertilization. Soil Science Society of America Journal, **71**, 944-951.

Dou JX, Liu JS, Wang Y, Zhao GY (2008) Effects of simulated nitrogen deposition on biomass of wetland plant and soil active carbon pool. Ying yong sheng tai xue bao, **19**, 1714-1720.

Du Y, Guo P, Liu J, Wang C, Yang N, Jiao Z (2014a) Different types of nitrogen deposition show variable effects on the soil carbon cycle process of temperate forests. Global Change Biology, **20**, 3222-3228.

Du Z, Wang W, Zeng W, Zeng H (2014b) Nitrogen Deposition Enhances Carbon Sequestration by Plantations in Northern China. PloS one, **9**, e87975.

Dukes JS, Chiariello NR, Cleland EE *et al.* (2005) Responses of grassland production to single and multiple global environmental changes. PLoS biology, **3**, e319.

Fan H, Yuan Y, Wang Q, Li Y, Huang R (2007) Effects of nitrogen deposition on soil organic carbon and total nitrogen beneath Chinese fir plantations. Fu Jian Lin Xue Yuan Xue Bao, **27**, 1-6.

Fang H, Cheng S, Yu G *et al.* (2014) Experimental nitrogen deposition alters the quantity and quality of soil dissolved organic carbon in an alpine meadow on the Qinghai-Tibetan Plateau. Applied Soil Ecology, **81**, 1-11.

Fang Q, Yu Q, Wang E, Chen Y, Zhang G, Wang J, Li L (2006) Soil nitrate accumulation, leaching and crop nitrogen use as influenced by fertilization and irrigation in an intensive wheat–maize double cropping system in the North China Plain. Plant and soil, **284**, 335-350.

Fang X, Liu J, Zhang D *et al.* (2012) Effects of precipitation change and nitrogen addition on organic carbon mineralization and soil microbial carbon of the forest soils in Dinghushan, southeastern China. Ying yong Yu Huan Jing Sheng Wu Xue Bao, **18**, 531-538.

Fornara DA, Banin L, Crawley M (2013) Multi-nutrient vs. nitrogen-only effects on carbon sequestration in grassland soils. Global Change Biology, 2013 **19**, 3848-3857.

Fornara DA, Tilman D (2012) Soil carbon sequestration in praire grasslands increased by chronic nitrogen addition. Ecology, 2012 **93**, 2030-2036.

Frost JW, Schleicher T, Craft C (2009) Effects of nitrogen and phosphorus additions on primary production and invertebrate densities in a Georgia (USA) tidal freshwater marsh. Wetlands, **29**, 196-203.

Gallo M, Lauber C, Cabaniss S, Waldrop M, Sinsabaugh R, Zak DR (2005) Soil organic matter and litter chemistry response to experimental N deposition in northern temperate deciduous forest ecosystems. Global Change Biology, **11**, 1514-1521.

Gnankambary Z, Ilstedt U, Nyberg G, Hien V, Malmer A (2008) Nitrogen and phosphorus limitation of soil microbial respiration in two tropical agroforestry parklands in the south-Sudanese zone of Burkina Faso: the effects of tree canopy and fertilization. Soil Biology and Biochemistry, **40**, 350-359.

Gong S, Guo R, Zhang T, Guo J (2015) Warming and Nitrogen Addition Increase Litter Decomposition in a Temperate Meadow Ecosystem. PloS one, **10**.

Gough L, Hobbie SE (2003) Responses of moist non‐acidic arctic tundra to altered environment: productivity, biomass, and species richness. Oikos, **103**, 204-216.

Green EK, Galatowitsch SM (2002) Effects of Phalaris arundinacea and nitrate‐N addition on the establishment of wetland plant communities. Journal of Applied Ecology, **39**, 134-144.

Gulledge J, Schimel JP (2000) Controls on soil carbon dioxide and methane fluxes in a variety of taiga forest stands in interior Alaska. Ecosystems, **3**, 269-282.

Gundale MJ, From F, Bach LH, Nordin A (2014) Anthropogenic nitrogen deposition in boreal forests has a minor impact on the global carbon cycle. Global Change Biology, **20**, 276-286.

Gunnarsson U, Rydin H (2000) Nitrogen fertilization reduces Sphagnum production in bog communities. New Phytologist, **147**, 527-537.

Haag RW (1974) Nutrient limitations to plant production in two tundra communities. Canadian Journal of Botany, **52**, 103-116.

Haile-Mariam S, Cheng W, Johnson D, Ball J, Paul E (2000) Use of carbon-13 and carbon-14 to measure the effects of carbon dioxide and nitrogen fertilization on carbon dynamics in ponderosa pine. Soil Science Society of America Journal, **64**, 1984-1993.

Han Y, Zhang Z, Wang C, Jiang F, Xia J (2012) Effects of mowing and nitrogen addition on soil respiration in three patches in an oldfield grassland in Inner Mongolia. Journal of Plant Ecology, **5**, 219-228.

Harpole WS, Potts DL, Suding KN (2007) Ecosystem responses to water and nitrogen amendment in a California grassland. Global Change Biology, **13**, 2341-2348.

Harrington RA, Fownes JH, Vitousek PM (2001) Production and resource use efficiencies in N-and P-limited tropical forests: a comparison of responses to long-term fertilization. Ecosystems, **4**, 646-657.

Hasselquist NJ, Metcalfe DB, Högberg P (2012) Contrasting effects of low and high nitrogen additions on soil CO2 flux components and ectomycorrhizal fungal sporocarp production in a boreal forest. Global Change Biology, **18**, 3596-3605.

Hati KM, Swarup A, Dwivedi A, Misra A, Bandyopadhyay K (2007) Changes in soil physical properties and organic carbon status at the topsoil horizon of a vertisol of central India after 28 years of continuous cropping, fertilization and manuring. Agriculture, Ecosystems & Environment, **119**, 127-134.

Heijmans MM, Berendse F, Arp WJ, Masselink AK, Klees H, De Visser W, Van Breemen N (2001) Effects of elevated carbon dioxide and increased nitrogen deposition on bog vegetation in the Netherlands. Journal of Ecology, **89**, 268-279.

Herbert DA, Fownes JH (1995) Phosphorus limitation of forest leaf area and net primary production on a highly weathered soil. Biogeochemistry, **29**, 223-235.

Hobbie SE (2000) Interactions between litter lignin and nitrogenitter lignin and soil nitrogen availability during leaf litter decomposition in a Hawaiian montane forest. Ecosystems, **3**, 484-494.

Hobbie SE (2008) Nitrogen effects on decomposition: a five-year experiment in eight temperate sites. Ecology, **89**, 2633-2644.

Hoek D, Mierlo Anita J, Groenendael JM (2004) Nutrient limitation and nutrient‐driven shifts in plant species composition in a species‐rich fen meadow. Journal of Vegetation Science, **15**, 389-396.

Hoosbeek MR, Van Breemen N, Vasander H, Buttler A, Berendse F (2002) Potassium limits potential growth of bog vegetation under elevated atmospheric CO2 and N deposition. Global

Change Biology, **8**, 1130-1138.

Hossain A, Raison R, Khanna P (1995) Effects of fertilizer application and fire regime on soil microbial biomass carbon and nitrogen, and nitrogen mineralization in an Australian subalpine eucalypt forest. Biology and fertility of soils, **19**, 246-252.

Hu Y-L, Zeng D-H, Liu Y-X, Zhang Y-L, Chen Z-H, Wang Z-Q (2010) Responses of soil chemical and biological properties to nitrogen addition in a Dahurian larch plantation in Northeast China. Plant and soil, **333**, 81-92.

Huang Z, Clinton PW, Baisden WT, Davis MR (2011) Long-term nitrogen additions increased surface soil carbon concentration in a forest plantation despite elevated decomposition. Soil Biology and Biochemistry, **43**, 302-307.

Hungate BA, Hart SC, Selmants PC, Boyle SI, Gehring CA (2007) Soil responses to management, increased precipitation, and added nitrogen in ponderosa pine forests. Ecological Applications, **17**, 1352-1365.

Illeris L, Michelsen A, Jonasson S (2003) Soil plus root respiration and microbial biomass following water, nitrogen, and phosphorus application at a high arctic semi desert. Biogeochemistry, **65**, 15-29.

Iversen CM, Bridgham SD, Kellogg LE (2010) Scaling plant nitrogen use and uptake efficiencies in response to nutrient addition in peatlands. Ecology, **91**, 693-707.

Iversen CM, Norby RJ (2008) Nitrogen limitation in a sweetgum plantation: implications for carbon allocation and storage. Canadian Journal of Forest Research, **38**, 1021-1032.

Jia GM, Cao J, Wang G (2005) Influence of land management on soil nutrients and microbial biomass in the central Loess Plateau, Northwest China. Land Degradation & Development, **16**, 455-462.

Jia X, Shao MA, Wei X (2012) Responses of soil respiration to N addition, burning and clipping in temperate semiarid grassland in northern China. Agricultural and Forest Meteorology, **166**, 32-40.

Johnson D, Ball J, Walker R (1997) Effects of CO2 and nitrogen fertilization on vegetation and soil nutrient content in juvenile ponderosa pine. Plant and soil, **190**, 29-40.

Johnson D, Cheng W, Ball J (2000) Effects of [CO2] and nitrogen fertilization on soils planted with ponderosa pine. Plant and soil, **224**, 99-113.

Johnson D, Geisinger D, Walker R, Newman J, Vose J, Elliot K, Ball T (1994) Soil pCO2, soil respiration, and root activity in CO2-fumigated and nitrogen-fertilized ponderosa pine. Plant and soil, **165**, 129-138.

Johnson D, Hoylman A, Ball J, Walker R (2006) Ponderosa Pine Responses to Elevated CO2and Nitrogen Fertilization. Biogeochemistry, **77**, 157-175.

Johnson D, Leake J, Read D (2005) Liming and nitrogen fertilization affects phosphatase activities, microbial biomass and mycorrhizal colonisation in upland grassland. Plant and soil, **271**, 157-164.

Jones S, Rees R, Kosmas D, Ball B, Skiba U (2006) Carbon sequestration in a temperate grassland; management and climatic controls. Soil use and management, **22**, 132-142.

Jourdan C, Silva E, Gonçalves J, Ranger J, Moreira R, Laclau J-P (2008) Fine root production and turnover in Brazilian Eucalyptus plantations under contrasting nitrogen fertilization regimes.

Forest Ecology and Management, **256**, 396-404.

Keeler BL, Hobbie SE, Kellogg LE (2009) Effects of long-term nitrogen addition on microbial enzyme activity in eight forested and grassland sites: implications for litter and soil organic matter decomposition. Ecosystems, **12**, 1-15.

Ket WA, Schubauer-Berigan JP, Craft CB (2011) Effects of five years of nitrogen and phosphorus additions on a Zizaniopsis miliacea tidal freshwater marsh. Aquatic botany, **95**, 17-23.

Koehler B, Corre MD, Veldkamp E, Sueta J (2009a) Chronic nitrogen addition causes a reduction in soil carbon dioxide efflux during the high stem-growth period in a tropical montane forest but no response from a tropical lowland forest on a decadal time scale. Biogeosciences, **6**, 2973-2983.

Koehler B, Corre MD, Veldkamp E, Wullaert H, Wright SJ (2009b) Immediate and long - term nitrogen oxide emissions from tropical forest soils exposed to elevated nitrogen input. Global Change Biology, **15**, 2049-2066.

Lamb EG, Shore BH, Cahill JF (2007) Water and nitrogen addition differentially impact plant competition in a native rough fescue grassland. Plant Ecology, **192**, 21-33.

Lee K-H, Jose S (2003) Soil respiration, fine root production, and microbial biomass in cottonwood and loblolly pine plantations along a nitrogen fertilization gradient. Forest Ecology and Management, **185**, 263-273.

Li X, Zheng X, Han S, Zheng J, Li T (2010) Effects of nitrogen additions on nitrogen resorption and use efficiencies and foliar litterfall of six tree species in a mixed birch and poplar forest, northeastern China. Canadian Journal of Forest Research, **40**, 2256-2261.

Liang C, Balser TC (2012) Warming and nitrogen deposition lessen microbial residue contribution to soil carbon pool. Nature communications, **3**, 1222.

Lin GG, Zhao Q, Zhao L, Li HC, Zeng DH (2012) Effects of understory removal and nitrogen addition on the soil chemical and biological properties of Pinus sylvestris var. mongolica plantation in Keerqin Sandy Land. Ying yong sheng tai xue bao, **23**, 1188-1194.

Liu E, Yan C, Mei X *et al.* (2010a) Long-term effect of chemical fertilizer, straw, and manure on soil chemical and biological properties in northwest China. Geoderma, **158**, 173-180.

Liu J, Xu Z, Zhang D *et al.* (2011) Effects of carbon dioxide enrichment and nitrogen addition on inorganic carbon leaching in subtropical model forest ecosystems. Ecosystems, **14**, 683-697.

Liu JX, Zhou GY, Zhang DQ, Xu ZH, Duan HL, Deng Q, Zhao L (2010b) Carbon dynamics in subtropical forest soil: effects of atmospheric carbon dioxide enrichment and nitrogen addition. Journal of Soils and Sediments, **10**, 730-738.

Liu K, Crowley D (2009) Nitrogen deposition effects on carbon storage and fungal: bacterial ratios in coastal sage scrub soils of southern California. Journal of Environmental quality, **38**, 2267-2272.

Liu P, Huang J, Han X, Sun OJ, Zhou Z (2006) Differential responses of litter decomposition to increased soil nutrients and water between two contrasting grassland plant species of Inner Mongolia, China. Applied Soil Ecology, **34**, 266-275.

Liu W, Xu W, Han Y, Wang C, Wan S (2007) Responses of microbial biomass and respiration of soil to topography, burning, and nitrogen fertilization in a temperate steppe. Biology and fertility of soils, **44**, 259-268.

Liu W, Xu W, Hong J, Wan S (2010c) Interannual variability of soil microbial biomass and respiration in responses to topography, annual burning and N addition in a semiarid temperate steppe. Geoderma, **158**, 259-267.

Long FL, Li YY, Fang X, Huang WJ, Liu SE, Liu JX (2014) Effects of elevated CO2 concentration and nitrogen addition on soil carbon stability in southern subtropical experimental forest ecosystems. Zhi Wu Sheng Tai Xue Bao, **38**, 1053-1063.

Lovelock CE, Feller IC, Ellis J, Schwarz AM, Hancock N, Nichols P, Sorrell B (2007) Mangrove growth in New Zealand estuaries: the role of nutrient enrichment at sites with contrasting rates of sedimentation. Oecologia, **153**, 633-641.

Lovett GM, Arthur MA, Weathers KC, Fitzhugh RD, Templer PH (2013) Nitrogen addition increases carbon storage in soils, but not in trees, in an eastern US deciduous forest. Ecosystems, **16**, 980-1001.

Lu X, Gilliam FS, Yu G, Li L, Mao Q, Chen H, Mo J (2013) Long-term nitrogen addition decreases carbon leaching in a nitrogen-rich forest ecosystem. Biogeosciences, **10**, 3931-3941.

Ludwig F, Kroon H, Prins HH, Berendse F (2001) Effects of nutrients and shade on tree - grass interactions in an East African savanna. Journal of Vegetation Science, **12**, 579-588.

Lugato E, Berti A, Giardini L (2006) Soil organic carbon (SOC) dynamics with and without residue incorporation in relation to different nitrogen fertilisation rates. Geoderma, **135**, 315-321.

Mäkipää R (1995) Effect of nitrogen input on carbon accumulation of boreal forest soils and ground vegetation. Forest Ecology and Management, **79**, 217-226.

Mack MC, Schuur EA, Bret-Harte MS, Shaver GR, Chapin FS (2004) Ecosystem carbon storage in arctic tundra reduced by long-term nutrient fertilization. Nature, **431**, 440-443.

Magill AH, Aber JD, Currie WS *et al.* (2004) Ecosystem response to 15 years of chronic nitrogen additions at the Harvard Forest LTER, Massachusetts, USA. Forest Ecology and Management, **196**, 7-28.

Maljanen M, Jokinen H, Saari A, Strömmer R, Martikainen P (2006) Methane and nitrous oxide fluxes, and carbon dioxide production in boreal forest soil fertilized with wood ash and nitrogen. Soil use and management, **22**, 151-157.

Matsushima M, Chang SX (2007) Effects of understory removal, N fertilization, and litter layer removal on soil N cycling in a 13-year-old white spruce plantation infested with Canada bluejoint grass. Plant and soil, **292**, 243-258.

Mcdowell WH, Magill AH, Aitkenhead-Peterson JA, Aber JD, Merriam JL, Kaushal SS (2004) Effects of chronic nitrogen amendment on dissolved organic matter and inorganic nitrogen in soil solution. Forest Ecology and Management, **196**, 29-41.

Mcmaster G, Jow W, Kummerow J (1982) Response of Adenostoma fasciculatum and Ceanothus greggii chaparral to nutrient additions. The Journal of Ecology, 745-756.

Micks P, Aber JD, Boone RD, Davidson EA (2004) Short-term soil respiration and nitrogen immobilization response to nitrogen applications in control and nitrogen-enriched temperate forests. Forest Ecology and Management, **196**, 57-70.

Mirmanto E, Proctor J, Green J, Nagy L (1999) Effects of nitrogen and phosphorus fertilization in a lowland evergreen rainforest. Philosophical Transactions of the Royal Society B: Biological Sciences, **354**, 1825-1829.

Mo J, Zhang W, Zhu W, Fang Y, Li D, Zhao P (2007) Response of soil respiration to simulated N deposition in a disturbed and a rehabilitated tropical forest in southern China. Plant and soil, **296**, 125-135.

Mo J, Zhang W, Zhu W, Gundersen P, Fang Y, Li D, Wang H (2008) Nitrogen addition reduces soil respiration in a mature tropical forest in southern China. Global Change Biology, **14**, 403-412.

Ni K, Ding W, Cai Z, Wang Y, Zhang X, Zhou B (2012) Soil carbon dioxide emission from intensively cultivated black soil in Northeast China: nitrogen fertilization effect. Journal of Soils and Sediments, **12**, 1007-1018.

Niu S, Yang H, Zhang Z *et al.* (2009) Non-additive effects of water and nitrogen addition on ecosystem carbon exchange in a temperate steppe. Ecosystems, **12**, 915-926.

Nohrstedt H-, Arnebrant K, Bååth E, Söderström B (1989) Changes in carbon content, respiration rate, ATP content, and microbial biomass in nitrogen-fertilized pine forest soils in Sweden. Canadian Journal of Forest Research, **19**, 323-328.

Nowinski NS, Trumbore SE, Jimenez G, Fenn ME (2009) Alteration of belowground carbon dynamics by nitrogen addition in southern California mixed conifer forests. Journal of Geophysical Research: Biogeosciences, **114**, G02005.

ien DI (2004) Nutrient limitation in boreal rich‐fen vegetation: A fertilization experiment. Applied Vegetation Science, **7**, 119-132.

Persson H, Ahlström K (1990) The effects of forest liming on fertilization on fine-root growth. Water, Air, and Soil Pollution, **54**, 365-375.

Pregitzer KS, Burton AJ, Zak DR, Talhelm AF (2008) Simulated chronic nitrogen deposition increases carbon storage in Northern Temperate forests. Global Change Biology, **14**, 142-153.

Pregitzer KS, Zak DR, Burton AJ, Ashby JA, Macdonald NW (2004) Chronic nitrate additions dramatically increase the export of carbon and nitrogen from northern hardwood ecosystems. Biogeochemistry, **68**, 179-197.

Priess J, Fölster H (2001) Microbial properties and soil respiration in submontane forests of Venezuelian Guyana: characteristics and response to fertilizer treatments. Soil Biology and Biochemistry, **33**, 503-509.

Quang Q, Zhang Z, He NP, Shu HX, Wen XF, Sun XM (2015) Short-term effects of nitrogen addition on soil respiration of three temperate forests in Dongling Mountain. Sheng Tai xue Za Zhi, **34**, 797-804.

Raiesi F (2004) Soil properties and N application effects on microbial activities in two winter wheat cropping systems. Biology and fertility of soils, **40**, 88-92.

Reid JP, Adair EC, Hobbie SE, Reich PB (2012) Biodiversity, nitrogen deposition, and CO2 affect grassland soil carbon cycling but not storage. Ecosystems, **15**, 580-590.

Ros M, Klammer S, Knapp B, Aichberger K, Insam H (2006) Long‐term effects of compost amendment of soil on functional and structural diversity and microbial activity. Soil use and management, **22**, 209-218.

Sardans J, Peñuelas J, Rodà F (2006) The effects of nutrient availability and removal of competing vegetation on resprouter capacity and nutrient accumulation in the shrub Erica multiflora. Acta Oecologica, **29**, 221-232.

Schaeffer S, Billings S, Evans R (2003) Responses of soil nitrogen dynamics in a Mojave Desert ecosystem to manipulations in soil carbon and nitrogen availability. Oecologia, **134**, 547-553.

Schmidt S, Lipson D, Ley R, Fisk M, West A (2004) Impacts of chronic nitrogen additions vary seasonally and by microbial functional group in tundra soils. Biogeochemistry, **69**, 1-17.

Schnürer J, Clarholm M, Rosswall T (1985) Microbial biomass and activity in an agricultural soil with different organic matter contents. Soil Biology and Biochemistry, **17**, 611-618.

Sifola M, Postiglione L (2003) The effect of nitrogen fertilization on nitrogen use efficiency of irrigated and non-irrigated tobacco (Nicotiana tabacum L.). Plant and soil, **252**, 313-323.

Sinsabaugh R, Zak D, Gallo M, Lauber C, Amonette R (2004) Nitrogen deposition and dissolved organic carbon production in northern temperate forests. Soil Biology and Biochemistry, **36**, 1509-1515.

Smaill SJ, Clinton P, Greenfield L (2008) Nitrogen fertiliser effects on litter fall, FH layer and mineral soil characteristics in New Zealand Pinus radiata plantations. Forest Ecology and Management, **256**, 564-569.

Song C, Liu D, Song Y, Mao R (2013a) Effect of nitrogen addition on soil organic carbon in freshwater marsh of Northeast China. Environmental earth sciences, **70**, 1653-1659.

Song C, Liu D, Yang G, Song Y, Mao R (2011) Effect of nitrogen addition on decomposition of Calamagrostis angustifolia litters from freshwater marshes of Northeast China. Ecological engineering, **37**, 1578-1582.

Song C, Wang L, Tian H *et al.* (2013b) Effect of continued nitrogen enrichment on greenhouse gas emissions from a wetland ecosystem in the Sanjiang Plain, Northeast China: A 5 year nitrogen addition experiment. Journal of Geophysical Research: Biogeosciences, **118**, 741-751.

Song Y, Song C, Li Y, Hou C, Yang G, Zhu X (2013c) Short-term effect of nitrogen addition on litter and soil properties in Calamagrostis angustifolia freshwater marshes of northeast China. Wetlands, **33**, 505-513.

Song Y, Song C, Li Y, Hou C, Yang G, Zhu X (2013d) Short-term effects of nitrogen addition and vegetation removal on soil chemical and biological properties in a freshwater marsh in Sanjiang Plain, Northeast China. Catena, **104**, 265-271.

Spinnler D, Egli P, Körner C (2002) Four-year growth dynamics of beech-spruce model ecosystems under CO2 enrichment on two different forest soils. Trees, **16**, 423-436.

Tanner E, Kapos V, Franco W (1992) Nitorgen and Phosphorus Fertilization Effects on Venezuelan Montane Forest Trunk Growth and Litterfall. Ecology, **73**, 78-86.

Thirukkumaran CM, Parkinson D (2002) Microbial activity, nutrient dynamics and litter decomposition in a Canadian Rocky Mountain pine forest as affected by N and P fertilizers. Forest Ecology and Management, **159**, 187-201.

Thormann MN, Bayley SE (1997) Response of aboveground net primary plant production to nitrogen and phosphorus fertilization in peatlands in southern boreal Alberta, Canada. Wetlands, **17**, 502-512.

Torn MS, Vitousek PM, Trumbore SE (2005) The influence of nutrient availability on soil organic matter turnover estimated by incubations and radiocarbon modeling. Ecosystems, **8**, 352-372.

Tripathi S, Kushwaha C, Singh K (2008) Tropical forest and savanna ecosystems show differential impact of N and P additions on soil organic matter and aggregate structure. Global Change Biology, **14**, 2572-2581.

Tu L, Hu T, Zhang J, He Y, Tian X, Xiao Y, Jing J (2010) Effects of simulated nitrogen deposition on soil active carbon pool and root biomass in Neosinoca lamusaffinis plantation, rainy area of West China. Sheng Tai Xue Bao, **30**, 2286-2294.

Tu L, Hu T, Zhang J, Li R, Dai H, Luo S (2011a) Response of soil organic carbon and nutrients to simulated nitrogen deposition in Pleioblastus amarus plantation, Rainy Area of West China. Zhi Wu Sheng Tai Xue Bao, **35**, 125-136.

Tu LH, Hu TX, Zhang J, Li RH, Dai HZ, Luo SH (2011b) Short-term simulated nitrogen deposition increases carbon sequestration in a Pleioblastus amarus plantation. Plant and soil, **340**, 383-396.

Tu LH, Hu TX, Zhang J, Li XW, Hu HL, Liu L, Xiao YL (2013) Nitrogen addition stimulates different components of soil respiration in a subtropical bamboo ecosystem. Soil Biology and Biochemistry, **58**, 255-264.

Tu Y, You YM, Sun JX (2012) Effects of forest floor litter and nitrogen addition on soil microbial biomass C and N and microbial activity in a mixed Pinus tabulaeformis and Quercus liaotungensis forest stand in Shanxi Province of China. Ying yong sheng tai xue bao, **23**, 2325-2331.

Turner C, Knapp A (1996) Responses of a C4 grass and three C3 forbs to variation in nitrogen and light in tallgrass prairie. Ecology, 1738-1749.

Turner CL, Blair JM, Schartz RJ, Neel JC (1997) Soil N and plant responses to fire, topography, and supplemental N in tallgrass prairie. Ecology, **78**, 1832-1843.

Van Duren I, Boeye D, Grootjans A (1997) Nutrient limitations in an extant and drained poor fen: implications for restoration. Plant Ecology, **133**, 91-100.

Van Wijnen H, Bakker J (1999) Nitrogen and phosphorus limitation in a coastal barrier salt marsh: the implications for vegetation succession. Journal of Ecology, **87**, 265-272.

Venterink HO, Van Der Vliet R, Wassen M (2001) Nutrient limitation along a productivity gradient in wet meadows. Plant and soil, **234**, 171-179.

Verburg PS, Arnone JA, Obrist D *et al.* (2004) Net ecosystem carbon exchange in two experimental grassland ecosystems. Global Change Biology, **10**, 498-508.

Verhoeven J, Schmitz M (1991) Control of plant growth by nitrogen and phosphorus in mesotrophic fens. Biogeochemistry, **12**, 135-148.

Volk M, Obrist D, Novak K, Giger R, Bassin S, Fuhrer J (2011) Subalpine grassland carbon dioxide fluxes indicate substantial carbon losses under increased nitrogen deposition, but not at elevated ozone concentration. Global Change Biology, **17**, 366-376.

Vose JM, Elliott KJ, Johnson DW, Walker RF, Johnson MG, Tingey DT (1995) Effects of elevated CO2 and N fertilization on soil respiration from ponderosa pine (Pinus ponderosa) in open-top chambers. Canadian Journal of Forest Research, **25**, 1243-1251.

Waldrop MP, Zak DR, Sinsabaugh RL (2004a) Microbial community response to nitrogen deposition in northern forest ecosystems. Soil Biology and Biochemistry, **36**, 1443-1451.

Waldrop MP, Zak DR, Sinsabaugh RL, Gallo M, Lauber C (2004b) Nitrogen deposition modifies soil carbon storage through changes in microbial enzymatic activity. Ecological Applications, **14**, 1172-1177.

Wallenstein MD, Mcnulty S, Fernandez IJ, Boggs J, Schlesinger WH (2006) Nitrogen fertilization decreases forest soil fungal and bacterial biomass in three long-term experiments. Forest Ecology and Management, **222**, 459-468.

Wang H, Mo JM, Lu XK, Xue JH, Li J, Fang Y (2008a) Effects of elevated nitrogen deposition on soil microbial biomass carbon in the main subtropical forests of southern China. Sheng Tai Xue Bao, **28**, 470-478.

Wang J, Zhu T, Ni H, Zhong H, Fu X, Wang J (2013) Effects of elevated CO2 and nitrogen deposition on ecosystem carbon fluxes on the sanjiang plain wetland in Northeast China. PloS one, **8**, e66563.

Wang L, D’odorico P, O’halloran LR, Caylor K, Macko S (2010) Combined effects of soil moisture and nitrogen availability variations on grass productivity in African savannas. Plant and soil, **328**, 95-108.

Wang Q, Wang S, Liu Y (2008b) Responses to N and P fertilization in a young Eucalyptus dunnii plantation: Microbial properties, enzyme activities and dissolved organic matter. Applied Soil Ecology, **40**, 484-490.

Wang Z, Hao X, Shan D *et al.* (2011) Influence of increasing temperature and nitrogen input on greenhouse gas emissions from a desert steppe soil in Inner Mongolia. Soil Science and Plant Nutrition, **57**, 508-518.

West JB, Hobbie SE, Reich PB (2006) Effects of plant species diversity, atmospheric [CO2], and N addition on gross rates of inorganic N release from soil organic matter. Global Change Biology, **12**, 1400-1408.

Williams B, Silcock D (1997) Nutrient and microbial changes in the peat profile beneath Sphagnum magellanicum in response to additions of ammonium nitrate. Journal of Applied Ecology, 961-970.

Xu W, Wan S (2008) Water-and plant-mediated responses of soil respiration to topography, fire, and nitrogen fertilization in a semiarid grassland in northern China. Soil Biology and Biochemistry, **40**, 679-687.

Xu X, Ouyang H, Cao G, Pei Z, Zhou C (2004) Nitrogen deposition and carbon sequestration in alpine meadows. Biogeochemistry, **71**, 353-369.

Yan L, Chen S, Huang J, Lin G (2010) Differential responses of auto - and heterotrophic soil respiration to water and nitrogen addition in a semiarid temperate steppe. Global Change Biology, **16**, 2345-2357.

Yang XX, Ren F, Zhou HK (2014) Responses of plant community biomass to nitrogen and phosphorus additions in an alpine meadow on the Qinghai-Xizang Plateau. Zhi Wu Sheng Tai Xue Bao, **38**, 159-166.

Yano Y, Mcdowell W, Aber J (2000) Biodegradable dissolved organic carbon in forest soil solution and effects of chronic nitrogen deposition. Soil Biology and Biochemistry, **32**, 1743-1751.

Yu PY, Zhu F, Su SF, Wang ZY, Yan WD (2013) Effects of nitrogen addition on red soil microbical in the Cinnamomum camphora plantation. Huan Jing Ke Xue, **34**, 3231-3237.

Zhang JZ, Ni HW, Wang JB, Yuan L, Wang HT (2013a) Effects of simulated nitrogen deposition and elevated CO2 concentration on soil organic carbon and nitrogen of deyeuxia angustifolia community on the Sanjiang Plain. Di Qiu Yu Huan Jing, **41**, 216-225.

Zhang L, Song C, Nkrumah PN (2013b) Responses of ecosystem carbon dioxide exchange to nitrogen addition in a freshwater marshland in Sanjiang Plain, Northeast China. Environmental

Pollution, **180**, 55-62.
Zhang N, Wan S, Li L, Bi J, Zhao M, Ma K (2008) Impacts of urea N addition on soil microbial community in a semi-arid temperate steppe in northern China. Plant and soil, **311**, 19-28.
Zhou X, Zhang Y, Downing A (2012) Non-linear response of microbial activity across a gradient of nitrogen addition to a soil from the Gurbantunggut Desert, northwestern China. Soil Biology and Biochemistry, **47**, 67-77.
